# Mechanistic study of Jiawei Zicao Plaster in atopic dermatitis via IL-17 signaling pathway and skin microbiome modulation

**DOI:** 10.3389/fmicb.2025.1668089

**Published:** 2025-09-24

**Authors:** Andong Wang, Tingting Chen, Hongtao Zhang, Yuru Yang, Xiaotian Cheng, Di Chen, Bai Ling

**Affiliations:** ^1^School of Pharmacy, Nantong University, Nantong, Jiangsu, China; ^2^Department of Pharmacy, The Yancheng Clinical College of Xuzhou Medical University & The First people's Hospital of Yancheng, Yancheng, Jiangsu, China; ^3^Scientific Research Department, Ningbo Municipal Hospital of Traditional Chinese Medicine & Affiliated Hospital of Zhejiang Chinese Medical University, Ningbo, Zhejiang, China

**Keywords:** Jiawei Zicao Plaster, atopic dermatitis, network analysis, inflammation, skin microbiota

## Abstract

**Background:**

Atopic dermatitis (AD) is a prevalent and difficult-to-cure chronic inflammatory skin condition. The mechanism of Jiawei Zicao Plaster (JZP), a clinically used Traditional Chinese Medicine (TCM) for AD, remains incompletely understood.

**Methods:**

Following successful model induction, JZP was topically applied to the dorsal skin of mice. Therapeutic efficacy was evaluated through histopathological examination of skin sections and quantification of inflammatory biomarkers using ELISA. To investigate the underlying mechanisms, we performed qRT-PCR to analyze gene expression in related signal pathway, along with 16S rRNA sequencing to characterize skin microbiota composition.

**Results:**

Network pharmacological analysis revealed that JZP may exert therapeutic effects by targeting antibacterial and anti-inflammatory pathways. Subsequent *in vivo* experiments demonstrated that JZP effectively ameliorates biochemical markers of AD by activating the IL-17 pathway. Analysis of skin microbiota indicated that JZP treatment led to an increase in *Chryseobacterium* abundance and a decrease in *Staphylococcus* abundance.

**Conclusion:**

This study seeks to validate the efficacy of JPZ as a treatment for AD. Initial findings suggest that JPZ attains its therapeutic efficacy through the synergistic actions of IL-17 and antibacterial properties, providing a theoretical basis for the future development and application of this traditional Chinese medicine formula.

## Graphical Abstract

The mechanism of action of Jiawei Zicao Plaster on atopic dermatitis. 
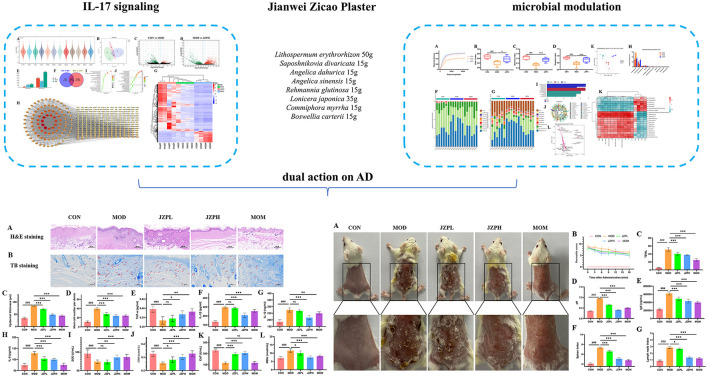

## 1 Introduction

Atopic dermatitis (AD) is a prevalent chronic inflammatory skin condition that affects 5–8% of adults and 11–20% of children. The complexity of AD is increased by the different variations in the AD endotype (Schuler et al., 2023). According to clinical criteria (disease stage/chronicity), demographics (age, ethnicity), and molecular parameters/endotypes, this condition has multiple subgroups. Data from the European Community Respiratory Health Survey (ECRHS) on subjects from the United States and Europe reveal a notable disparity in adult prevalence rates of AD between countries, with Switzerland reporting the lowest rate at 0.3% and Estonia the highest at 6.2%. Additionally, AD appears to be more prevalent among females than males. The peak prevalence is observed in the 25–45 age group, followed by a gradual decline with advancing age (*p* < 0.05). The development of a novel therapeutic method based on the AD subtype is necessary to attain the therapeutic benefits that the patient anticipates, even though some people can be treated with traditional medicines. Severe pruritus and recurrent eczematous lesions negatively affect the patient's psychological and social wellbeing and also significantly burden caregivers, partners, and close family members (Ali et al., 2020). The human skin, the body's largest organ, serves as the primary defense against environmental elements. Skin microorganisms engage with the skin beyond surface adhesion, influencing its barrier function. Disruption of the skin microbiome can precipitate various skin conditions, including AD and psoriasis (Lee and Kim, 2022; Prajapati et al., 2025).

Glucocorticoids, immunosuppressants, and calcium phosphatase inhibitors are being utilized to treat AD (Sidbury et al., 2023). These medications are given as topical and systemic emollients. Because of the severe side effects, resistance to glucocorticoid and antibiotic therapy, and potential for rebound, experts from the International Eczema Committee regrettably advise against using glucocorticoids and antibiotics for AD, even though they are widely used clinically. Patients and physicians are looking for complementary and alternative medications to treat AD in light of these possible negative effects. One possible source is Chinese botanical medication that is both safe and effective (Thyssen et al., 2023).

Traditional Chinese medicine (TCM) has demonstrated unique therapeutic advantages in this field. An empirical formula, Jiawei Zicao Plaster (JZP, *Lithospermum erythrorhizon* Siebold & Zucc. [Boraginaceae *Lithospermum*; *Lithospermum erythrorhizon* radix et rhizoma] 500 g, *Saposhnikovia divaricata* (Turcz. ex Ledeb.) Schischk. [Apiaceae *Saposhnikovia*; *Saposhnikovia divaricate* radix et rhizoma] 150 g, *Angelica dahurica* (Hoffm.) Benth. & Hook.f. ex Franch. & Sav. [Apiaceae *Angelica*; *Angelica dahurica* radix et rhizoma] 150 g, *Angelica sinensis* (Oliv.) Diels [Apiaceae *Angelica*; *Angelica sinensis* radix et rhizoma] 150 g, *Rehmannia glutinosa* (Gaertn.) Libosch. ex Fisch. & C. A. Mey. [Orobanchaceae *Rehmannia*; *Rehmannia glutinosa* radix et rhizoma] 150 g, *Lonicera japonica* Thunb. [Caprifoliaceae *Lonicera*; *Lonicera japonica* flower buds] 350 g, *Commiphora myrrha* (Nees) Engl. [Burseraceae *Commiphora*; *Commiphora myrrha* resin] 150 g, *Boswellia carterii* Birdw. [Burseraceae *Boswellia*; *Boswellia carterii* resin] 150 g), was developed through a combination of addition and subtraction based on the classic formula “Zicao Plaster” from the Ming Dynasty's “Wai Dao Zheng Zong” (Oh et al., 2021). This TCM formulation has shown substantial clinical benefits in managing atopic dermatitis, including lesion resolution and symptom relief, though its exact molecular targets and mechanisms of action require further investigation. Traditional Chinese medicine formulations are crucial in managing inflammatory skin conditions, notably AD. For instance: three-herbs formula markedly decreased IL-6 and tumor necrosis factor (TNF)-α production in HMC-1 cells. It also suppressed the expression of IL-6, IL-8, and CCL2 in TNF-α/IFN-γ-stimulated HaCaT cells, and inhibited nitric oxide (NO) production induced by lipopolysaccharide (LPS) in RAW 264.7 cells. Additionally, it significantly reduced the production of CCL2 and TNF-α in LPS-induced RAW 264.7 cells. In an *in vivo* model, the formula alleviated ear swelling and scratching frequency in mice, reduced epidermal thickness, and inhibited mast cell infiltration (Wu et al., 2021).

Thus, the purpose of this study was to use network analysis, transcriptomics, quantitative real-time reverse transcription polymerase chain reaction (qRT-PCR) and experimental validation to examine the therapeutic impact of JZP extracts on AD mice. The balance between IL-17-mediated immune responses and cutaneous microbial composition appears to be reciprocally regulated, based on experimental validation. These results imply that JZP administered for cutaneous application is a successful treatment strategy for AD. We propose that JZP could be used as an alternate therapy to treat AD. It might also provide important information on the pathophysiology of AD and the creation of novel treatment agents.

## 2 Materials and methods

### 2.1 Gene identification of JZP

The traditional Chinese medicine system pharmacology database and analysis platform (TCMSP) (Ru et al., 2014; Gu et al., 2024; Fan et al., 2022) was used to screen the metabolites and genes of JZP (*Lithospermum erythrorhizon, Saposhnikovia divaricata, Angelica dahurica, Angelica sinensis, Rehmannia glutinosa, Lonicera japonica, Commiphora myrrha, Boswellia carterii*). The degree to which a medication reaches the bloodstream and has biological effects following oral delivery is known as oral bioavailability, or OB. A medication with a high oral bioavailability is more readily absorbed and acts on the body through the oral route. Consequently, a criterion of OB ≥30% aids in the exclusion of additional possible therapeutic molecules having a higher oral bioavailability following oral delivery. The term “drug-likeness” (DL) describes how well a metabolite's physicochemical characteristics match those of drug molecules in general. In terms of size, hydrophilicity, chemical stability, etc., drug molecules generally have some characteristics. A molecule with a higher degree of drug-likeness is more likely to have favorable pharmacological and toxicological characteristics and to develop into a candidate medication. To guarantee uniformity between datasets, the Uni Prot database was used to standardize the gene symbols for every gene that was found (Ru et al., 2014).

### 2.2 Gene identification associated with AD

Afterwards, we used five databases to look for genes linked to AD: OMIM (Amberger et al., 2015), DrugBank (Wishart et al., 2018), DisGeNet (Piñero et al., 2017), Genecards (Rebhan et al., 1997), and TTD (Zhou et al., 2024). A comprehensive gene collection associated with AD was created by combining the search results from numerous databases. We constructed an interactive venny diagram of JZP metabolites genes and AD disease genes, and obtained the intersection of metabolites and disease genes.

### 2.3 PPI network analysis

The STRING database, the gene set for metabolites, and the gene set associated with AD were used to construct a protein-protein interaction (PPI) network using a set parameter of moderate confidence (0.400). STRING was used to export the PPI network, and Cytoscape was used to further analyze the significant sub-network. CytoNca scores were used to filter genes with Betweenness, Closeness, and Degree values higher than the median. These filtered genes were used to build a primary sub-network, and the primary sub-network was further filtered to generate a final, crucial sub-network.

### 2.4 GO and KEGG analysis

Using the metabolites gene set and the AD-related gene set in Cytoscape version 3.10.2, we built a metabolite -gene network. Then, utilizing Kyoto Encyclopedia of Genes and Genomes (KEGG) pathway analysis and gene ontology (GO), enrichment analysis was used to identify the underlying biological processes (BP), cellular components (CC), molecular functions (MF), and important signaling pathways. The Metascape database was used in this analysis.

### 2.5 Preparation of JZP

All materials for the experiment were procured from Beijing Tongrentang. The herbs were processed as follows: The resin of *C. myrrha*, and *B. carterii*, 150 g each, were finely ground and sieved. Additionally, the root of *S. divaricata*, the root of *A. dahurica*, the root of *A. sinensis*, the root of *R. glutinosa* (150 g each), and the rattan of *L. japonica* (350 g) were cut into pieces. These materials were dissolved in 6,000 g of edible sesame oil, heated until dry, and then filtered. The whole grass of *L. erythrorhizon* was added to the solution and heated until it turned purplish-red. After filtering out the materials, JPZ was obtained. UPLC-Q-TOF/MS identified shikonin as the main component of JPZ, meeting the Chinese Pharmacopeia standards (Supplementary Figure S1 and Supplementary Table S1).

### 2.6 Administration of JZP in AD models

AD mice were created in accordance with earlier research. In short, an electric shaver was used to shave a 2 cm × 2 cm section of dorsal. After sensitization, 100 μl 0.5% DNCB was applied for the first 3 days, and then every other 2 days, 100 μl 0.3% DNCB. In the present study, the sample size was calculated using G^*^power analysis. The mice in the control group received the same vehicle (acetone/olive 3:1, vol/vol). The control group (CON), AD model group (MOD), JZP-treated groups (150, 300 mg/kg), and positive group (Mometasone Furoate Cream, MOM) make up the five groups (*n* = 8). Typical clinical dosages are 40.0 g of crude herbs/person/d for AD. The dosing protocol for the animal model was established via interspecies dose translation from humans (65 kg body weight) to mice at a 1:12.3 ratio. The low-dose group was calibrated to approximate the human equivalent dose, while the high-dose group was set at twice this amount. The low-dosage (150 mg/kg) was selected based on prior studies. The mice in the JZP-treated and positive groups received topical treatment daily. The AD model group and the control group received the same quantity of vehicles. Daily observations were made of the skin lesion characteristics, which included crusts, excoriations, edema, and erythema. In order to undertake an objective scoring system, the mice's AD model's scoring criteria were based on the severity of the clinical symptoms. Clinical scores of 0 (no symptoms), 1 mild, 2, moderate, and 3 severe were noted. Dorsal skin was separated and blood was collected at the conclusion of the experiment. A Gpskin barrier (GPskin, Seoul, Republic of Korea), a tool used to measure the amount of water that evaporates from the skin's surface, was used to measure transepidermal water loss (TEWL). To guarantee precision and consistency, measurements were made in the middle of the lesioned area. In order to prevent pressure from changing the skin's normal TEWL values, the device was gently placed on the skin. To reduce variability and guarantee data dependability, readings were obtained from each mouse at least twice. The skin's capacity to retain moisture and act as an efficient barrier was quantified by measuring TEWL values, which were expressed in grams per hour per square meter (g/h/m^2^). All mice were anesthetized with 2%−3% isoflurane inhaled for 2–3 min and then sacrificed by rapid cervical dislocation to avoid unnecessary pain and suffering before death. The animal ethical and welfare was approved on April 25th in 2024 with an Approve No. S20240927-004.

### 2.7 Histopathological analysis

Once a 24-h grace period from the last product application, mice were culled once the JZP treatment regimen was stopped. After carefully removing the dorsal skin and ear tissues, they were immediately preserved in 10% (v/v) formalin for 48 h. Following fixation, ethanol concentrations ranging from 70% to 100% were used in a graduated dehydration procedure on the tissues. The tissues were then sectioned at a thickness of 5 μm, fixed in paraffin, and prepared for staining. Following thorough deparaffinization and rehydration of the samples, hematoxylin and eosin (H&E) staining was used to examine alterations in epidermal hyperplasia and inflammatory cell infiltration. To determine the extent of mast cell infiltration in the dorsal skin samples, Toluidine Blue (TB) staining was used. All tissue sections were carefully examined under light microscopy after staining in order to record any histological changes. Images were obtained at a magnification of × 200 and subjected to morphometric analysis using Zeiss image analysis software in order to provide a quantitative interpretation of the histological changes.

### 2.8 Serum biochemical analysis

The mice were euthanized after the treatment period and the development of AD-like lesions, and serum separator tubes were used to collect blood samples. After 30 min of clotting at room temperature, the blood was centrifuged for 20 min at 1,500 × g. The clotted blood and serum were separated by this method. The total serum biochemical concentration was determined using a sandwich enzyme-linked immunosorbent assay (ELISA) kit designed for mouse immunoglobulin E (lgE), Tumor Necrosis Factor-alpha (TNF-α), interleukin-1β (IL-1β), interleukin-6 (IL-6), interleukin-10 (IL-10), superoxide dismutase (SOD), glutathione (GSH), Catalase (CAT), malondialdehyde (MDA) (Nanjing Jiancheng Technology Co., Ltd.).

### 2.9 qRT-PCR analysis

Total RNA was extracted from skin tissue using the Trizol reagent (Beyotime Biotechnology Co., China). Following the quantification of total RNA using NanoDrop 2000 (Thermo Fisher, USA), the RNA was reverse-transcribed into cDNA using a kit (Cwbio Century Biotechnology Co., China). The cDNA was subsequently amplified using a Quant Studio3 fluorescent quantitative PCR instrument in compliance with the SYBR kit (Cwbio Century Biotechnology Co., China). After 10 min at 95 °C, the PCR reaction was performed 40 times, with 15 s at 95 °C and 1 min at 60 °C. The comparison method (2−ΔΔCt) used to determine relative mRNA levels used GAPDH as an internal reference gene. The primer sequences used were listed in Table 1 (Huada Gene Research Institute, China).

**Table 1:** Primer sequences for qPCR.

**Primer name**	**Primer sequence (5^′^-3^′^)**	**Primer length/bp**
HSP90AA1	F GACGCTCTGGATAAAATCCGTT	22
	R TGGGAATGAGATTGATGTGCAG	22
JUN	F TTCCTCCAGTCCGAGAGCG	19
	R TGAGAAGGTCCGAGTTCTTGG	21
STAT1	F TCACAGTGGTTCGAGCTTCAG	21
	R CGAGACATCATAGGCAGCGTG	21
IL6	F CTGCAAGAGACTTCCATCCAG	21
	R AGTGGTATAGACAGGTCTGTTGG	23
IL2	F TGAGCAGGATGGAGAATTACAGG	23
	R GTCCAAGTTCATCTTCTAGGCAC	23
NFKBIA	F TGAAGGACGAGGAGTACGAGC	21
	R TGCAGGAACGAGTCTCCGT	19
IL1B	F GAAATGCCACCTTTTGACAGTG	22
	R TGGATGCTCTCATCAGGACAG	21
TGFB1	F CCACCTGCAAGACCATCGAC	20
	R CTGGCGAGCCTTAGTTTGGAC	21

### 2.10 Transcription analysis

To create clean reads appropriate for additional research, the raw sequencing data underwent a rigorous cleaning step. First, reads with a low-quality base ratio (quality score ≤ 15) greater than 40%, reads containing more than 1% of unknown bases, and reads tainted by adaptor sequences were filtered out using SOAPnuke (version 1.6.5). The FASTQ format was then used to store the clean reads. Bowtie2 (version 2.4.5) was then used to map the cleaned data to the generated unique gene sequences. After mapping, RSEM (version 1.3.1) was used to accurately quantify the gene expression levels. Public databases like KEGG and GO have been used to annotate the genes. Important details regarding the biological functions of the genes were revealed by this annotation process. Genes that exhibit differential expression between groups with fold change (FC) > 2 or < 0.5 were identified using DESeq2. This was done to make sure that the detection rate of DEGs is more than or equal to 66.6% in at least one group and to remove differentially expressed genes (DEGs) with fragments per kilobase of transcript per million fragments mapped (FPKM) values less than 1 in both comparison groups. The DEGs were functionally classified using the KEGG and GO annotations.

### 2.11 Skin microbiota analysis

Fresh skin samples were collected in liquid nitrogen for high-throughput sequencing analysis in accordance with previous protocols (Wang et al., 2022). Principal coordinate analysis (PCoA) and cluster analysis were used to calculate feature-level alpha diversity indices for the beta diversity study. The Chao1 richness estimation, the Shannon diversity index, and observed OTUs were used. The LEfSe application was used to analyze the differences in significance between several groups. Spearman's correlations were used to determine the relationship between the skin microbiota and inflammatory indicators.

### 2.12 Statistical analysis

The data was evaluated using the SPSS 22.0 program. The findings were displayed using the mean ± standard deviation. Group comparisons were created using a one-way ANOVA and the Tukey post-test; a *p*-value of less than 0.05 was deemed statistically significant.

## 3 Results

### 3.1 Network analysis analysis of JZP

The TCMSP database and Swiss Gene Prediction database was used to search and predict the metabolites and metabolite-related genes of JZP, a total of 106 metabolites and 1,422 genes were obtained: *L. erythrorhizon* with 11 metabolites and 98 genes; *S. divaricata* with 17 metabolites and 229 genes; *A. dahurica* with 20 metabolites and 156 genes; *A. sinensis* with 2 metabolites and 43 genes; *R. glutinosa* with 2 metabolites and 33 genes; *L. japonicae* with 15 metabolites and 256 genes; *C. myrrha* with 33 metabolites and 377 genes; *B. carterii* with 6 metabolites and 15 genes.

**Figure 1:**
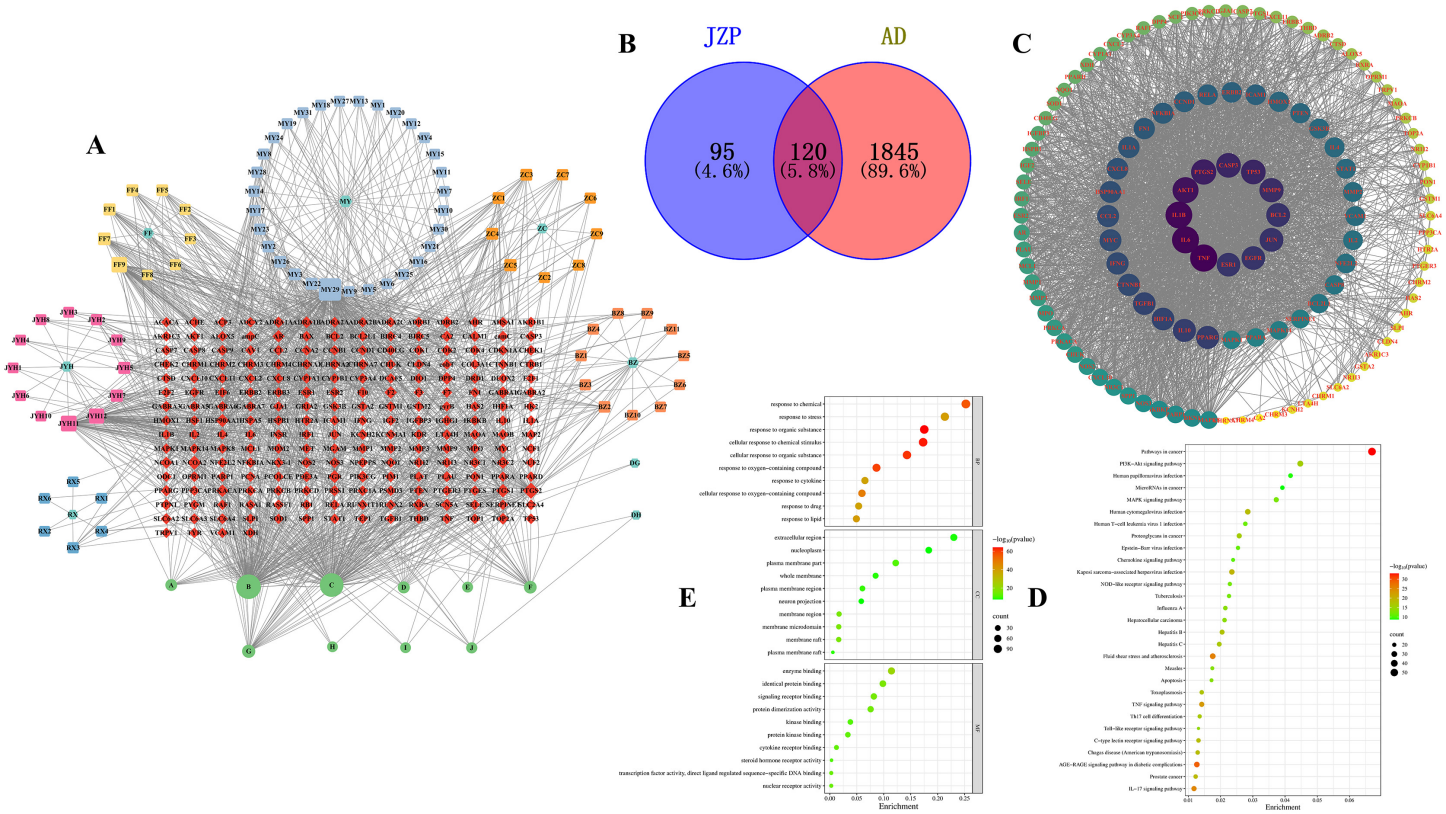
Network analysis of JZP. Gene-disease-pathway association network **(A)**, Venn diagram of metabolite genes of JZP and AD-related genes **(B)**, Core genes **(C)**, The KEGG pathway enrichment analysis of key genes **(D)**, GO pathway enrichment analysis of key genes **(E)**.

There are 215 shared genes and 10 common metabolites among these metabolites and associated genes. One thousand two hundred and seven distinct genes and 96 independent metabolites were obtained by combining and eliminating the duplicates (Figure 1A). Following this, genes linked to AD were analyzed using the OMIM, DrugBank, DisGeNet, Genecards, and TTD databases; 23, 66, 18, 1,906, and 20 AD-related genes were obtained, respectively. Deduplication and aggregation produced a total of 1,965 genes linked to AD. After that, 120 intersecting genes were found by calculating the intersection of medication and AD genes using Venny2.0 software (Figure 1B). The PPI network of the metabolite Chinese medicine-acting disorders was obtained by importing the 120 intersecting genes into the STRING database. This resulted in 120 nodes, 2,656 edges, and an average node degree value of 44.3, suggesting that the gene-encoded proteins had complicated interactions. The color hues in Figure 1C, which are imported into Cytoscape 3.10.2 software for analysis and visualization, indicate the strength of the correlation. The topological statistics of network analysis was presented in the Supplementary Table S2.

### 3.2 KEGG and GO analysis

A total of key pathways was identified by KEGG pathway analysis, and the results were plotted as bubble diagrams (Figure 1D). These pathways included several key biological signaling pathways and processes such as Pathways in cancer, PI3K-Akt signaling pathway, Fluid shear stress and atherosclerosis, AGE-RAGE signaling pathway in diabetic complications, Human cytomegalovirus infection, Kaposi sarcoma-associated herpesvirus infection, IL-17 signaling pathway, Human papillomavirus infection, Th17 cell differentiation, Tuberculosis, NOD-like receptor signaling pathway, Epstein-Barr virus infection, Apoptosis, Measles, Chemokine signaling pathway, Toll-like receptor signaling pathway. The pathways most closely associated with atopic dermatitis are IL-17 and Th17 cell differentiation signaling pathways. Consequently, the IL-17 pathway, ranked highest, was chosen as the focus for further research.

The genes of the metabolite botanical medicine for AD were entered into the METASCAPE database and analyzed by GO functional enrichment, and 10 closely related BP were obtained, covering a wide range of response to organic substance, cellular response to chemical stimulus, cellular response to organic substance, response to oxygen-containing compound, response to chemical, cellular response to oxygen-containing compound, response to lipid, response to stress, response to cytokine and response to drug. The analysis also identified 10 major MF and 10 CC. We plotted the BP, CC, and MF obtained from the GO functional analysis as bubble diagrams (Figure 1E).

Most significantly, our results suggest that JZP may play a part in regulating several important immune system and inflammatory response pathways. JZP appears to have an impact on a number of important functions, including immune effector process modulation and inflammatory response regulation. Additionally, the activity of the inflammatory cytokine IL-17, which is essential for immunological and inflammatory responses, is positively modulated by JZP. The idea that JZP may have therapeutic potential in the treatment of several skin disorders is supported by this preliminary investigation. These results further illustrate the intricacy of JZP's biological activity and the value of a systems biology viewpoint in comprehending its mechanisms of action.

### 3.3 Alleviated AD-like symptoms by JZP

**Figure 2:**
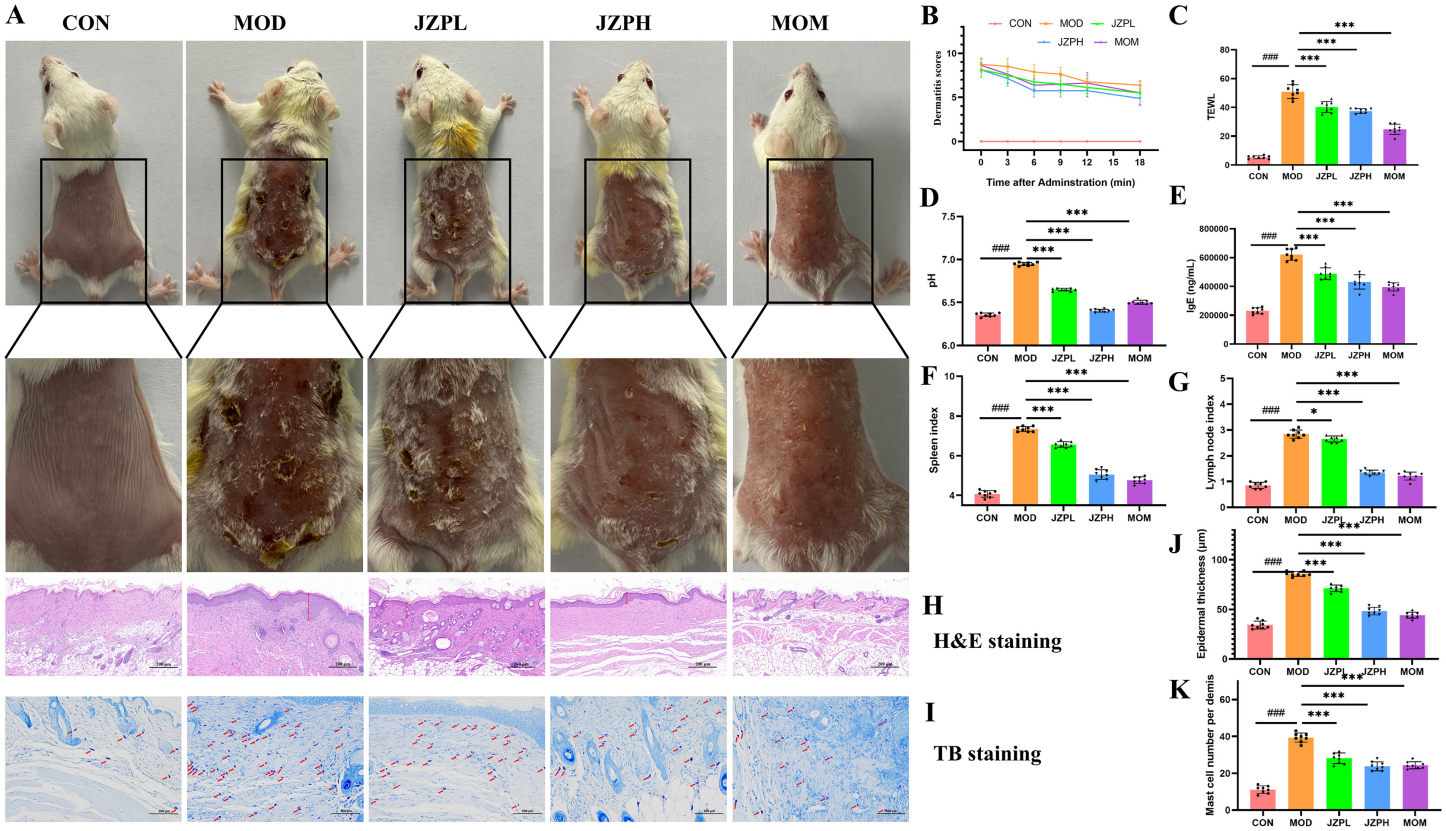
Effects of JZP on DNCB-induced mice. Images of dorsal skin of DNCB-induced AD mice **(A)**, Dermatitis scores **(B)**, TWEL **(C)**, Ph **(D)**, the level of IgE **(E)**, Spleen index **(F)**, Lymph node index **(G)**, HandE staining **(H)**, TB staining **(I)**, Statistical analysis of epidermal thickness **(J)**, Statistical analysis of mast cell numbers **(K)**. Magnification at 200X, Bar = 100 μm, red arrows indicate mast cells (dark purple red). *represents for vs. MOD group (*p* < 0.05), ***represents for vs. MOD group (*p* < 0.001), ###represents for vs. CON group (*p* < 0.001), respectively (*n* = 6).

Photographic evidence of the skin lesions demonstrated that topical DNCB administration resulted in atopic dermatitis-like signs and symptoms. In comparison to the control group that was not exposed to DNCB, these symptoms—which included extreme erythema, erosion, dryness, and a noticeable scratching behavior—led to a noticeably higher dermatitis score. According to our research, JZP administration significantly reduces the dermatitis score, and this anti-inflammatory action exhibits a clear dose-dependent pattern (Figures 2A, B).

An important first step in comprehending JZP's potential as a treatment for atopic dermatitis (AD) is assessing its impact on cutaneous epidermal lichenification. Chronic AD is typified by lichenification, which is characterized by thicker skin, more wrinkles, and hyperpigmentation. This disorder is produced by the complex interaction of several variables, including changed skin lipid composition, decreased moisture retention, ceramide shortages, impaired filaggrin function, and abnormalities in skin pH levels. JZP can control keratin synthesis and greatly reduce transepidermal water loss (TEWL), which offers a dual approach for reducing the pathogenic processes causing lichenification (Figure 2C). In order to treat the primary symptoms of lichenification and restore the integrity of the epidermal barrier, JZP can help regulate skin pH disturbances (Figure 2D). This is essential for reducing the vulnerability of lichenified skin to Staphylococcus aureus invasion and colonization, which is a common aggravating factor in AD.

Importantly, our study showed that the administration of JZP could effectively counteract this immunological perturbation. As shown in Figure 2E, the immunoglobulin level in the JZP group decreased compared with the MOD group, and the immunoglobulin level in the JZPH group tends to approach that in the MOM group. Serum IgE is a crucial clinical marker that is frequently elevated in AD cases, and its increased serum concentration is frequently observed in patients with AD. In our experimental model, repeated topical administration of DNCB caused a noticeable surge in serum IgE levels, mimicking the characteristic systemic immunological response in human AD. Specifically, JZP administration caused a dose-dependent drop in serum IgE levels. In addition to this, compared with the MOD group, the spleen index, lymph node index of the skin surface of the JZP group mice reduced, and the changes of the immune organ index at a high dose were close to the MOM group (Figures 2F, G).

### 3.4 Histopathological analysis

According to the H&E staining, the mice in the MOD group had thicker epidermis, intercellular edema, and eosinophil aggregation around superficial dermal blood vessels, which are pathological characteristics of AD, in contrast to the CON group (Figure 2H). The JZP intervention considerably decreased the epidermal thickness of the mice's back skin (Figure 2J) when compared to the MOD group (*p* < 0.001).

The TB staining reveals that the mice in the MOD group had mast cell infiltration in their epidermis as compared to the CON group (Figure 2I). The JZP intervention dramatically decreased number of mast cell (Figure 2K) (*p* < 0.001) as compared to the MOD group. These results suggest that JZP can successfully stop the dermatological alterations caused by DNCB that resemble AD.

### 3.5 Anti-inflammatory effects of JZP

**Figure 3:**
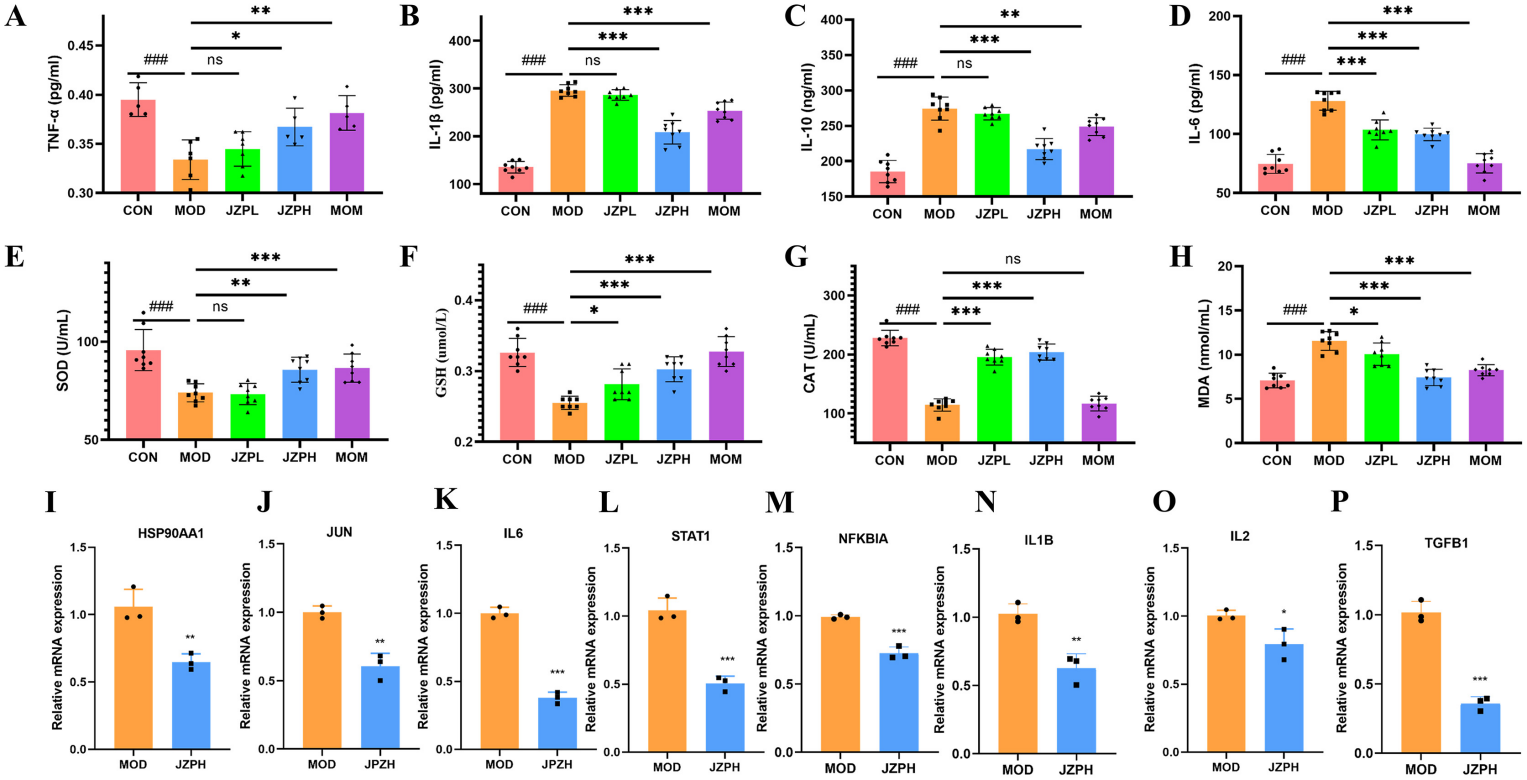
TNF-α **(A)**, IL-1β **(B)**, IL-10 **(C)**, IL-6 **(D)**, SOD **(E)**, GSH **(F)**, CAT **(G)**, MOA **(H)** (*n* = 6), relative mRNA expression of HSP90AA1 **(I)**, JUN **(J)**, IL6 **(K)**, STAT1 **(L)**, NFKBIA **(M)**, IL1B **(N)**, IL2 **(O)**, TGFB1 **(P)** (*n* = 3). Magnification at 200X, Bar = 100 μm, red arrows indicate mast cells (dark purple red). *represents for vs. MOD group (*p* < 0.05), **represents for vs. MOD group (*p* < 0.01), ***represents for vs. MOD group (*p* < 0.001), ns represents for vs. MOD group (*p* ≥ 0.05), ###represents for vs. CON group (*p* < 0.001), respectively.

Experiments have shown that JZP does, in fact, prevent the blood from releasing pro-inflammatory proteins like TNF-α, IL-1β, IL-6, and IL-10. The model group's levels of inflammatory factors are significantly higher than those of the blank control group, as illustrated in Figures 3A–D. In contrast, the JZP group's levels of pro-inflammatory factors significantly decreased in comparison to the model group, and this decrease became more noticeable as concentrations increased.

### 3.6 Alleviated oxidative stress by JZP

We examine how JZP affects the activities of oxidative stress indicators, such as SOD, GSH, and CAT, in order to determine whether it can reduce oxidative stress in DNCB-induced skin inflammation. According to the research findings, JZP therapy increases the activity of the enzymes SOD, GSH, and CAT in the skin lesions caused by DNCB. Furthermore, compared to the positive medication clobetasol propionate, JZP has a significantly greater activating effect on peroxidase (Figures 3E–G). Furthermore, mice treated with DNCB show a considerable decrease in MDA activity in their lesional skin after receiving JZP treatment (Figure 3H). These findings imply that JZP treatment might prevent the oxidative damage on the skin brought on by DNCB.

### 3.7 qRT-PCR verification by JZPH

JZP does, in fact, block the IL-17 signaling pathway, as demonstrated by qRT-PCR tests. This includes the expression of markers associated with HSP90AA1, JUN, STAT1, IL6, IL2, NFKBIA, IL1B, and TGFB1. Relative mRNA expression levels in the model group are substantially higher than those in the JZPH group, as displayed in Figures 3I–P.

### 3.8 Transcriptomics analysis

**Figure 4:**
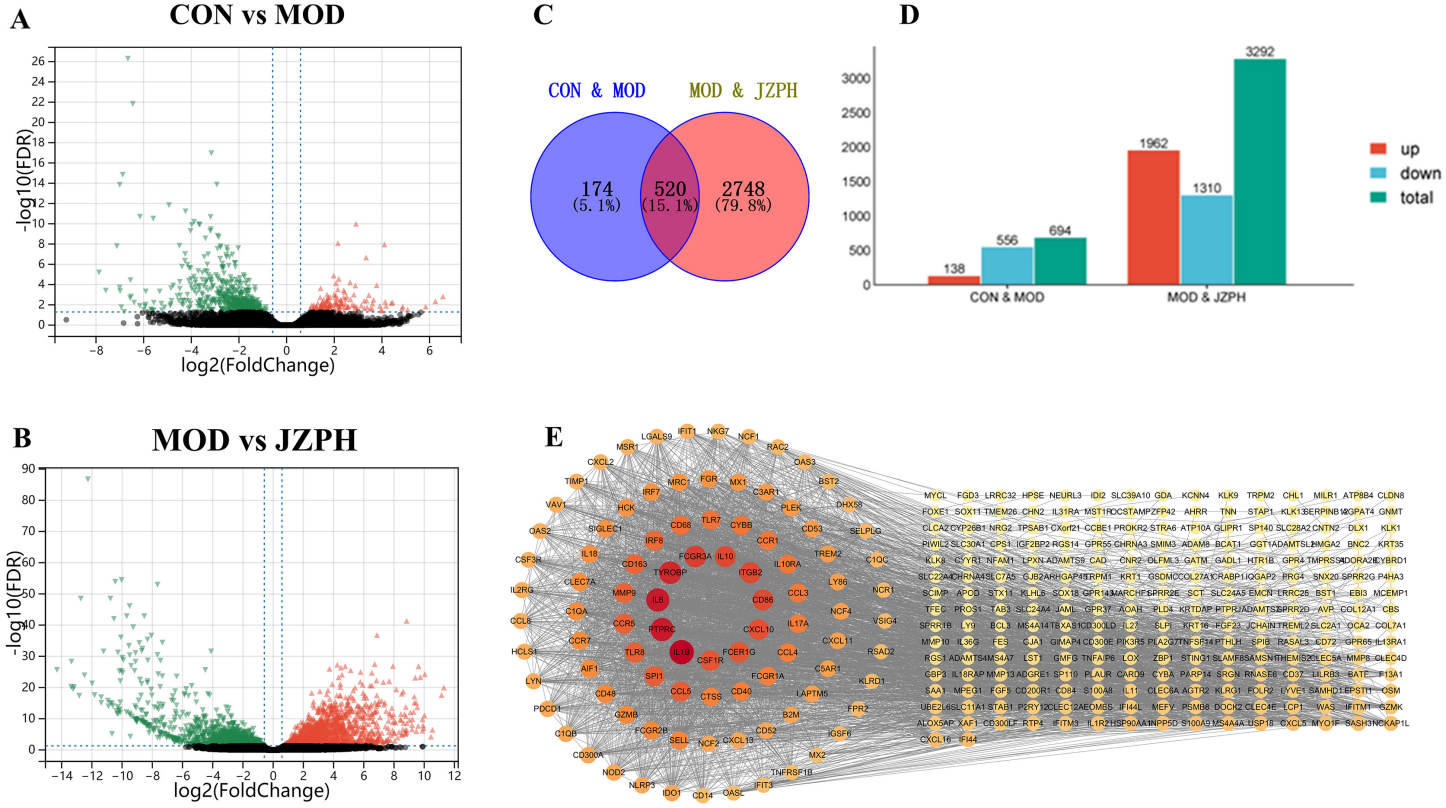
Expression difference volcano plot between CON vs. MOD **(A)**, Expression difference volcano plot between MOD vs. JZPH **(B)**, Venn diagram **(C)**, Expression difference statistic table **(D)**, PPI Network Analysis **(E)** (*n* = 3).

We used transcriptome analysis to assess the expression of inflammatory-related genes in the skin tissues of normal untreated mice, DNCB-treated animals, and JZPH-treated mice in order to look into the possible pathophysiology of DNCB-induced AD-like skin lesions. The distribution of gene expression levels in the CON, MOD, and JZPH groups was displayed using FPKM and PCA plots. The distribution of gene expression levels varied across these samples, according to the results (Supplementary Figures S2A, S2B). The level of variance in gene expression and its statistical significance were displayed using volcano and histogram plots. More genes were significantly down-regulated in the MOD group than in the CON group, and more genes were significantly up-regulated in the JZPH group than in the MOD group (Figures 4A, B). This suggests that JZPH may have a therapeutic effect on atopic dermatitis by influencing the expression of genes linked to inflammation and so regulating inflammatory-related pathways. Furthermore, we used a heatmap to display the expression level of the same gene across various groups (Supplementary Figure S2C). The findings further indicated that JZPH can exert anti-specific dermatitis effects by controlling gene expression.

The intersectional genes between the MOD *vs*. JZPH group and the CON *vs*. MOD group were then computed using Venny software, and a visual analysis was performed on them. According to the Venny plot, we found 520 similar values in all, which represents 15.1% of the entire gene pool (Figures 4C, D). Genes including IL6, IL1β, IL10, CD86, CXCL10, and others that are closely linked to atopic dermatitis were shown in the network plot; higher associations were indicated by darker colors (Figure 4E).

KEGG pathway analysis identified 20 key pathways, which were visualized as bubble plots (Supplementary Figure S2D). These pathways included several key biological signal transduction pathways and processes, such as cytokine-cytokine receptor interaction, chemokine signaling pathway, viral protein interaction with cytokine and cytokine receptor, tuberculosis, NOD-like receptor signaling pathway, phagosome, Toll-like receptor signaling pathway, osteoclast differentiation, Staphylococcus aureus infection, influenza A, FcγR-mediated phagocytosis, rheumatoid arthritis, pertussis, IL-17 signaling pathway, leishmaniasis, c-type lectin receptor signaling pathway, complement and coagulation cascades, inflammatory bowel disease (IBD), cytoplasmic DNA sensing pathway, and prion disease, among others.

Then, we obtained 10 closely related biological processes by GO functional enrichment analysis (Supplementary Figure S2E), covering various aspects such as immune system processes, immune response, defense response, response to external stimuli, regulation of immune system, cell activation, leukocyte activation, innate immune response, and regulation of immune response. And from the figure, it can be seen that the biological processes closely related to inflammation are immune system processes. GO functional enrichment analysis also identified 10 main molecular functions and 10 cellular components. Molecular functions mainly involved signal receptor binding, signal receptor activity, molecular transducer activity, receptor regulator activity, receptor ligand activity, cytokine receptor binding, cytokine activity, chemokine receptor binding, chemokine activity, and superoxide-generating NADPH oxidase activity. Cellular components mainly included the plasma membrane part, intracellular vesicle, cytoplasmic vesicle, cytoplasmic vesicle part, intrinsic component of the plasma membrane, secretory vesicle, secretory granule, cell surface, secretory granule membrane, and external side of plasma membrane, among others.

These results allow us to draw the preliminary conclusion that JZPH may be involved in several important immune system and inflammatory response regulatory pathways. It seems that JZPH affects a number of important functions, including immune response regulation, immunological effector regulation, and immune response regulation. Furthermore, it has been discovered that JZPH primarily regulates inflammatory responses by affecting the inflammatory IL-17 signaling pathway. Together with the findings of network analysis, these analytical data also offer a strong foundation for the theory that JZPH may have therapeutic potential for a range of skin conditions.

### 3.9 Skin microbiota analysis

Using α-diversity analysis, we first assessed the diversity and abundance of the skin microbiome. An indicator of sequencing saturation is the Observed Species Index, which is a sequencing depth index. The curves of the administration group, model group, and blank control group finally leveled off, as seen in Supplementary Figure S3A, suggesting that the sequencing volume's present saturation was adequate. Bacterial abundance is shown by the Chao1 index; the higher the index, the more OTUs and thus the richer the community. The model group has the smallest community richness, while the blank control group has the largest, as illustrated in Supplementary Figure S3B. Additionally, there is a notable difference between the model group and the control group, as well as between the model group and the blank group. Bacterial diversity indexes are the Shannon and Simpson indices. These two indexes allow us to assess the diversity of the community. The community diversity was lowest in the model group and highest in the blank group, as seen in Supplementary Figures S3C, S3D. Additionally, there was a significant difference between the model group and the control group as well as between the model group and the blank group. We next used β-diversity analysis to compare the samples' microbial composition (Supplementary Figure S3E). The results showed that there were differences in the microbial composition between different samples.

**Figure 5:**
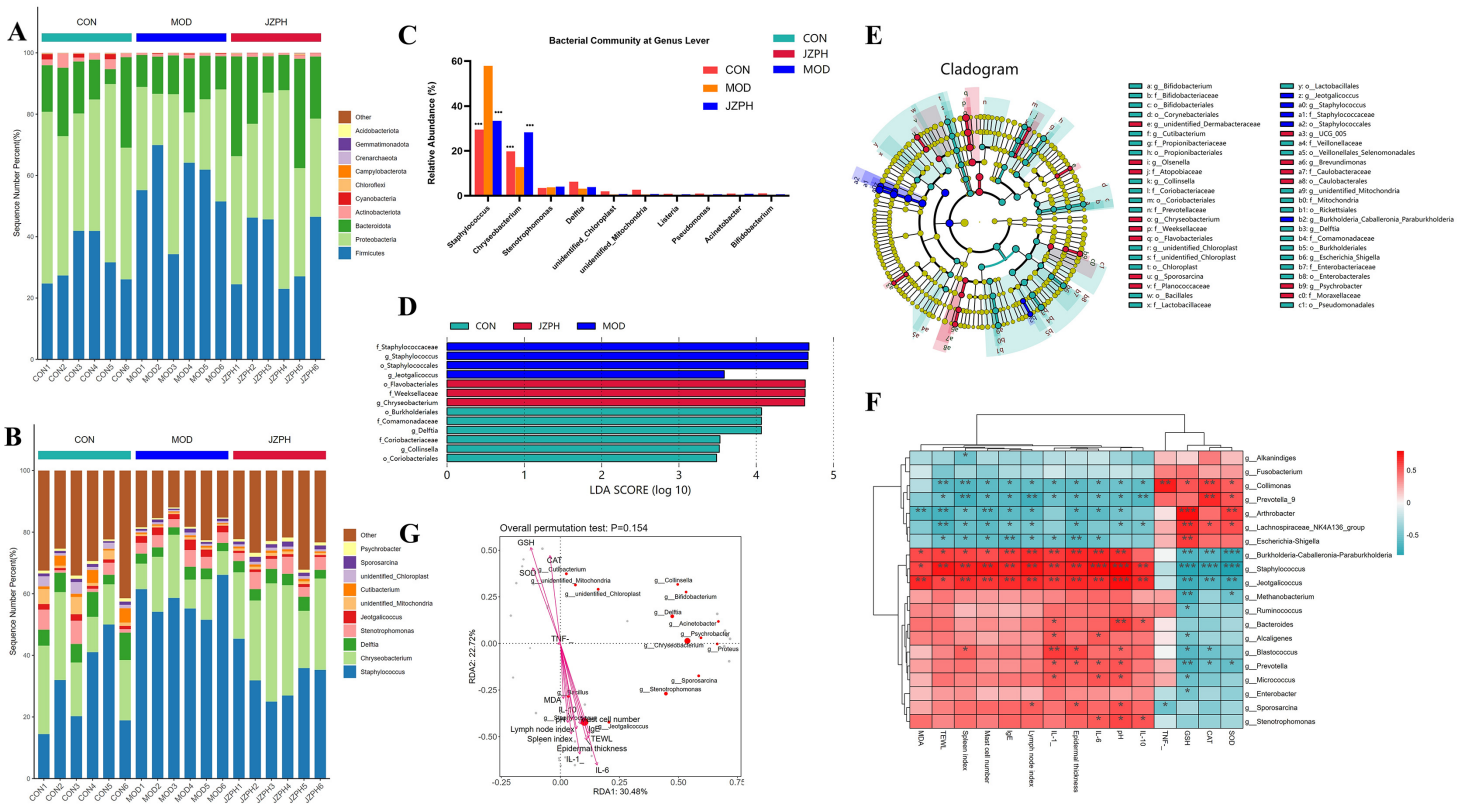
The relative abundances of the phylum-level skin microbiota **(A)**, the relative abundances of the genus-level skin microbiota **(B)** and **(C)**, LEfSe barplot analysis **(D)**, LEfSe cladogram analysis **(E)**, Spearman heatmap analysis between skin flora and index **(F)**, pecies CCA/RDA ordination chart between skin flora and index **(G)** Note: ***represents for vs. MOD group (*p* < 0.001), ###represents for vs. CON group (*p* < 0.001), respectively (*n* = 6).

At the phylum level, the microbiota of all groups, whether it be the blank group vs. the model group or the administration group, consists of the *Firmicutes, Proteobacteria, Bacteroidetes, Actinobacteria, Cyanobacteria, Chloroflexi, Capmpylobacterota, Crenarchaeota, Gemmatimonadota*, and *Acidobacteriota* and so on (Figure 5A). Among them, the *Firmicutes, Proteobacteria, Bacteroidetes*, and *Actinobacteria* are the most abundant phylum. The *Proteobacteria* dominate in the CON and JZPH groups, while the *Firmicutes* dominate in the MOD group. This indicates that there are differences in the phylum-level structure of the microbiota among the different samples. At the genus level, the most abundant genera are *Staphylococcus, Flavobacterium, Delftia*, and *Stenotrophomonas* (Figures 5B, C). In all samples, *Staphylococcus* is the predominant genus. However, *Staphylococcus spp*. accounted for different proportions of the genera contained in each sample, with a significantly higher proportion in the MOD group than in the CON and JZPH groups. This implies that patients with atopic dermatitis are at the highest risk of being infected by bacteria of the genus *Staphylococcus*, and that JZPH is resistant to staphylococcal infections.

LEfSe was performed for characterizing the microbial communities exhibiting significant differences in abundance between the CON group, MOD group, and JZPH group (Figures 5D, E). The translation results show that in the CON group, the relative abundance of *o_Burkholderiales, f_Comamonadaceae, g_Delftia, f_Coriobacteriaceae, g_Collinsella*, and *o_Coriobacteriales* was significantly higher. In the JZPH group, the relative abundance of *o_Flavobacteriales, f_Weeksellaceae*, and *g_Chryseobacterium* was significantly higher. Additionally, in the MOD group, the relative abundance of *f_Staphylococcaceae, g_Staphylococcus, o_Staphylococcales*, and *g_Jeotgalicoccus* increased significantly.

### 3.10 Relationship between skin microbiota and biochemical markers

The relationship between certain Gram-positive bacteria and biochemical indicators was investigated using Spearman heat map analysis and typical redundancy analysis (RDA). As shown in Figure 5F, *Collimonas* is positively correlated with CAT and TNF, and negatively correlated with TEWL and Spleen index. *Prevotella_9* is positively correlated with CAT and negatively correlated with IL-10, Lymph node index, and Spleen index. Arthrobacter is positively correlated with SOD and GSH and negatively correlated with Mast cell number, TWEL, and MDA. *Lachnospiraceae_NK4A136_group* is positively correlated with GSH and negatively correlated with TWEL. *Escherichia-Shigella* is positively correlated with GSH and negatively correlated with Epidermal thickness and IgE. *Burkholderia-Caballeronia-Paraburkholderia* is negatively correlated with CAT, GSH, and SOD and positively correlated with pH, IL-6, IL-1, Lymph node index, Epidermal thickness, and IgE. *Staphylococcus* is negatively correlated with SOD, CAT, and GSH and positively correlated with IL-10, pH, IL-6, IL-1, Lymph node index, Epidermal thickness, IgE, Mast cell number, TWEL, and Spleen index. Moreover, the negative correlation between *Staphylococcus* and SOD, CAT, GSH and the positive correlation with pH and IgE are more significant. This proves that atopic dermatitis (AD) patients indeed have a higher risk of *Staphylococcus aureus* infection. In addition, *Jeotgalicoccus* was found to have a significant negative correlation with SOD, CAT, GSH, and a significant positive correlation with IL-10, pH, IL-6, IL-1, Lymph node index, Epidermal thickness, IgE, Mast cell number, MDA, and Spleen index. The RDA also verifies the above analysis results (Figure 5G). RAD uses the angle, length, and vertical distance of the arrows between the axes of the microbial and parametric variables to illustrate the degree of association between the biochemical markers and the chemicals. The fourth quadrant contains IL-10, pH, IL-6, IL-1, Lymph node index, Epidermal thickness, IgE, Mast cell number, MDA, TWEL, Spleen index, and *Staphylococcus, Jeotgalicoccus*, etc., while the first quadrant contains SOD, CAT, GSH, etc., all pointing to the consistency of the correlation between RDA and Spearman analysis. In other words, the skin of patients with atopic dermatitis (AD) is characterized by dryness and occasional wounds, which provide favorable conditions for *Staphylococcus aureus* to penetrate deeper into the skin layer, triggering an immune response and worsening the condition. However, the focus of this study was on the changes in the skin microbiota of AD patients because of its significant impact on AD symptoms. Future studies will explore the specific role of JZPH against *S. aureus*.

## 4 Discussion

The traditional Chinese medicinal formula JZP comprises *L. erythrorhizon, S. divaricata, A. dahurica, A. sinensis, R. glutinosa, L. japonica, C. myrrha*, and *B. carterii*. JZP, a well-established remedy, has been historically utilized in China for the treatment of various skin disorders with notable efficacy. Despite its documented effectiveness, the underlying mechanism of action of JZP remains largely unexplored. Existing investigations have predominantly delved into the individual components of the formula. For instance, *L. erythrorhizon* has demonstrated a reduction in scratching frequency, ear and epidermal thickness, and inflammatory cell infiltration in atopic dermatitis mice. *L. erythrorhizon* has been shown to modulate the IgG1/IgG2a ratio, decrease serum IgE and histamine levels, restore Th1/Th2 immune balance, and lower serum levels of IL-1β, IL-4, IL-6, and TNF-α (Oh et al., 2021). *S. divaricata*, known for its analgesic properties in traditional Chinese medicine, exhibits significant alleviation of skin inflammation symptoms, restoration of skin barrier function, and reduction in IgE levels and pro-inflammatory cytokines such as TSLP, IL-33, and IL-4. Moreover, *S. divaricata* reduces TRPV1 expression in F4/80+/TRPV1+ cells, leading to a marked decrease in itching (Li et al., 2024). *A. dahurica*, recognized in traditional Chinese medicine for its diverse therapeutic effects, including disease elimination, dampness removal, pus discharge, muscle growth promotion, blood circulation activation, and pain relief, has been found to reduce spleen weight, skin thickness, CD^4+^ cell count, inflammation, and mast cell infiltration in mouse skin. Furthermore, *A. dahurica* decreases IgE, IL-6, IL-10, and IL-12 levels in mouse serum (Ku et al., 2017). *A. sinensis*, a crucial medicinal herb, has been extensively studied across various domains, with specific relevance to atopic dermatitis. Administration of *A. sinensis* extract results in a significant reduction in serum IgE levels, mast cell count, and skin thickness. Additionally, expression levels of NF-κB, P-MAPKs, and cytokines (IL-4, IL-6, TNF-α, and IFN-γ) are diminished in the dorsal skin (Lee et al., 2016). *L. japonica* also exhibits efficacy in atopic dermatitis treatment, as evidenced by increased TSLP levels and reduced skin hyperplasia and pathological defects in mice. Activation of NLRP3 inflammasomes, release of IL-17, and downregulation of p-c-Fos and p-p65 protein expression in cutaneous tissues are observed following extract administration (Bai et al., 2023). The synergistic utilization of these individual herbs markedly improves the therapeutic effectiveness of the formulation for managing atopic dermatitis.

Network analysis predicted that the mechanism of action for treating atopic dermatitis involves the activation of the IL-17 pathway, which has been supported by both computational simulations and existing literature. IL-17, a key pro-inflammatory cytokine, is predominantly produced by CD4^+^ helper T cells (Th17 cells) and innate lymphocyte subsets. IL-17A is implicated in the pathogenesis of diverse inflammatory skin conditions such as AD, hidradenitis suppurativa, alopecia areata, erythrasma, pemphigus, and systemic sclerosis. Additionally, IL-23 plays a vital role in inducing IL-17 production by activating Th17 cells. The IL-23/IL-17 axis represents a critical pathway for targeted therapy in skin inflammatory disorders. Ongoing clinical trials support the efficacy of targeting IL-23 and IL-17 in managing patients with AD, hidradenitis suppurativa, erythrasma, pemphigus, and systemic sclerosis (Liu et al., 2020).

Multiple studies have validated the efficacy of traditional Chinese medicine in treating AD through the activation of the IL-17 pathway. JZP was made up *L. erythrorhizon, S. divaricata, A. dahurica, A. sinensis, R. glutinosa, L. japonica, C. myrrha, B. carterii*. Historically, key constituents of those herbs have been pivotal in AD treatment, concurrently eliciting IL-17 activation to facilitate therapeutic efficacy. For instance, shikonin, the active component of *L. erythrorhizon*, was found to inhibit IL-17 production by targeting the JAK/STAT3 pathway in an inflammation model (Lan et al., 2020). *L. japonica* was observed to modulate gut microbiota, increase beneficial flora, and consequently reduce inflammatory factors, eosinophils, goblet cells, NLRP3 inflammasomes, and the differentiation of splenic Th17 cells (Bai et al., 2022). The polyphenolic compounds, particularly flavonoids present in *C. myrrha*, exhibit anti-inflammatory properties by decreasing ROS and NO production, preserving macrophage viability, and suppressing COX-2, iNOS, TNF-α, IL-1β, IL-6, and IL-17 (Da Silva et al., 2024). Experimental validation has further demonstrated that the compound prescription can ameliorate pharmacodynamic symptoms in a mouse model of atopic dermatitis through the activation of the IL-17 pathway. Numerous studies have explored the role of the IL-17 signaling pathway in treating AD using traditional Chinese medicine formulations. This pathway is a well-established component of inflammation. For instance, the Fangji Dihuang formulation (FJDHF) has demonstrated efficacy in ameliorating AD-like skin lesions in murine models. Through network pharmacology analysis, the key pathway identified in FJDHF is the IL-17 signaling pathway, which interacts with various cytokines. Analysis using single-cell RNA sequencing suggests that FJDHF may mitigate AD by modulating dendritic cells. Experimental findings from flow cytometry and RT-PCR indicate that FJDHF can attenuate the impact of IL-4 and IFN-γ, as well as suppress the expression of IL-17 in AD specimens (Zhao et al., 2023).

The focus of this study is a complex traditional Chinese medicine preparation, presenting challenges in understanding its pharmacological mechanism due to its intricate composition. Isolating the main components or groups from the herbs results in reduced pharmacological effects compared to the original compound, suggesting potential synergistic interactions among molecules contributing to the efficacy. The self-assembly of traditional Chinese medicine formulations exemplifies a common occurrence characterized by the spontaneous organization into structured entities (e.g., nanoparticles, nanofibers, micelles, and vesicles) facilitated by non-covalent interactions, such as hydrogen bonding, electrostatic forces, van der Waals forces, and π-π stacking. Research indicates that self-assembly processes are pervasive throughout various stages of TCM, encompassing the sourcing of raw herbs, their preparation, decoction, formulation, storage, and eventual administration. Chinese herbal remedies comprise a diverse array of bioactive compounds, including alkaloids, flavonoids, polysaccharides, volatile oils, glycosides, and tannins, each possessing distinct pharmacological properties. The self-assembly dynamics may manifest intra-componentally or inter-componentally, thereby influencing the overall therapeutic outcomes.

AD is closely associated with alterations in the skin microbiota, particularly an overabundance of *Staphylococcus aureus*, which exacerbates the condition by compromising the skin barrier and weakening the immune response. Studies have shown that the efficacy of treatment following microbiota transplantation is inversely related to *S. aureus* levels, confirming its pathogenic role (Edslev et al., 2020). Further investigations have linked the reduction of *S. aureus* strains to neutrophil activity and the IL-17 pathway (Simpson et al., 2023). *S. aureus* not only diminishes neutrophil levels but also prompts keratinocytes to produce α-toxin, triggering cellular inflammatory responses that worsen skin inflammation (Geoghegan et al., 2018). Given the significant role of *S. aureus* in atopic dermatitis, clinical studies often assess skin microbiota composition, particularly *S. aureus* abundance, as a severity indicator (Byrd et al., 2017). While prior research has primarily focused on microbiota analysis and pharmacodynamics, a recent study integrated network analysis, skin microbiota profiling, and pathway validation to elucidate the therapeutic impact of JZP. This study identified a relationship between reshaping the skin microbiota and activating the IL-17 pathway. Nevertheless, further in-depth investigations are needed to fully understand the underlying mechanisms.

The relationship between skin microbes and the IL-17 pathway is significant. The abundance of human skin commensal, symbiont, and mutualistic microbes equals or exceeds that of eukaryotic cells. Immune adaptation to eukaryotic cell antigens occurs through central and peripheral tolerance mechanisms to prevent autoimmunity. Similarly, the immune system gradually responds to microbiota antigens, a concept termed adaptation to the “extended self” (Iwakura et al., 2011). Maintenance of self-extension tolerance relies on a delicate equilibrium between regulatory T cells (Tregs) and helper T cells (Ths). Tregs mitigate excessive immune reactions, while Th cells swiftly act when commensal species proliferate or new species emerge in the microbiome. Antigens and metabolites produced within specific microbiome conditions modulate the expansion or contraction of Tregs and effector Th cells. For instance, the interplay between the microbiota and the immune system prompts mouse Th cells to generate IL-17. Th17 cells, producing IL-17 and IL-22, safeguard intestinal mucosal integrity, promote local plasma cell maturation for IgA production, and deter bacterial colonization. Furthermore, fibroblasts, endothelial cells, chondrocytes, and adipocytes respond to IL-17A by releasing antimicrobial proteins, peptides, pro-inflammatory cytokines, and chemokines crucial for acute-phase responses and tissue remodeling (Gaffen et al., 2014). Consequently, organisms deficient in IL-17A exhibit heightened susceptibility to fungal and bacterial skin and mucous membrane infections (Veldhoen, 2017).

Therefore, investigating the JPZ can advance the contemporary utilization of traditional preparation. Subsequent computational network analysis utilized existing databases, revealing polyphenols as the primary components. While literature suggests that polyphenols may yield false-positive results, some studies highlight their importance for human health. Consequently, this study hypothesizes that polyphenols serve as the active components through which the compound exerts its therapeutic effects, warranting further experimental validation. The computer-based speculation on the mechanism of action represents an initial step, with outcomes being meticulously chosen. This conclusion is acknowledged. Utilizing network pharmacology and pharmacodynamics analysis, this study investigated the potential mechanism of action of the Modified JPZ for treating AD. However, further research is imperative. This study comprises a network pharmacological analysis and *in vivo* animal study of JZP. Further investigations are warranted to explore the correlation between the formula ratio and efficacy of this prescription, along with an assessment of its clinical utility, in order to establish a robust groundwork for its contemporary use.

## 5 Conclusions

Atopic dermatitis, a chronic inflammatory skin disorder affecting millions worldwide, necessitates effective therapeutic interventions to improve quality of life, prevent complications, and minimize disease recurrence. Our study demonstrates that JZP exhibits potent therapeutic effects against atopic dermatitis through dual mechanisms involving both immunomodulation and microbial regulation. Systematic experimental validation has confirmed JZP's therapeutic efficacy and mechanistic basis. These findings position topical JZP as a promising alternative or adjunctive therapy for AD management, while simultaneously advancing our understanding of AD pathophysiology to inform future drug development.

## Data Availability

The original contributions presented in the study are included in the article/Supplementary material, further inquiries can be directed to the corresponding authors.
