# Supplementary material for: Mechanistic study of Jiawei Zicao Plaster in atopic dermatitis via IL-17 signaling pathway and skin microbiome modulation
Source: Front Microbiol. 2025 Sep 24;16:1668089. doi: 10.3389/fmicb.2025.1668089 (PMC12504314; doi:10.3389/fmicb.2025.1668089)
Supplement: Supplementary file 1 [file Supplementary_file_1.docx]

**Mechanistic Study of Jiawei Zicao Plaster in Atopic Dermatitis *via* IL-17 Signaling Pathway and Skin Microbiome Modulation**

Andong Wang ^1,#^, Tingting Chen ^1,#^, Hongtao Zhang ^1^, Yuru Yang ^1^, Xiaotian Cheng ^1,2^, Di Chen ^3,*^, Bai Ling ^1,2,*^

^1^ School of Pharmacy, Nantong University, Nantong, Jiangsu 226001, P. R. China;

^2^ Department of Pharmacy, The Yancheng Clinical College of Xuzhou Medical University & The First people’s Hospital of Yancheng, Yancheng, Jiangsu 224001, P. R. China;

^3^ Scientific Research Department, Ningbo Municipal Hospital of Traditional Chinese Medicine & Affiliated Hospital of Zhejiang Chinese Medical University, Ningbo, Zhejiang 315010, P. R. China;

* Correspondence: Di Chen: [zjnbcd@163.com](mailto:zjnbcd@163.com) ; Bai Ling: [lingbai@163.com](mailto:lingbai@163.com)

# These authors contributed equally to this work and shared first authorship.


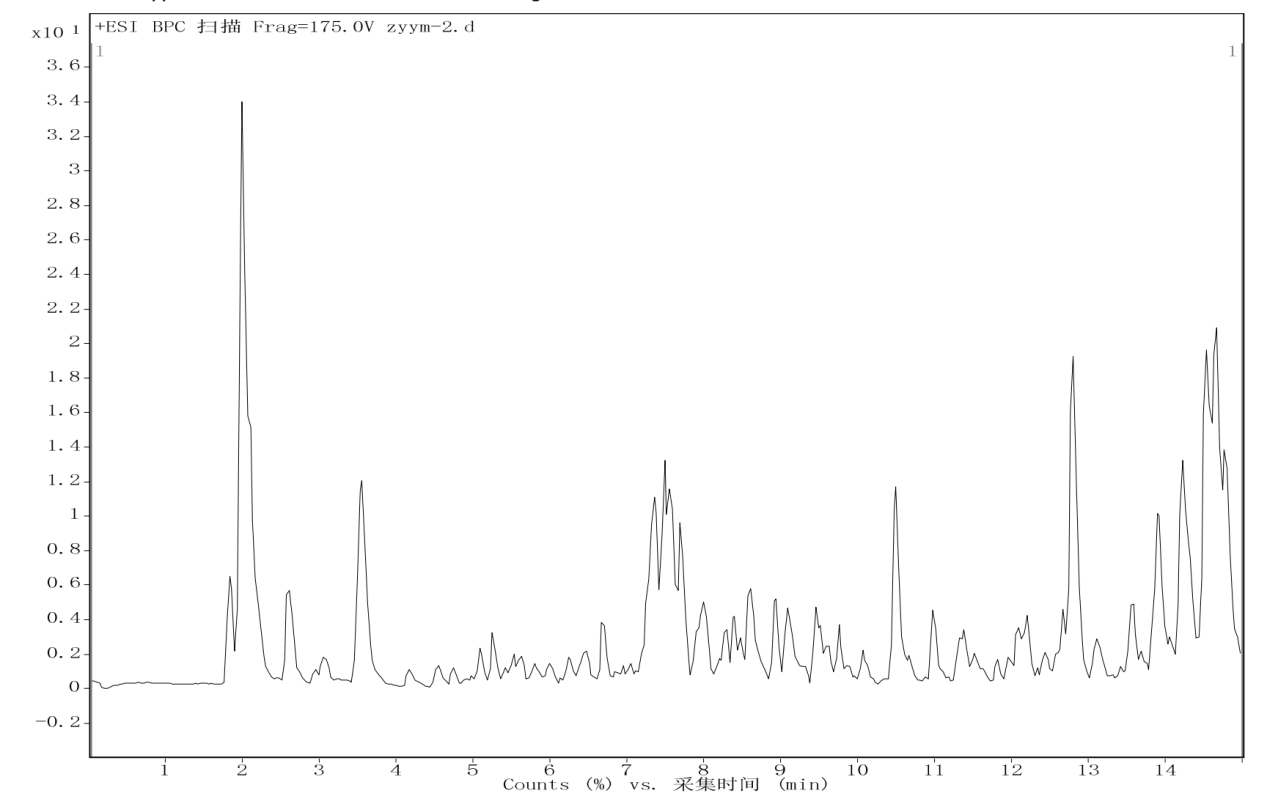


Figure S1 Chromatograms in UPLC-Q-TOF/MS


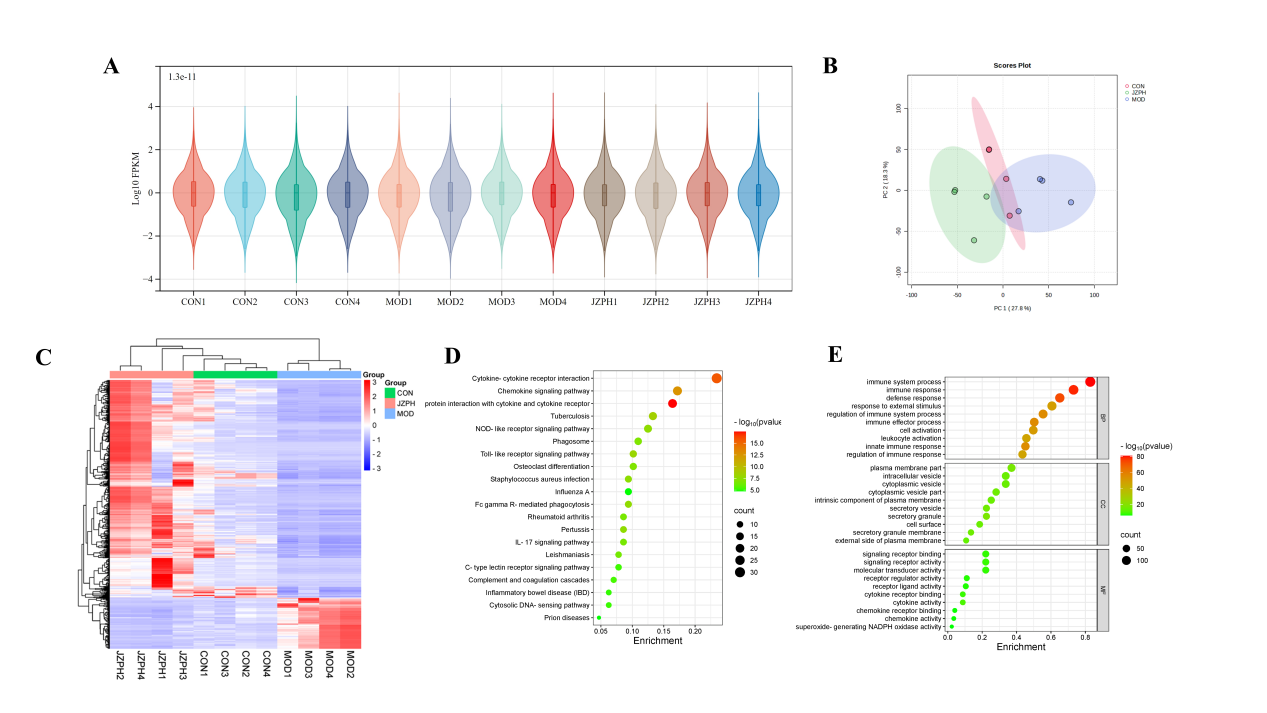


Figure S2 FPKM analysis (A), PCA analysis (B), Analysis of Clustering heatmap (C), KEGG functional pathway analysis (D), GO pathway enrichment analysis (E). (n=3)


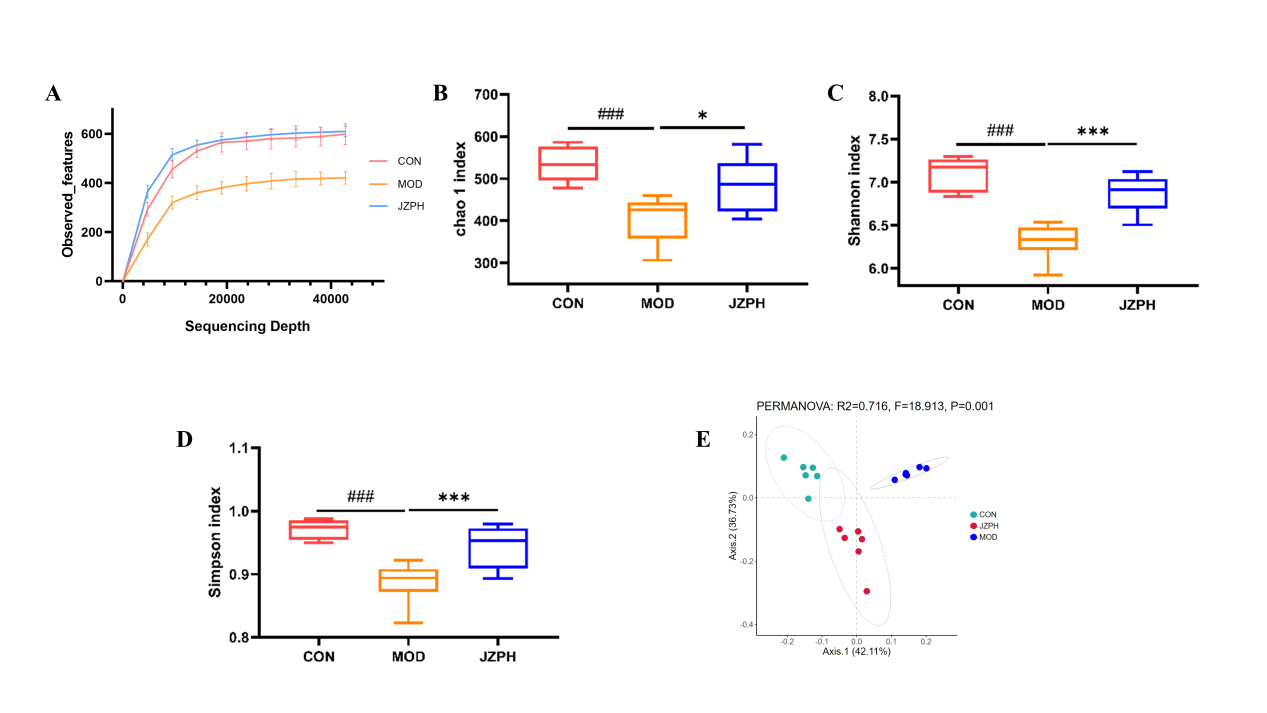


Figure S3 Observed_features (A), Chao1 indices (B), Shannon indices (C), Simpson indices (D), PCA analysis (E) Note: * represents for vs MOD group (p < 0.05), ** represents for vs MOD group (p < 0.01), *** represents for vs MOD group (p < 0.001), ### represents for vs CON group (p < 0.001), respectively. (n=6)

Table S1 Summary table of relevant data for typical compounds in plants

| Drug Name | Rt  (min) | Compound Name | Molecular Formula | CAS numble | Accurated Mass  (m/z) | Theoretical Mass  (m/z) | Error  (ppm) | Ion Species |
| --- | --- | --- | --- | --- | --- | --- | --- | --- |
| *Lithospermum erythrorhizon* | 12.81 | shikonin | C_16_H_16_O_5_ | 517-89-5 | 288.0998 | 288.0995 | 1 | [M]+ |
|  | 7.56 | alkannin beta,beta-dimethylacrylate | C_21_H_22_O_6_ | 34539-65-6 | 393.1314 | 393.1309 | 1.3 | [M+Na]+ |
| *Saposhnikovia divaricata* | 10.38 | prim-O-glucosylcimifugin | C_22_H_28_O_11_ | 80681-45-4 | 469.1701 | 469.1704 | -0.6 | [M+H]+ |
|  | 3.61 | 4'-O-beta-Glucopyranosyl-5-O-Methylvisamminol | C_22_H_28_O_10_ | 84272-85-5 | 475.1586 | 475.1575 | 2.3 | [M+Na]+ |
| *Angelica dahurica* | 6.83 | imperatorin | C_16_H_14_O_4_ | 482-44-0 | 270.0886 | 270.0892 | -2.2 | [M]+ |
| *Angelica sinensis* | 2.07 | ferulate | C_10_H_10_O_4_ | 1135-24-6 | 195.0672 | 195.0672 | 0 | [M]+ |
| *Rehmannia glutinosa* | 13.92 | catalpol | C_15_H_22_O_10_ | 2415-24-9 | 385.1114 | 385.1105 | 2.3 | [M+Na]+ |
|  | 2.65 | martynoside | C_31_H_40_O_15_ | 67884-12-2 | 675.2253 | 675.2259 | -0.9 | [M+Na]+ |
| *Lonicera japonica* | 2.11 | Chlorogenic acid | C_16_H_18_O_9_ | 327-97-9 | 337.0907 | 337.0923 | -4.7 | [M+H]+ |
|  | 1.95 | Loganin | C_17_H_26_O_10_ | 18524-94-2 | 413.1402 | 413.1419 | -4.1 | [M+Na]+ |

Table S2 Topological statistics of network analysis

| name | BetweennessCentrality | ClosenessCentrality | ClusteringCoefficient | Degree | Eccentricity | NumberOfUndirectedEdges |
| --- | --- | --- | --- | --- | --- | --- |
| KCNH2 | 0.000452 | 0.500000 | 0.466667 | 6 | 3 | 6 |
| GSTA2 | 0.000045 | 0.450758 | 0.857143 | 8 | 4 | 8 |
| GSTM1 | 0.000878 | 0.510730 | 0.590476 | 15 | 4 | 15 |
| SLPI | 0.000000 | 0.493776 | 1.000000 | 10 | 4 | 10 |
| CLDN4 | 0.000053 | 0.495833 | 0.833333 | 9 | 4 | 9 |
| CHRM4 | 0.000000 | 0.335211 | 0.000000 | 1 | 4 | 1 |
| HTR2A | 0.004522 | 0.464844 | 0.254545 | 11 | 3 | 11 |
| PTGER3 | 0.003046 | 0.512931 | 0.545455 | 11 | 3 | 11 |
| CHRNA7 | 0.000000 | 0.309896 | 0.000000 | 1 | 4 | 1 |
| CHRM3 | 0.000000 | 0.340974 | 1.000000 | 2 | 4 | 2 |
| CHRM1 | 0.021033 | 0.447368 | 0.190476 | 7 | 3 | 7 |
| THBD | 0.000190 | 0.538462 | 0.883333 | 25 | 4 | 25 |
| PON1 | 0.000289 | 0.510730 | 0.783333 | 16 | 4 | 16 |
| CA2 | 0.000000 | 0.445693 | 1.000000 | 2 | 4 | 2 |
| PRKCA | 0.002881 | 0.607143 | 0.708213 | 46 | 3 | 46 |
| PRKCD | 0.000554 | 0.548387 | 0.798030 | 29 | 4 | 29 |
| LTA4H | 0.000032 | 0.489712 | 0.733333 | 6 | 4 | 6 |
| MYC | 0.013162 | 0.712575 | 0.668641 | 74 | 3 | 74 |
| TRPV1 | 0.001928 | 0.528889 | 0.731579 | 20 | 3 | 20 |
| PRKCB | 0.000333 | 0.524229 | 0.742690 | 19 | 3 | 19 |
| HIF1A | 0.005852 | 0.725610 | 0.677033 | 77 | 3 | 77 |
| IKBKB | 0.001785 | 0.626316 | 0.803167 | 52 | 3 | 52 |
| CXCL2 | 0.000969 | 0.569378 | 0.844920 | 34 | 3 | 34 |
| RXRA | 0.001776 | 0.533632 | 0.628571 | 21 | 4 | 21 |
| PIK3CG | 0.011193 | 0.569378 | 0.698851 | 30 | 3 | 30 |
| TOP2A | 0.000110 | 0.512931 | 0.908497 | 18 | 4 | 18 |
| RAF1 | 0.000644 | 0.561321 | 0.812500 | 32 | 3 | 32 |
| IGF2 | 0.004898 | 0.580488 | 0.769559 | 38 | 3 | 38 |
| PPARA | 0.004402 | 0.643243 | 0.696753 | 56 | 3 | 56 |
| CDKN1A | 0.001291 | 0.626316 | 0.825038 | 52 | 3 | 52 |
| BCL2 | 0.010861 | 0.753165 | 0.612695 | 83 | 3 | 83 |
| NFE2L2 | 0.013339 | 0.661111 | 0.678477 | 62 | 3 | 62 |
| MAPK8 | 0.000878 | 0.629630 | 0.866516 | 52 | 3 | 52 |
| SPP1 | 0.002433 | 0.610256 | 0.806973 | 49 | 4 | 49 |
| PPP3CA | 0.000047 | 0.487705 | 0.863636 | 12 | 4 | 12 |
| IGFBP3 | 0.000954 | 0.577670 | 0.823613 | 38 | 4 | 38 |
| XDH | 0.000892 | 0.569378 | 0.830660 | 34 | 4 | 34 |
| BCL2L1 | 0.002156 | 0.657459 | 0.788701 | 60 | 3 | 60 |
| ALOX5 | 0.000698 | 0.536036 | 0.844156 | 22 | 3 | 22 |
| PLAU | 0.000818 | 0.592040 | 0.859358 | 43 | 4 | 43 |
| PTEN | 0.003885 | 0.676136 | 0.745673 | 65 | 3 | 65 |
| JUN | 0.010625 | 0.753165 | 0.615927 | 83 | 3 | 83 |
| CD40LG | 0.000357 | 0.566667 | 0.899160 | 35 | 3 | 35 |
| CHUK | 0.001061 | 0.610256 | 0.848289 | 47 | 3 | 47 |
| CASP7 | 0.000014 | 0.545872 | 0.989418 | 28 | 4 | 28 |
| MCL1 | 0.000625 | 0.601010 | 0.875264 | 44 | 3 | 44 |
| PARP1 | 0.001674 | 0.626316 | 0.803922 | 52 | 3 | 52 |
| STAT1 | 0.003268 | 0.672316 | 0.754464 | 64 | 3 | 64 |
| DPP4 | 0.000826 | 0.561321 | 0.794355 | 32 | 4 | 32 |
| CASP8 | 0.002309 | 0.653846 | 0.779661 | 60 | 3 | 60 |
| FN1 | 0.009190 | 0.695906 | 0.657971 | 70 | 3 | 70 |
| SELE | 0.000576 | 0.586207 | 0.876923 | 40 | 3 | 40 |
| NOS2 | 0.003344 | 0.616580 | 0.753546 | 48 | 3 | 48 |
| GSK3B | 0.005307 | 0.672316 | 0.694444 | 64 | 3 | 64 |
| MMP1 | 0.001084 | 0.595000 | 0.854123 | 44 | 4 | 44 |
| CASP3 | 0.012637 | 0.772727 | 0.585405 | 87 | 3 | 87 |
| PPARD | 0.001220 | 0.569378 | 0.796791 | 34 | 3 | 34 |
| HAS2 | 0.000011 | 0.500000 | 0.963636 | 11 | 4 | 11 |
| CXCL11 | 0.000239 | 0.545872 | 0.898462 | 26 | 4 | 26 |
| CXCL10 | 0.001939 | 0.616580 | 0.813776 | 49 | 3 | 49 |
| MMP3 | 0.001010 | 0.595000 | 0.864646 | 45 | 4 | 45 |
| VCAM1 | 0.003901 | 0.668539 | 0.721454 | 63 | 3 | 63 |
| NCF1 | 0.000768 | 0.563981 | 0.791398 | 31 | 3 | 31 |
| GJA1 | 0.001781 | 0.548387 | 0.822751 | 28 | 4 | 28 |
| SOD1 | 0.001388 | 0.566667 | 0.807487 | 34 | 4 | 34 |
| ERBB3 | 0.000282 | 0.548387 | 0.855385 | 26 | 3 | 26 |
| IL1A | 0.006849 | 0.700000 | 0.681288 | 71 | 3 | 71 |
| HSPB1 | 0.000534 | 0.577670 | 0.862020 | 38 | 4 | 38 |
| IRF1 | 0.000424 | 0.589109 | 0.896154 | 40 | 3 | 40 |
| CTSD | 0.000121 | 0.540909 | 0.913043 | 24 | 4 | 24 |
| MAPK14 | 0.002439 | 0.646739 | 0.791353 | 57 | 3 | 57 |
| CCND1 | 0.004524 | 0.687861 | 0.707638 | 68 | 3 | 68 |
| IL2 | 0.002750 | 0.664804 | 0.767319 | 62 | 3 | 62 |
| CCL2 | 0.007548 | 0.712575 | 0.651611 | 74 | 3 | 74 |
| MPO | 0.002630 | 0.604061 | 0.761353 | 46 | 4 | 46 |
| SERPINE1 | 0.004775 | 0.639785 | 0.716792 | 57 | 4 | 57 |
| TGFB1 | 0.008017 | 0.725610 | 0.655844 | 77 | 3 | 77 |
| MMP2 | 0.002896 | 0.668539 | 0.755248 | 63 | 3 | 63 |
| HMOX1 | 0.010173 | 0.672316 | 0.705769 | 65 | 3 | 65 |
| MAPK1 | 0.003966 | 0.643243 | 0.715584 | 56 | 3 | 56 |
| CYP3A4 | 0.011316 | 0.569378 | 0.452652 | 33 | 3 | 33 |
| PTGS1 | 0.003030 | 0.556075 | 0.653439 | 28 | 3 | 28 |
| NQO1 | 0.003855 | 0.569378 | 0.702317 | 34 | 4 | 34 |
| AKR1C3 | 0.000117 | 0.491736 | 0.678571 | 8 | 4 | 8 |
| HSP90AA1 | 0.022730 | 0.708333 | 0.599696 | 73 | 3 | 73 |
| CYP1A1 | 0.006436 | 0.566667 | 0.590018 | 34 | 4 | 34 |
| ESR1 | 0.021282 | 0.748428 | 0.574526 | 82 | 3 | 82 |
| CYP1B1 | 0.001604 | 0.524229 | 0.617647 | 17 | 4 | 17 |
| CTNNB1 | 0.010016 | 0.716867 | 0.656216 | 75 | 3 | 75 |
| RELA | 0.027886 | 0.691860 | 0.698779 | 67 | 3 | 67 |
| ESR2 | 0.005328 | 0.597990 | 0.752613 | 42 | 3 | 42 |
| NR1I2 | 0.002175 | 0.524229 | 0.594771 | 18 | 4 | 18 |
| NR1I3 | 0.000113 | 0.459459 | 0.750000 | 8 | 4 | 8 |
| AR | 0.003960 | 0.601010 | 0.756368 | 43 | 3 | 43 |
| AHR | 0.000413 | 0.489712 | 0.666667 | 10 | 4 | 10 |
| PRKACA | 0.003409 | 0.613402 | 0.702128 | 47 | 3 | 47 |
| OPRM1 | 0.002018 | 0.533632 | 0.609524 | 21 | 4 | 21 |
| TNF | 0.043793 | 0.843972 | 0.476566 | 100 | 3 | 100 |
| AKT1 | 0.023854 | 0.809524 | 0.517502 | 94 | 3 | 94 |
| CHRM2 | 0.031247 | 0.502110 | 0.327273 | 11 | 3 | 11 |
| CXCL8 | 0.006343 | 0.704142 | 0.677621 | 72 | 3 | 72 |
| IL6 | 0.044644 | 0.850000 | 0.484024 | 99 | 3 | 99 |
| PTGS2 | 0.036468 | 0.788079 | 0.526841 | 90 | 3 | 90 |
| IL10 | 0.017809 | 0.743750 | 0.617981 | 79 | 3 | 79 |
| MMP9 | 0.019676 | 0.762821 | 0.595238 | 85 | 3 | 85 |
| MAOA | 0.002211 | 0.536036 | 0.631579 | 20 | 3 | 20 |
| NOS3 | 0.002729 | 0.619792 | 0.727059 | 51 | 4 | 51 |
| PPARG | 0.009613 | 0.734568 | 0.620578 | 79 | 3 | 79 |
| EGFR | 0.015114 | 0.748428 | 0.581752 | 82 | 3 | 82 |
| ERBB2 | 0.006394 | 0.683908 | 0.674355 | 67 | 3 | 67 |
| TP53 | 0.016176 | 0.772727 | 0.574445 | 87 | 3 | 87 |
| ICAM1 | 0.004018 | 0.683908 | 0.728630 | 67 | 3 | 67 |
| IL1B | 0.041396 | 0.843972 | 0.491689 | 98 | 3 | 98 |
| SLC6A4 | 0.001198 | 0.519651 | 0.696970 | 12 | 3 | 12 |
| NR3C1 | 0.007801 | 0.619792 | 0.676871 | 49 | 3 | 49 |
| IL4 | 0.004043 | 0.672316 | 0.722718 | 64 | 3 | 64 |
| IFNG | 0.006783 | 0.716867 | 0.667748 | 75 | 3 | 75 |
| SLC6A2 | 0.000638 | 0.485714 | 0.428571 | 7 | 3 | 7 |
| NFKBIA | 0.003759 | 0.691860 | 0.731458 | 69 | 3 | 69 |
| ADRB2 | 0.003238 | 0.545872 | 0.699275 | 24 | 3 | 24 |
