# Supplementary material for: Mechanistic study of Jiawei Zicao Plaster in atopic dermatitis via IL-17 signaling pathway and skin microbiome modulation
Source: Front Microbiol. 2025 Sep 24;16:1668089. doi: 10.3389/fmicb.2025.1668089 (PMC12504314; doi:10.3389/fmicb.2025.1668089)
Supplement: Supplementary file 2 [file Data_Sheet_1.pdf]

南通大学实验动物中心标准操作规程

The Standard Operating Procedures for Laboratory Animal Center of NTU

动物实验伦理审查表

The Tab of Animal Experimental Ethical Inspection

编号(No)：

S20240927-004

<table>
<tr><td rowspan="12">申请人填写的相关信息(Information Of applicant)</td><td colspan="6">申请单位(Name of organization)： 南通大学</td></tr>
<tr><td colspan="6">主要申请人姓名(Name of applicant)： 王安东
学历(Education)： 博士研究生
技术职称(Professional title)： 讲师
江苏省培训记录卡编号(No. of training card)： 无

主要成员姓名(Name of applicant)： 成效天
学历(Education)： 硕士研究生
技术职称(Professional title)： 无
江苏省培训记录卡编号(No. of training card)： 220227603

主要成员姓名(Name of applicant)： 杨玉茹
学历(Education)： 硕士研究生
技术职称(Professional title)： 无
江苏省培训记录卡编号(No. of training card)： 220240304</td></tr>
<tr><td colspan="6">项目名称(Title of project)： 中药复方治疗皮炎的机制研究</td></tr>
<tr><td colspan="6">实验目的(Aim of experiment)： 探讨中药复方治疗皮炎的药效物质，以及该中药复方的作用机制</td></tr>
<tr><td rowspan="8">实验动物情况</td><td colspan="5">动物来源(Source of animal)： 南通大学实验动物中心</td></tr>
<tr><td colspan="5">品种品系(Species or strain)： BALB/c</td></tr>
<tr><td>等级(Grade)</td><td>性别(Sex)</td><td>周龄(Week age)</td><td>体重(Body weight)</td><td>数量(Number)</td></tr>
<tr><td>SPF</td><td>雌</td><td>6-8</td><td>20±2</td><td>25</td></tr>
<tr><td colspan="3">申请日期(Application date)：
2024 年 09 月 05 日</td><td colspan="2">批准日期(Approved date)：
2024 年 09 月 27 日</td></tr>
<tr><td colspan="3">进驻日期(Entering date)：
2024 年 10 月 08 日</td><td colspan="2">结束日期(Ending date)：
2024 年 11 月 08 日</td></tr>
</table>

NTULAC

NTULAC

南通大学实验动物中心标准操作规程

The Standard Operating Procedures for Laboratory Animal Center of NTU

实验要点，包括动物分组、饲养条件、处置情况（如前期准备、手术过程和术后护理等）、观测指标、安乐死及尸体处理方式等(Key points: Animal grouping, breeding condition, experimental methods, observational index, euthanasia et. al)：

动物分组**(Animal grouping)**：

对照组	实验组1	实验组2	实验组3	实验组4	实验组5	实验组6
5	5	5;	5	5		

动物处理**(Materials and methods)**：

正常饲喂（饲料Co60辐照灭菌，饲喂高压灭菌水，自由采食和饮水），仅在手术前12h暂时禁水禁食防止手术期间排泄造成感染以及因肠梗阻意外死亡 光周期：12：12h明暗周期 其它条件：每周更换1次高压灭菌玉米芯垫料，每笼5只饲养 手术及护理过程：无 动物出现异常情况下如何处理：若饲养过程中出现突发性濒死现象，采用C02安乐死，白色透明自封袋封存后送交实验动物中心集中进行无害化处理。 给药方式及注意事项：本实验整个过程造模阶段需在第0和第4天分别在小鼠背部裸露皮肤处给予100 µL 高浓度造模药（1% DNCB），给药方式是局部涂抹。之后每周2次，继续局部涂抹100 µL 低浓度造模药（0.2% DNCB），直至造模成功。造模成功后，局部涂抹14天给予治疗药物。治疗药量参照《药理实验方法学》以临床上每日推荐剂量进行换算，在合理区间范围内，出现副作用的概率极低。实验结束，采用C02室息法安乐死。若造模过程中小鼠出现痉挛抽搐、大小便失禁等症状，则该鼠不在进行后续实验，采用CO2室息法安乐死。

观测指标**(Indexes to observation)**：

小鼠行为学改变，H＆E观察病理变化，相关蛋白表达水平，相关mRNA的表达情况等。

安乐死及尸体处理方式 **(Euthanasia)**：

CO2安乐死，白色透明自封袋封存后送交实验动物中心集中进行无害化处理。

主要申请人签名(Signature of Applicant)： 联系电话(Telephone)：

南通大学实验动物中心标准操作规程

The Standard Operating Procedures for Laboratory Animal Center of NTU

<table>
<tr><td>审查
依据
(Inspection criteria)</td><td colspan="4">该项目是否必须用实验动物进行实验，能否用计算机模拟、细胞培养等非生命方法替代动物或用低等动物替代高等动物进行实验(Does laboratory animal must be used in the project Could other methods such as computer simulation, cell cultivation or using the low-grade animal instead of the high-grade animal)？
该项目必须用实验动物进行实验，不能用计算机模拟、细胞培养等非生命方法替代动物或用低等动物替代高等动物进行实验。

表中所填申请人资格和所用动物的品种品系、质量等级、规格是否合适，能否通过改良设计方案或用高质量的动物来减少所用动物的数量(Are the qualification of applicant，species or strain, grade and specifications of animals suitable Could the quantity of animals be reduced by improving the study design or using high quality animals)？
申请人资格和所用动物的品种品系、质量等级、规格合适，不能通过改良设计方案或用高质量的动物来减少所用动物的数量。

能否通过改进实验方法、调整实验观测指标、改良处死动物的方法，来优化实验方案、善待动物(Could the study design and animal treatment be refined by ameliorating experimental method, adjusting observational index, executing animal method)？
实验方法、实验观测指标、处死动物的方法符合动物伦理要求，此实验不能通过改进实验方法、调整实验观测指标、改良处死动物的方法来优化实验方案、善待动物。</td></tr>
<tr><td rowspan="3">审查结果
(是否同意申请人的实验方案)
(Results of inspection)</td><td>课题负责人意见
(Principal Investigator)：</td><td>同意
(Agree) ☐</td><td>不同意
(Disagree) ☐</td><td>签 名
(Signature)</td></tr>
<tr><td>质量保障室主任意见
(Director of QC Department)：</td><td>同意
(Agree) ☐</td><td>不同意
(Disagree) ☐</td><td>签 名
(Signature)</td></tr>
<tr><td>实验动物伦理委员会意见
(Director of IACUC)：</td><td>同意
(Agree) ☐</td><td>不同意
(Disagree) ☐</td><td>签 名
(Signature)</td></tr>
<tr><td colspan="4">备注(Supplement)：</td><td>签章(Stamp)</td></tr>
</table>
