# Supplementary figures and images for: Mechanistic study of Jiawei Zicao Plaster in atopic dermatitis via IL-17 signaling pathway and skin microbiome modulation
Source: Front Microbiol. 2025 Sep 24;16:1668089. doi: 10.3389/fmicb.2025.1668089 (PMC12504314; doi:10.3389/fmicb.2025.1668089)

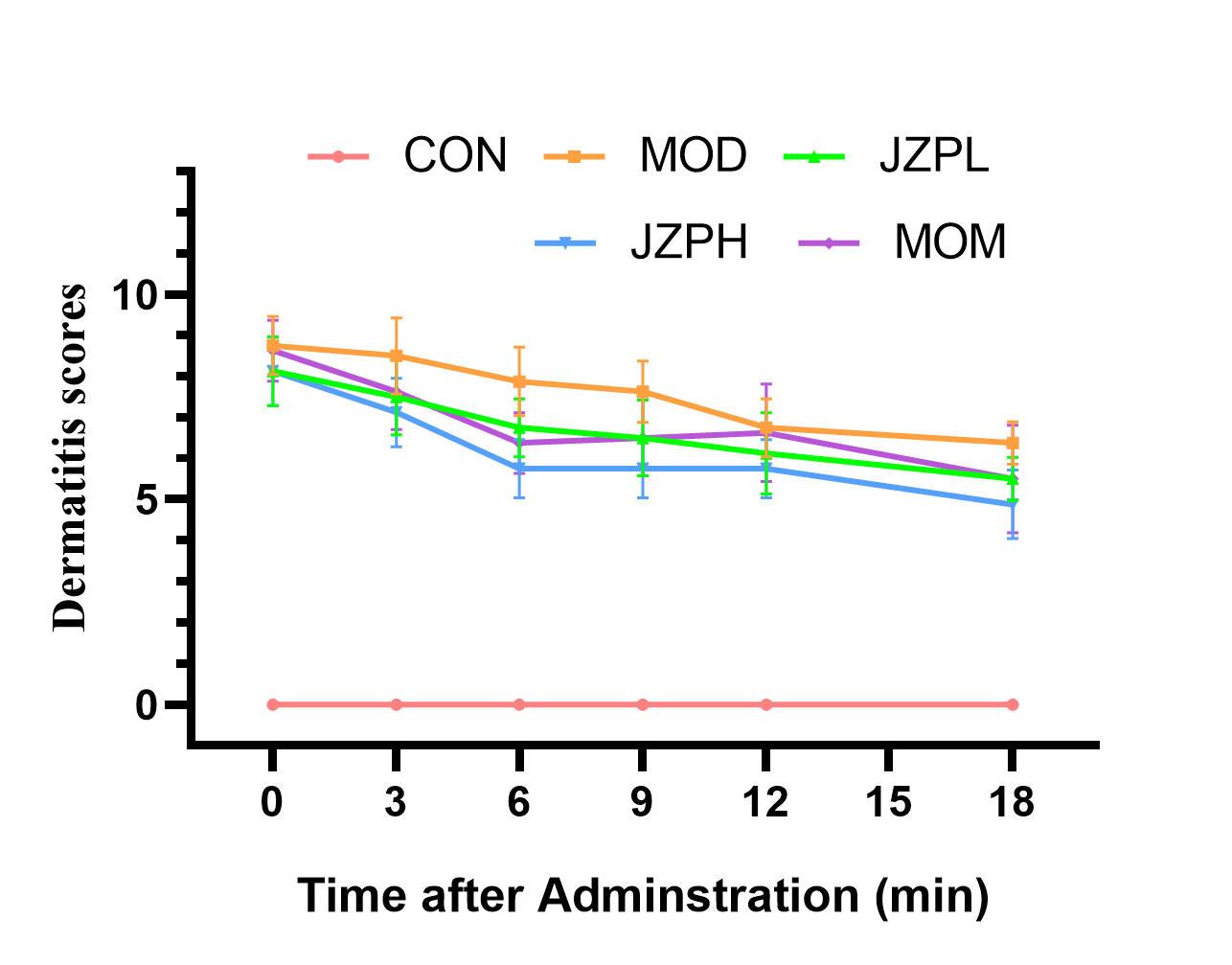

Supplement: Supplementary file 3 [file Data_Sheet_2.zip › Source data/FIGURE 2/Dermatitis scores.png]

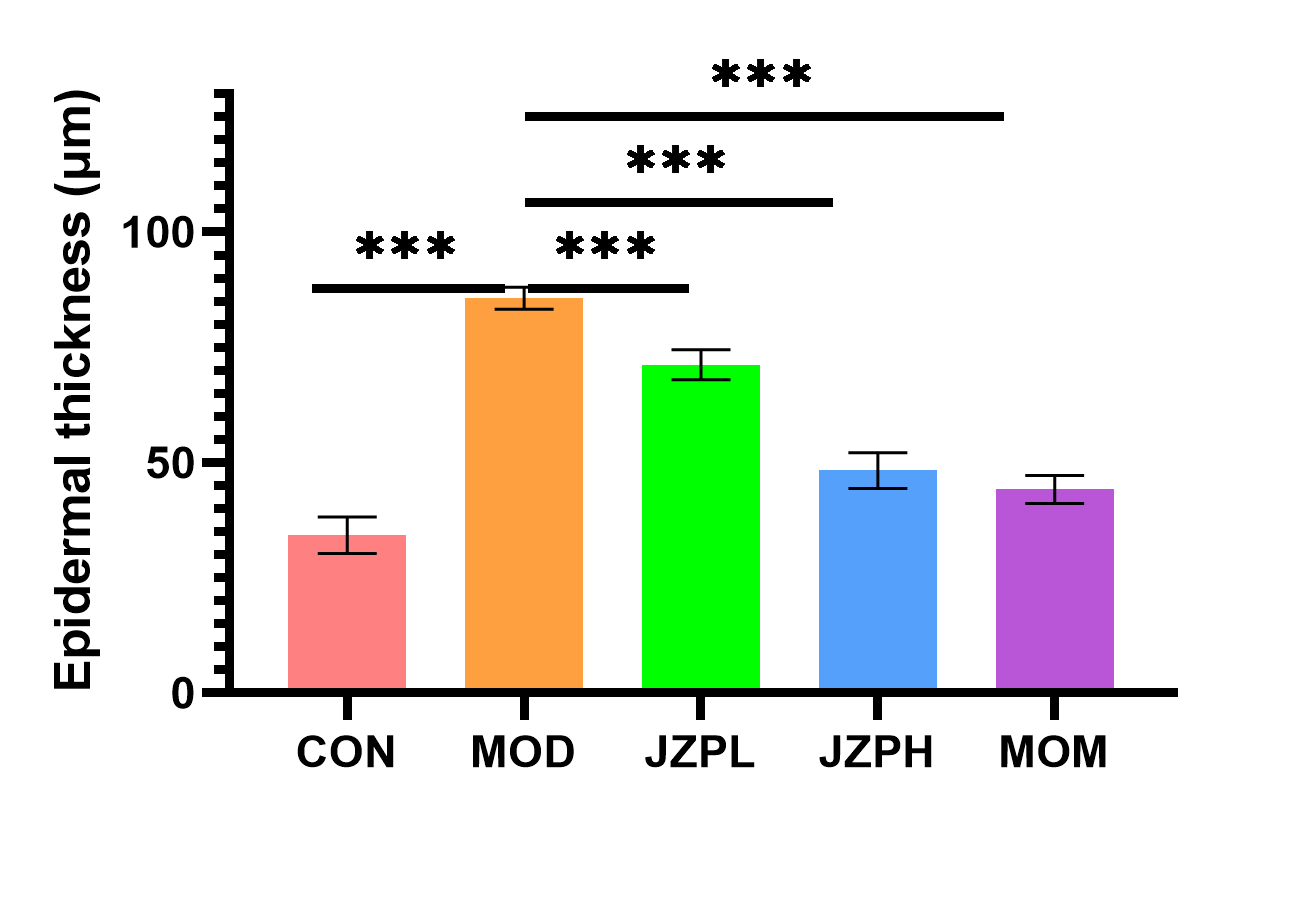

Supplement: Supplementary file 3 [file Data_Sheet_2.zip › Source data/FIGURE 2/Epidermal thickness.png]

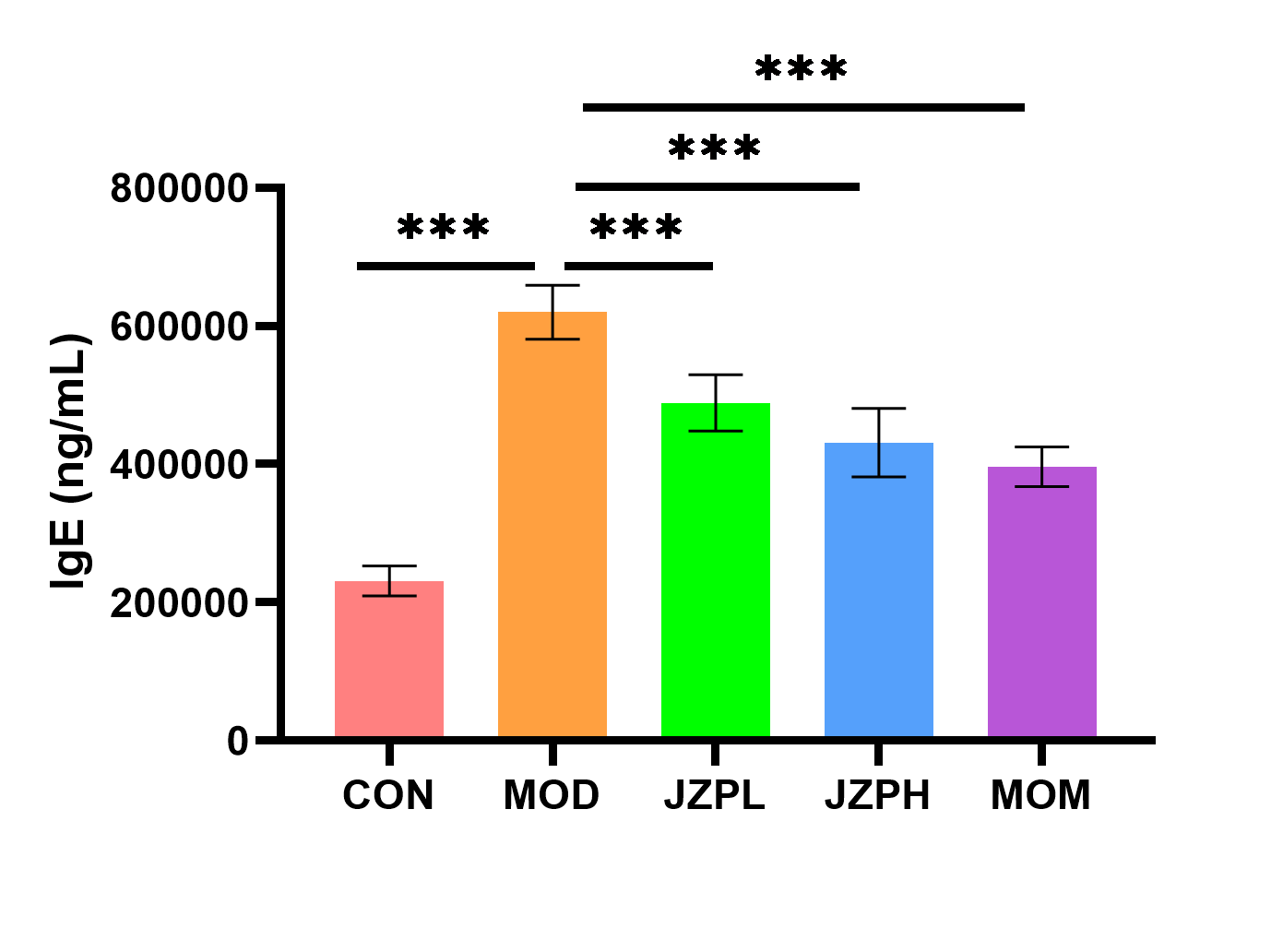

Supplement: Supplementary file 3 [file Data_Sheet_2.zip › Source data/FIGURE 2/lgE.png]

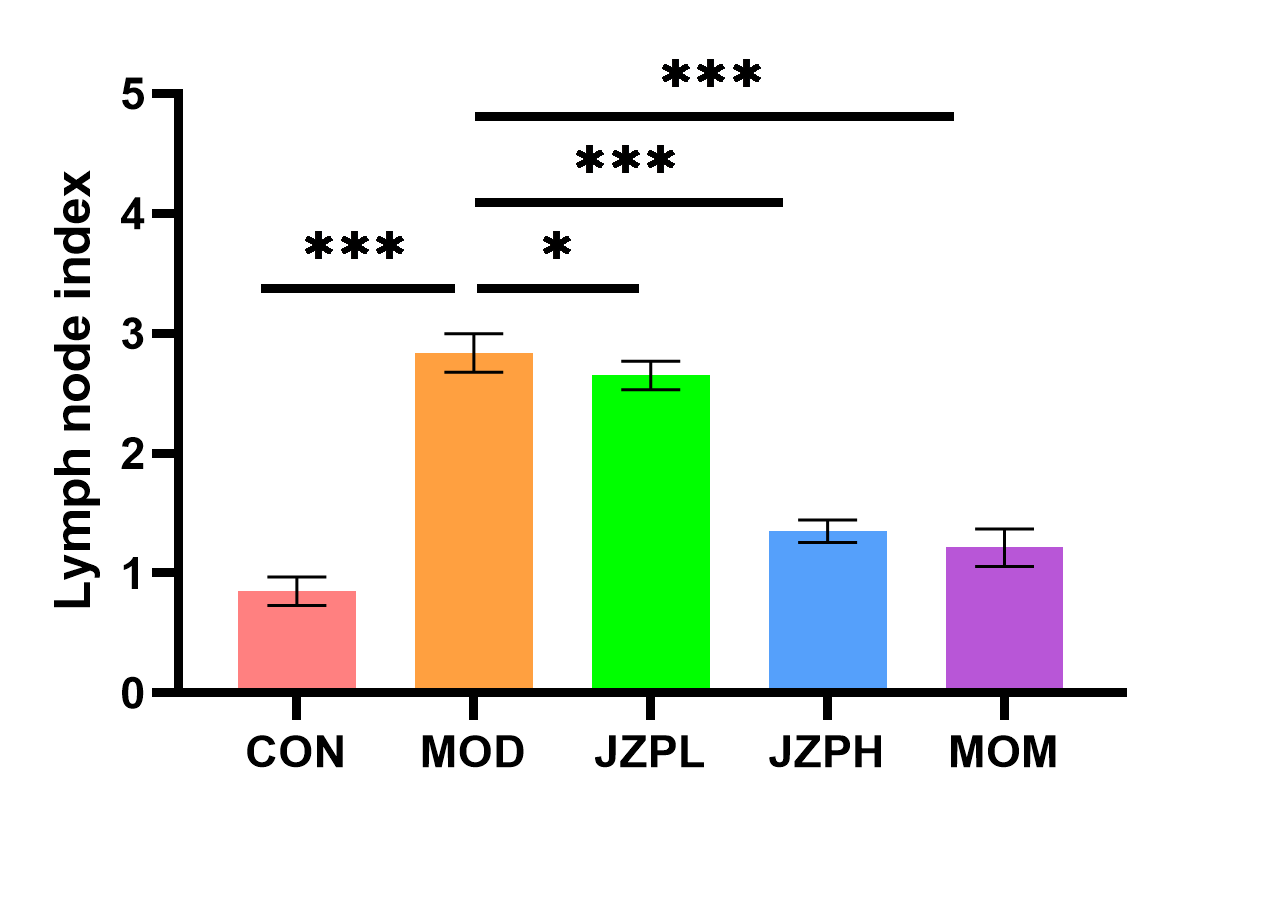

Supplement: Supplementary file 3 [file Data_Sheet_2.zip › Source data/FIGURE 2/Lymph node index.png]

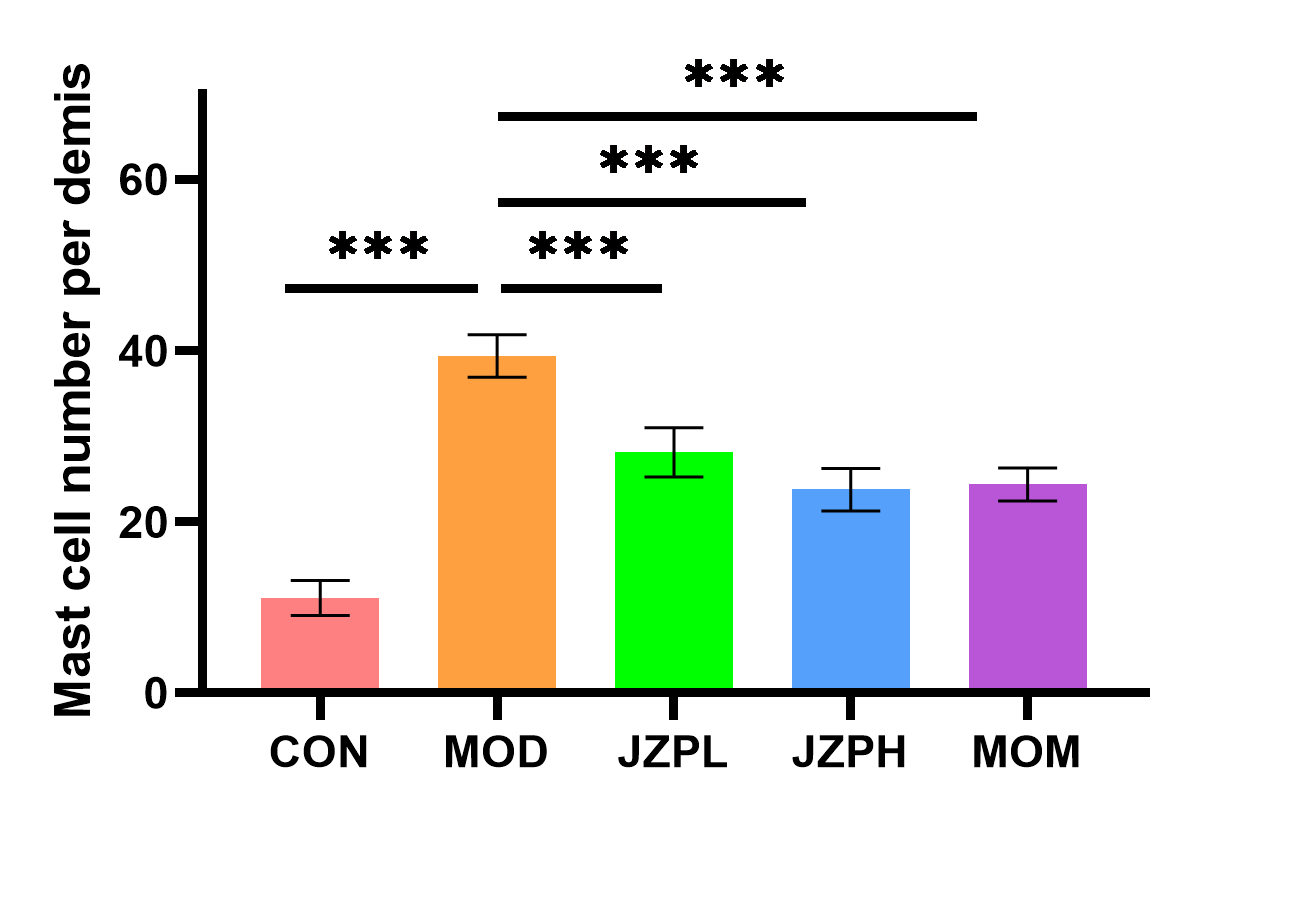

Supplement: Supplementary file 3 [file Data_Sheet_2.zip › Source data/FIGURE 2/Mast cell number per demis.png]

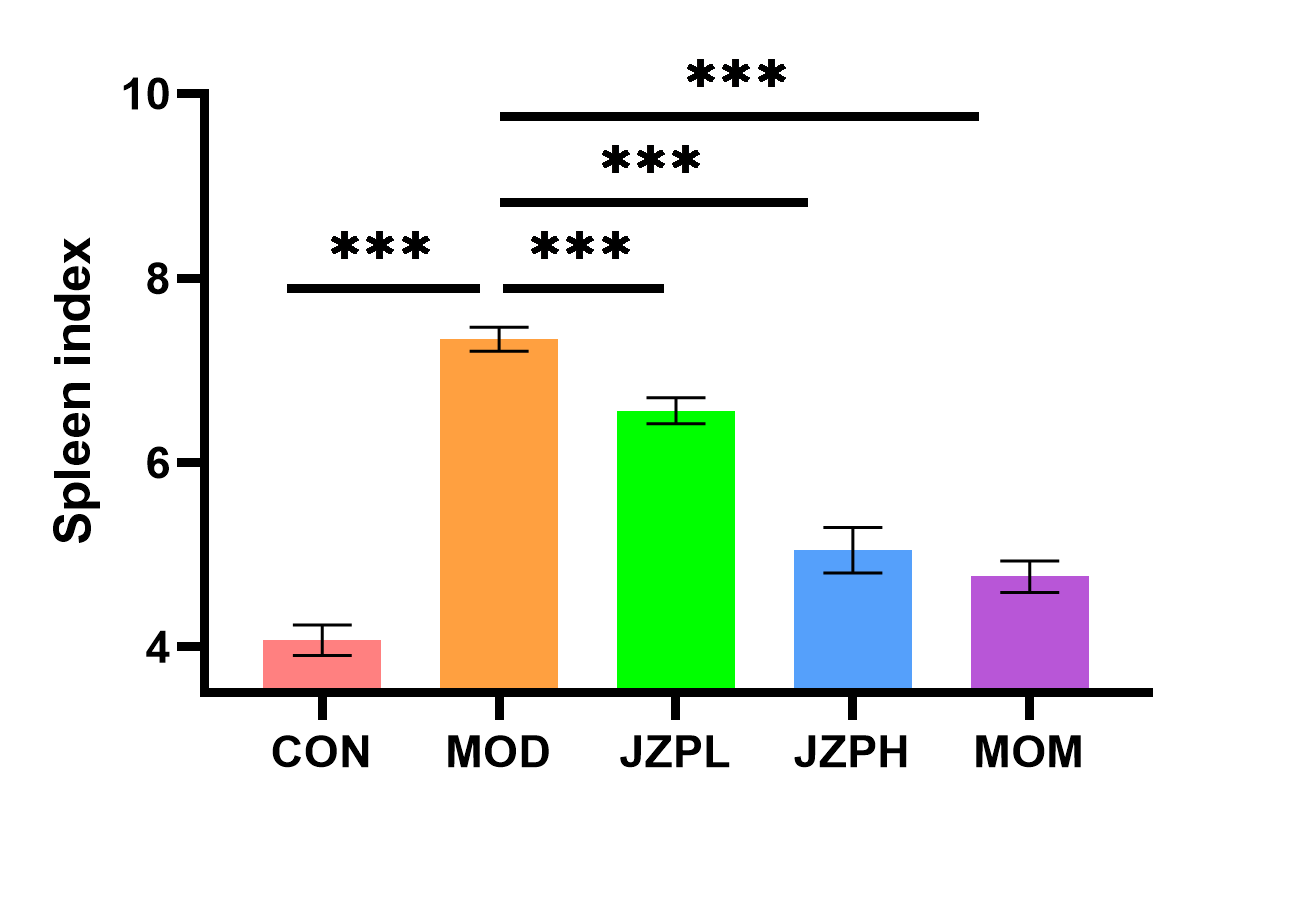

Supplement: Supplementary file 3 [file Data_Sheet_2.zip › Source data/FIGURE 2/Spleen index.png]

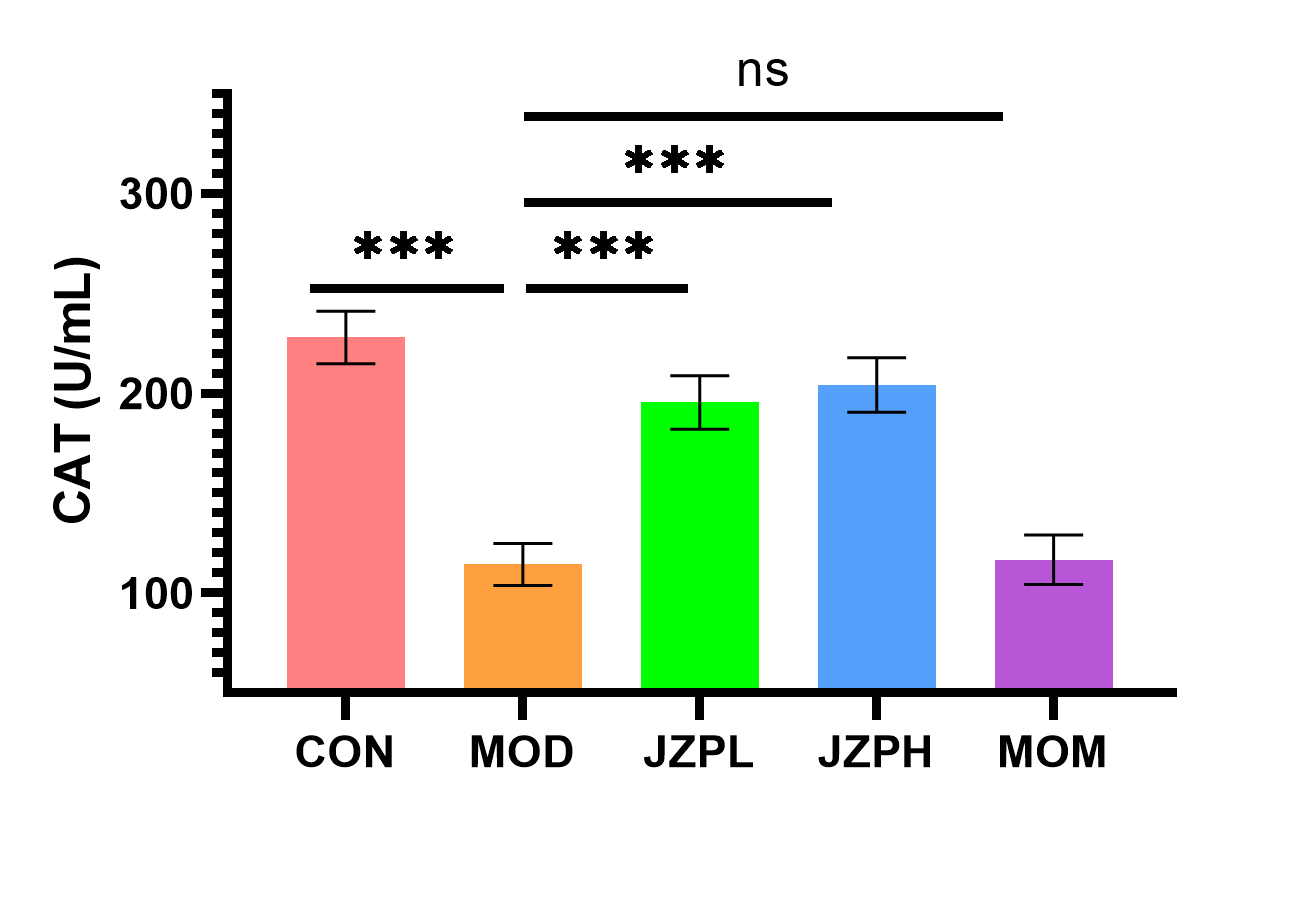

Supplement: Supplementary file 3 [file Data_Sheet_2.zip › Source data/FIGURE 3/CAT.png]

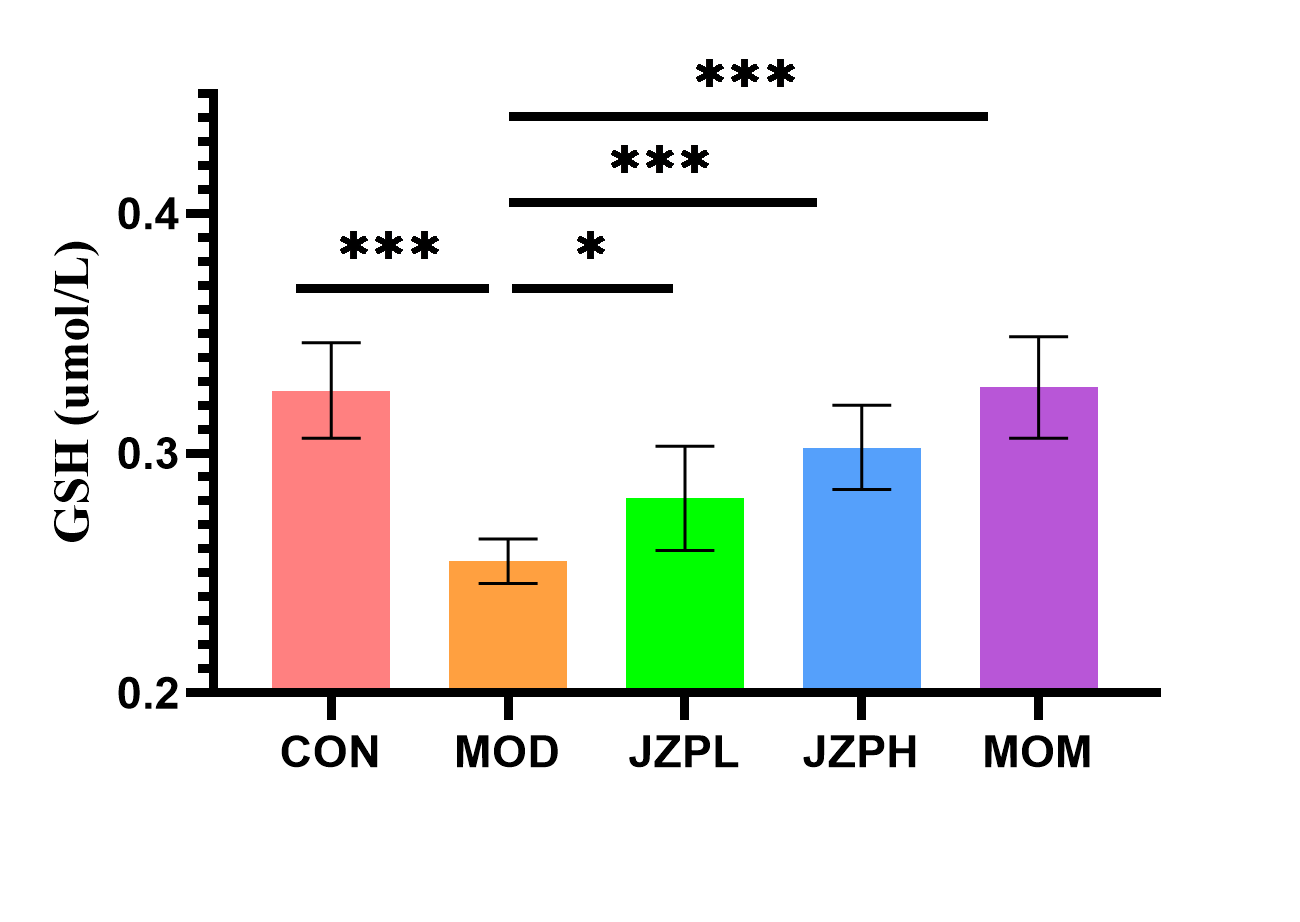

Supplement: Supplementary file 3 [file Data_Sheet_2.zip › Source data/FIGURE 3/GSH.png]

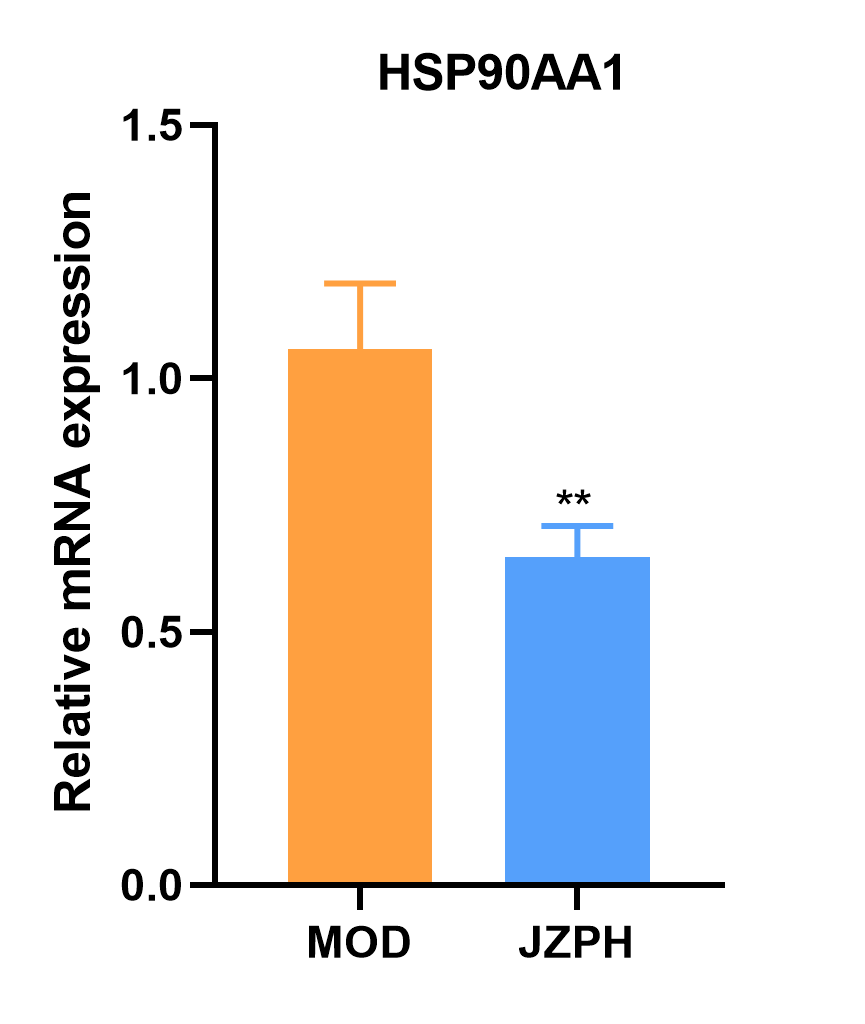

Supplement: Supplementary file 3 [file Data_Sheet_2.zip › Source data/FIGURE 3/HSP90AA1.tif]

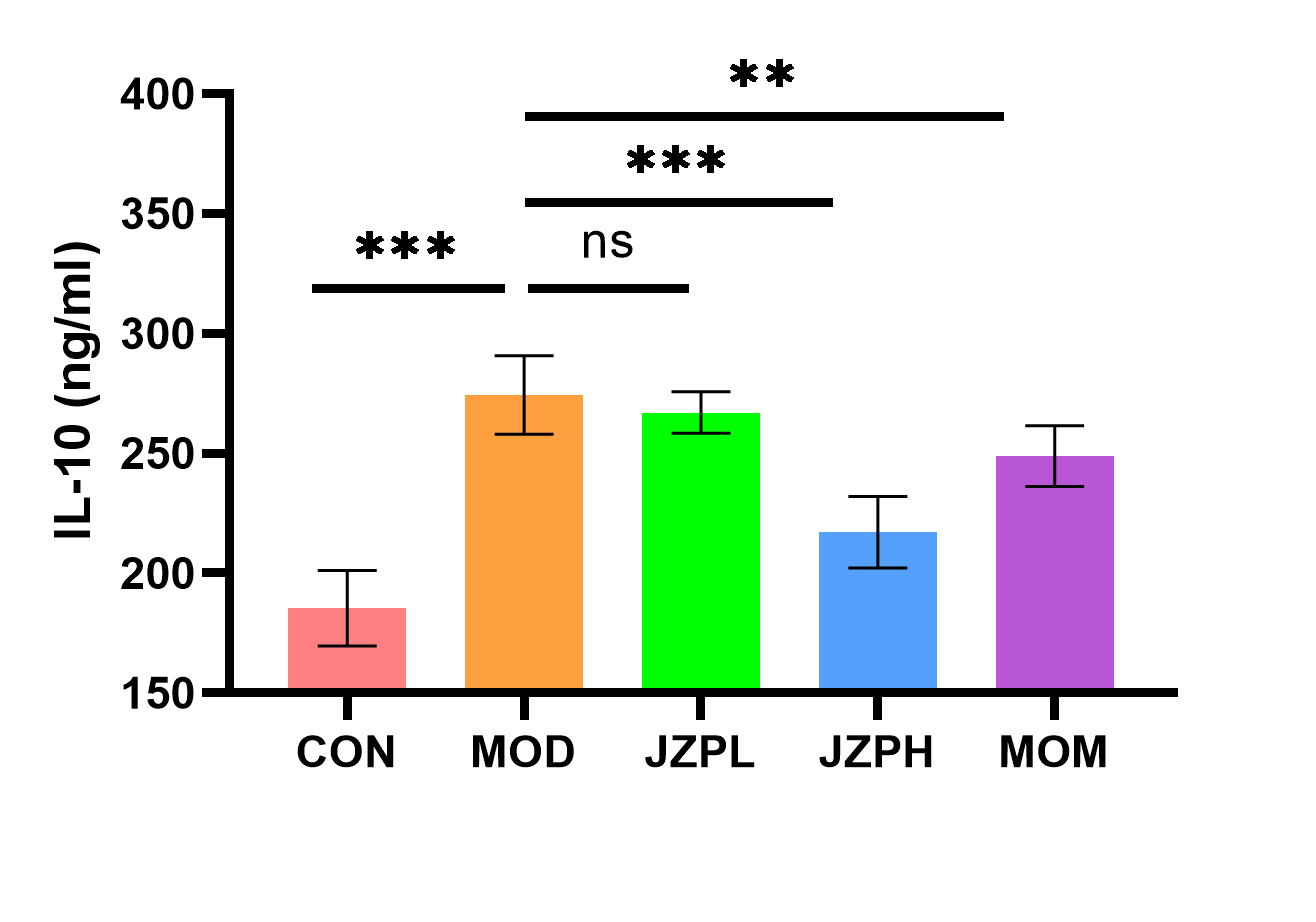

Supplement: Supplementary file 3 [file Data_Sheet_2.zip › Source data/FIGURE 3/IL-10.png]

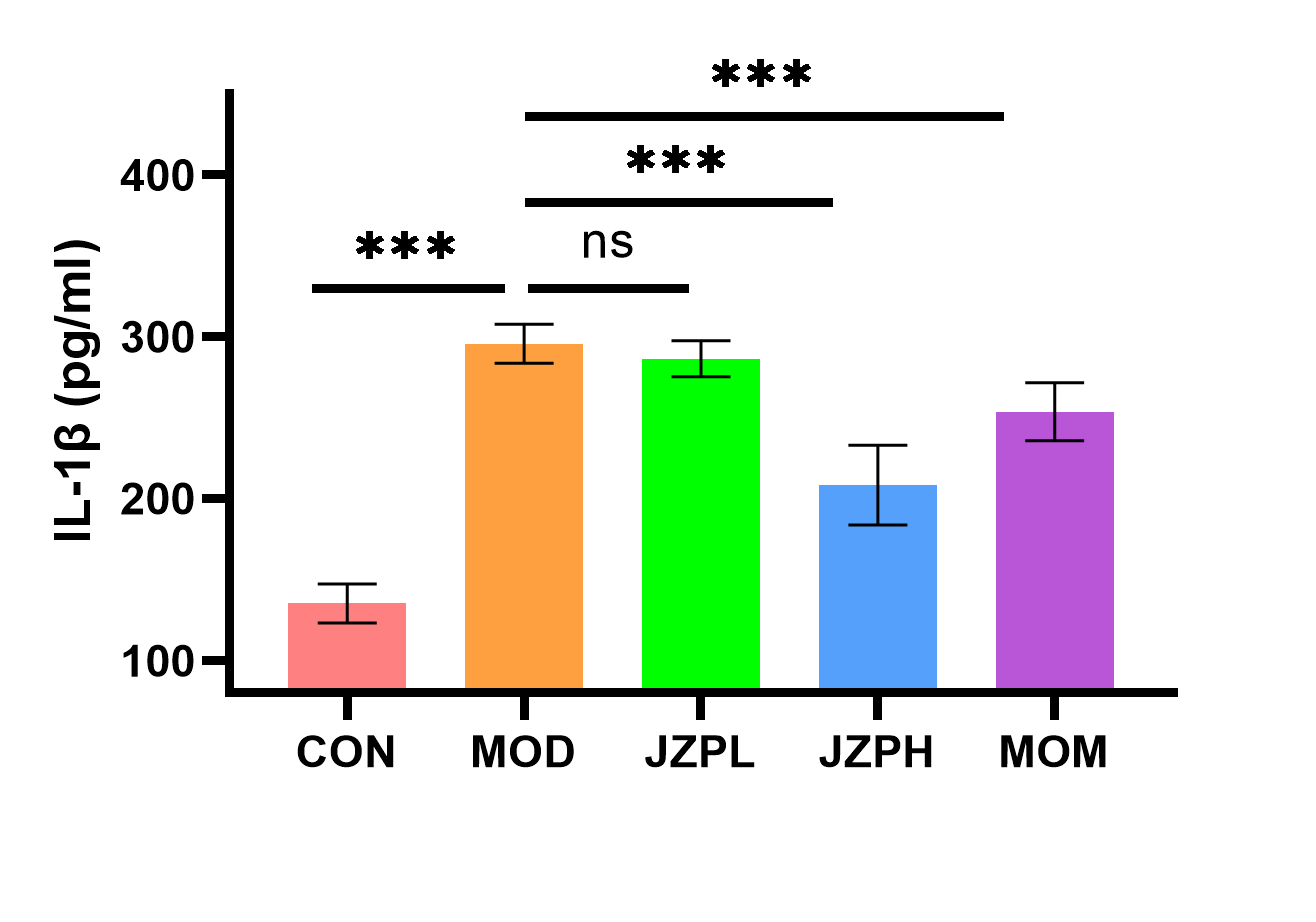

Supplement: Supplementary file 3 [file Data_Sheet_2.zip › Source data/FIGURE 3/IL-1β.png]

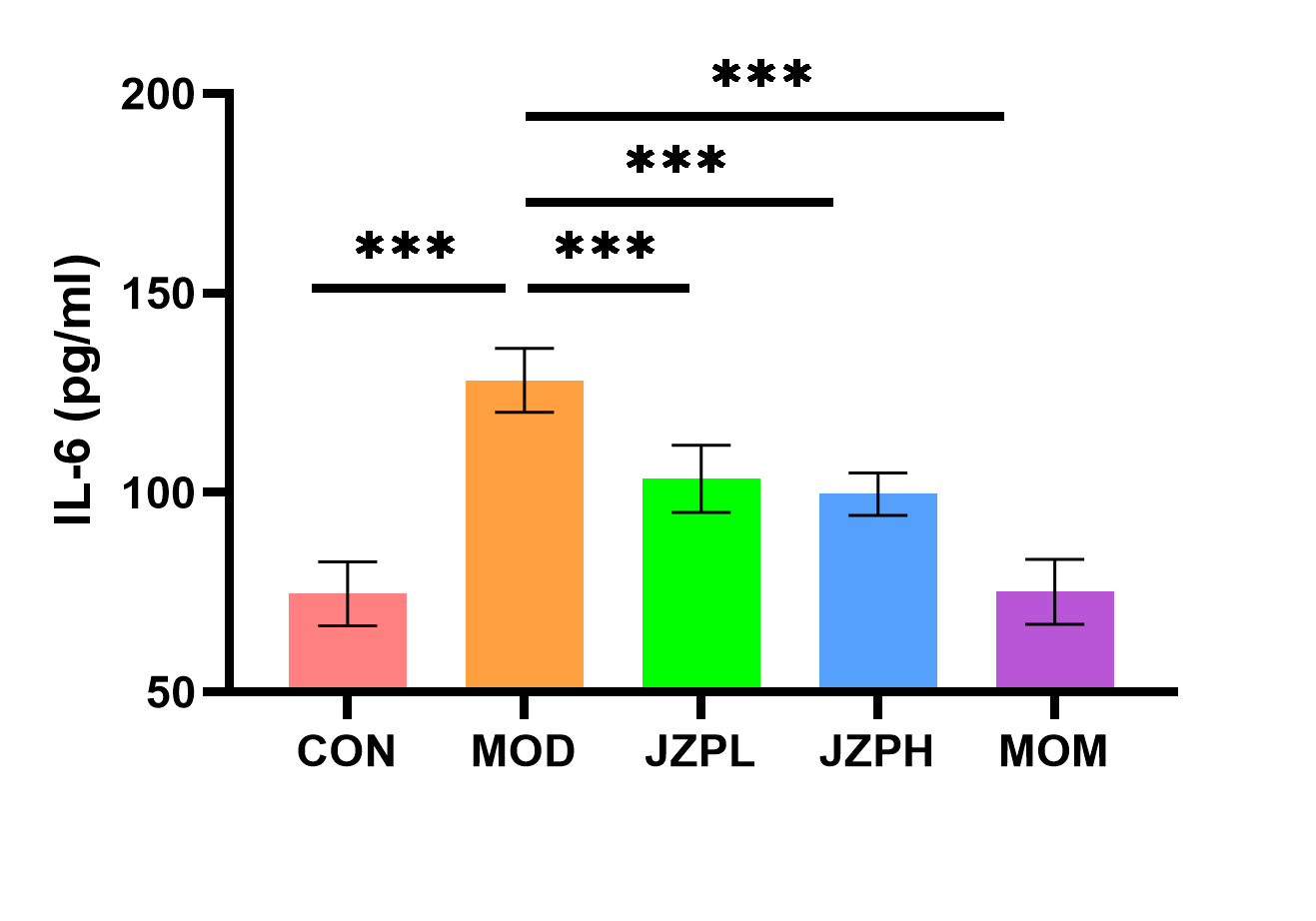

Supplement: Supplementary file 3 [file Data_Sheet_2.zip › Source data/FIGURE 3/IL-6.png]

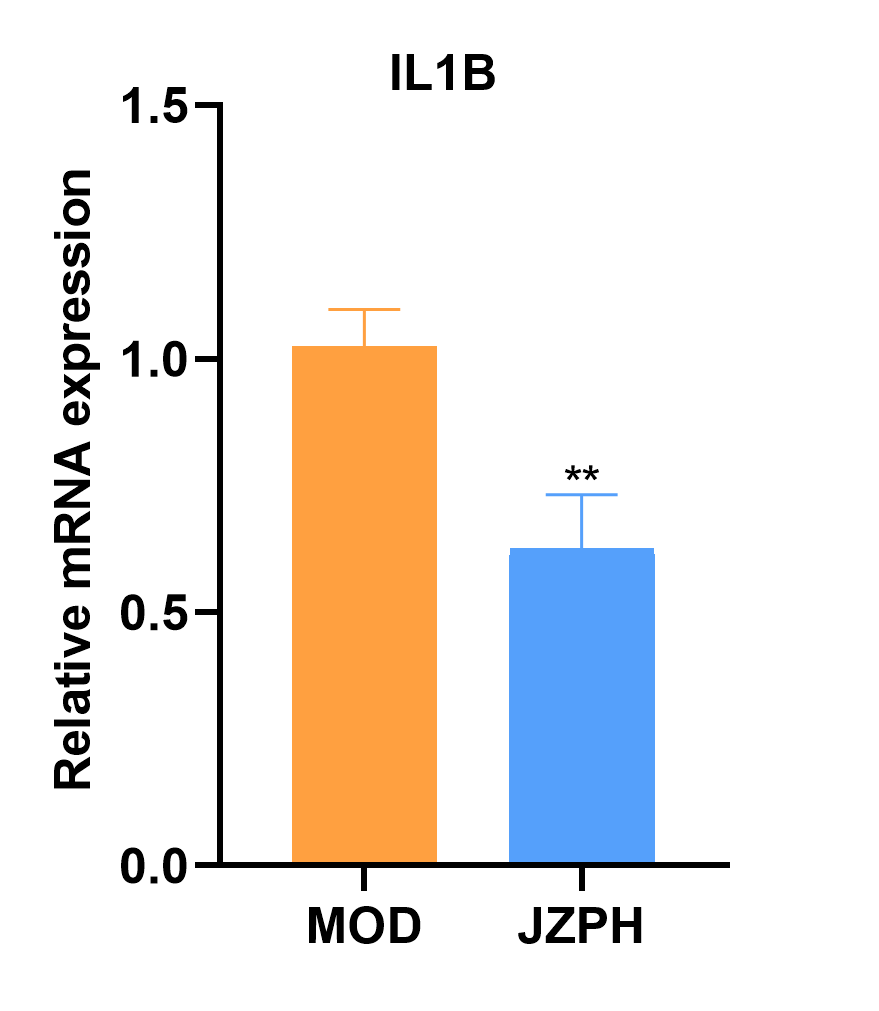

Supplement: Supplementary file 3 [file Data_Sheet_2.zip › Source data/FIGURE 3/IL1B.tif]

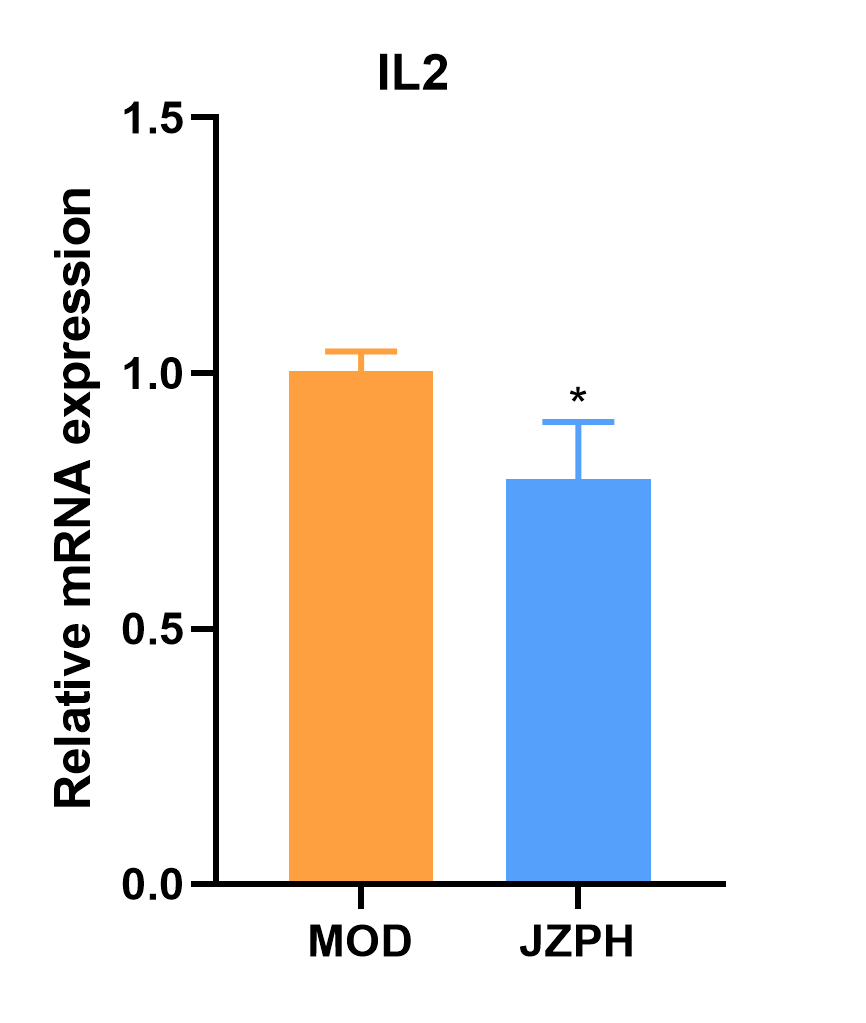

Supplement: Supplementary file 3 [file Data_Sheet_2.zip › Source data/FIGURE 3/IL2.tif]

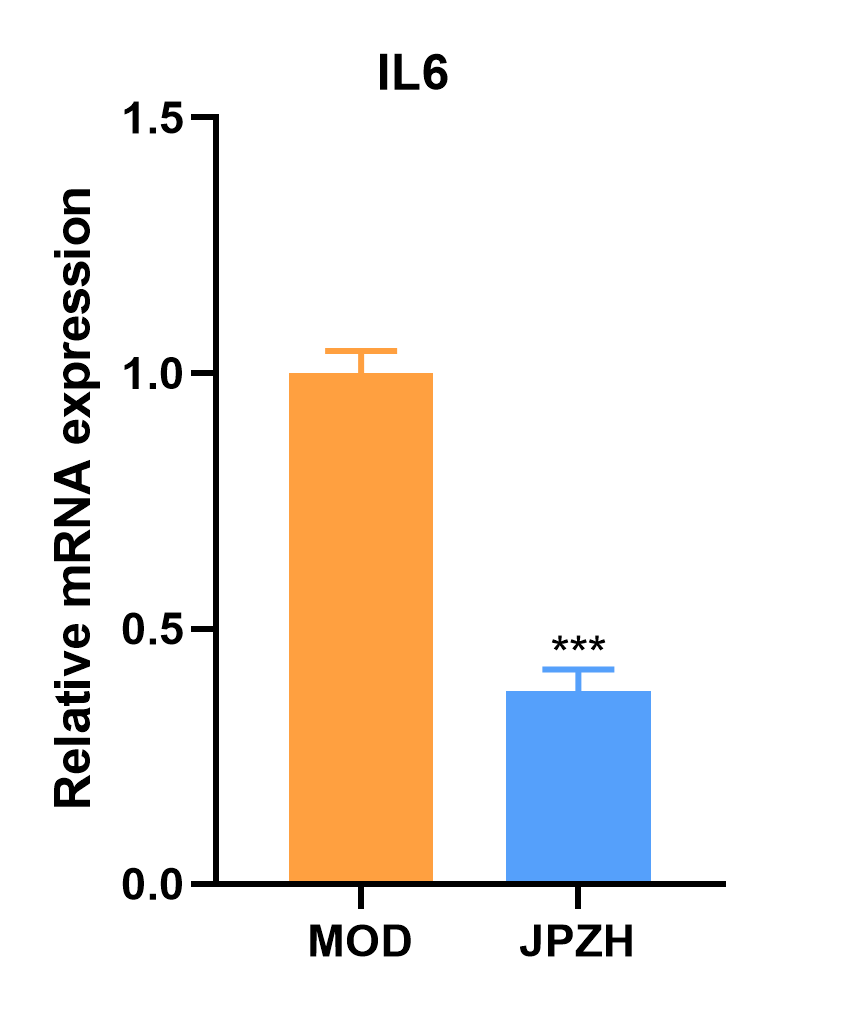

Supplement: Supplementary file 3 [file Data_Sheet_2.zip › Source data/FIGURE 3/IL6.tif]

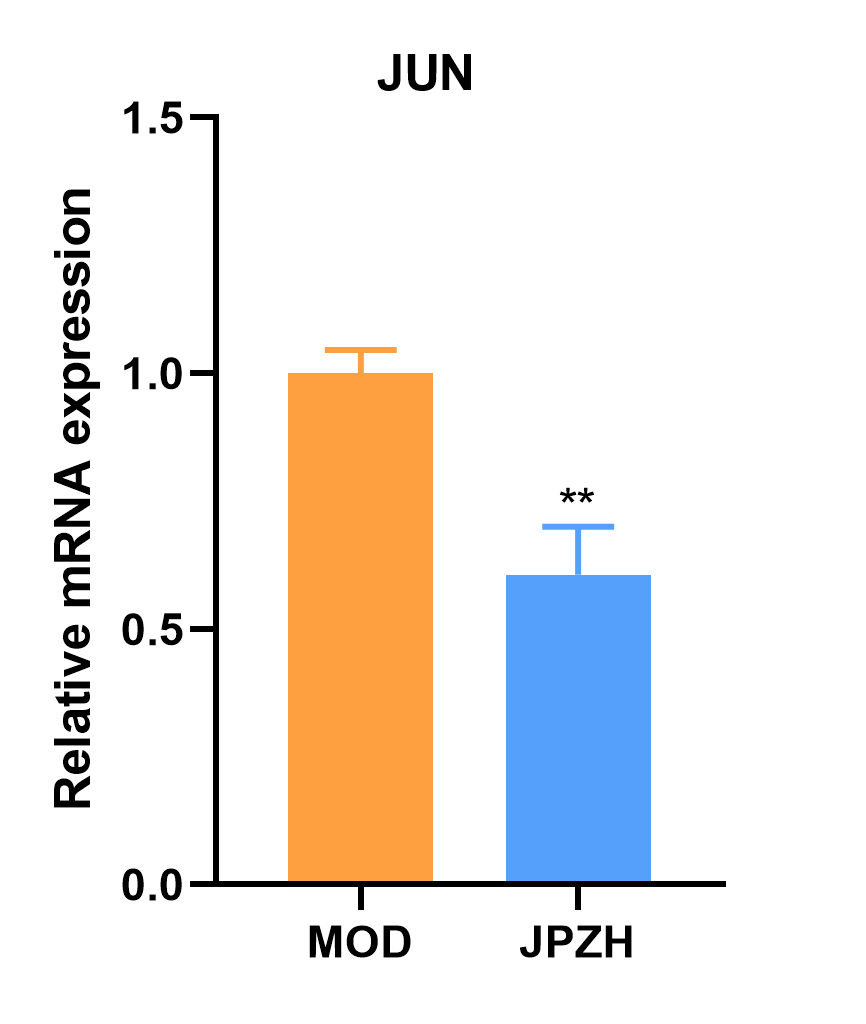

Supplement: Supplementary file 3 [file Data_Sheet_2.zip › Source data/FIGURE 3/JUN.tif]

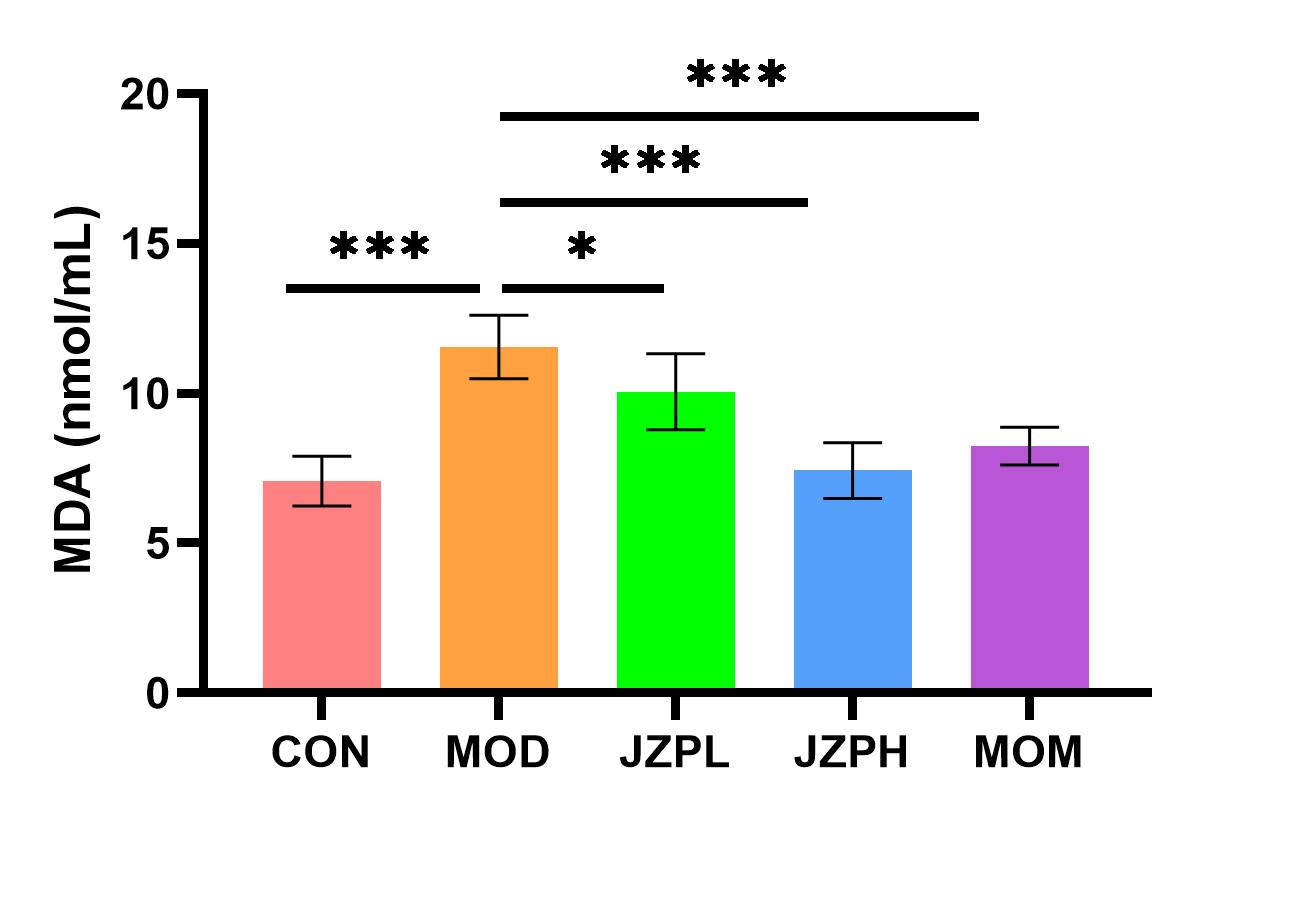

Supplement: Supplementary file 3 [file Data_Sheet_2.zip › Source data/FIGURE 3/MDA.png]

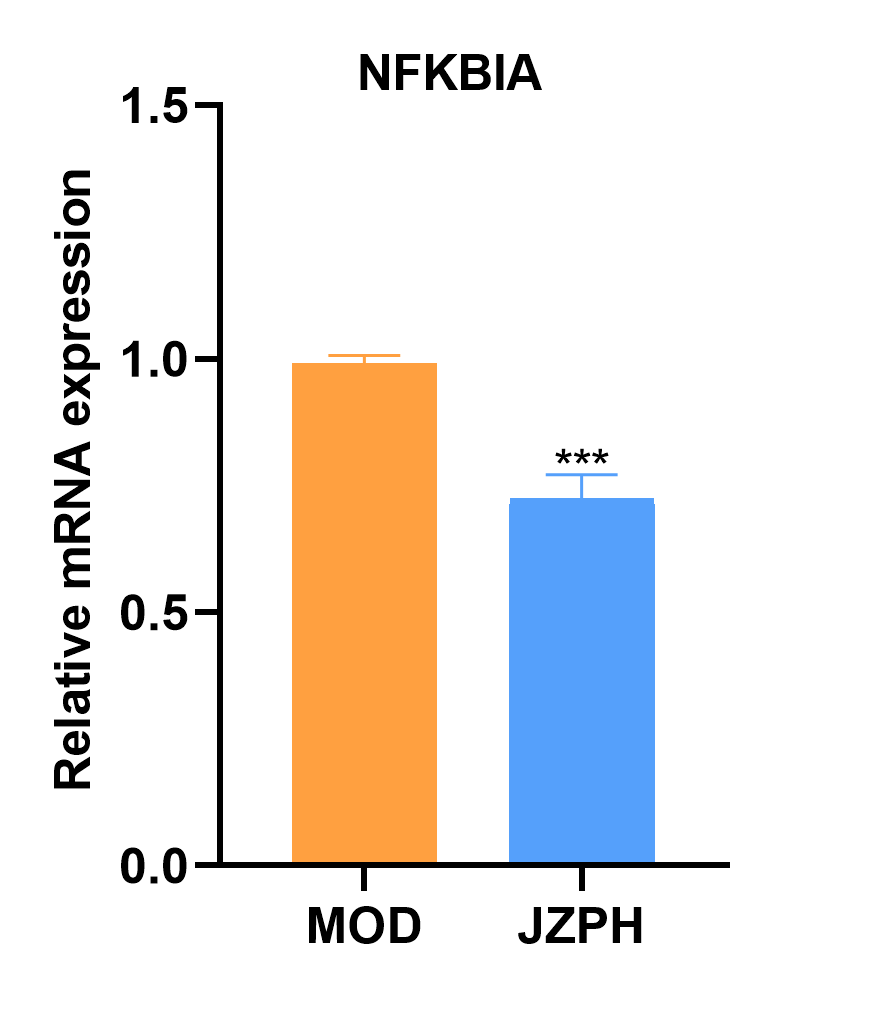

Supplement: Supplementary file 3 [file Data_Sheet_2.zip › Source data/FIGURE 3/NFKBIA.tif]

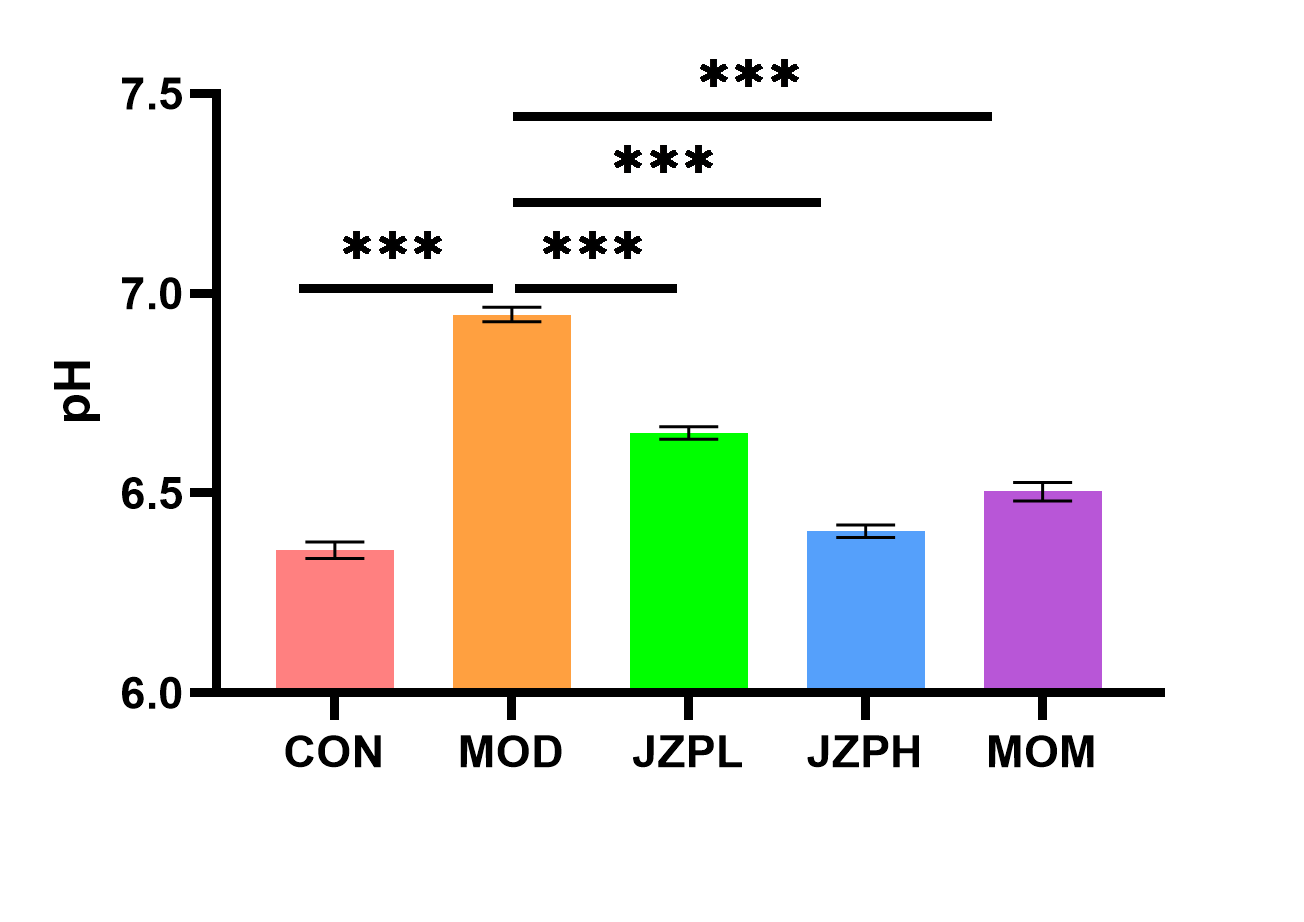

Supplement: Supplementary file 3 [file Data_Sheet_2.zip › Source data/FIGURE 3/pH.png]

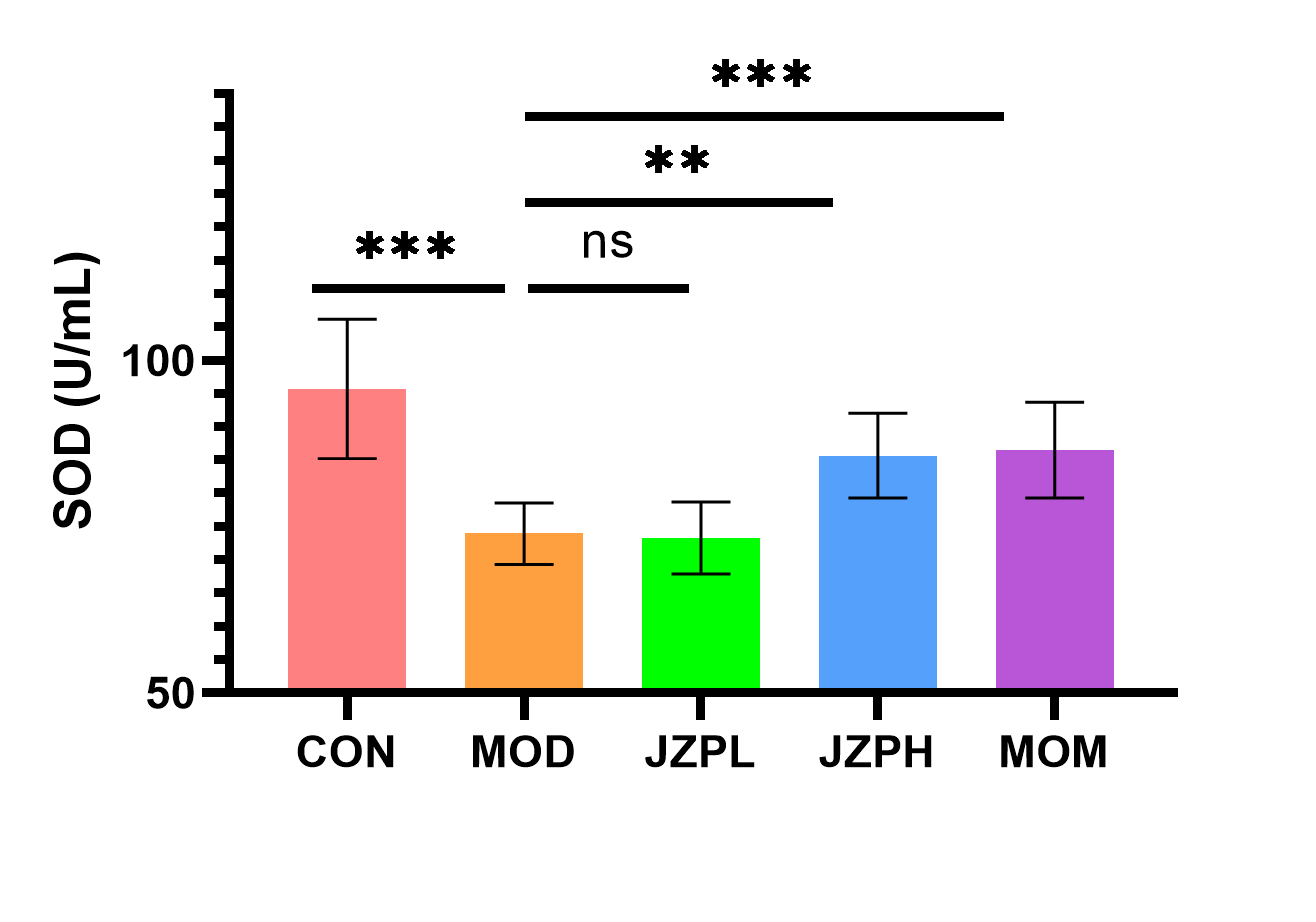

Supplement: Supplementary file 3 [file Data_Sheet_2.zip › Source data/FIGURE 3/SOD.png]

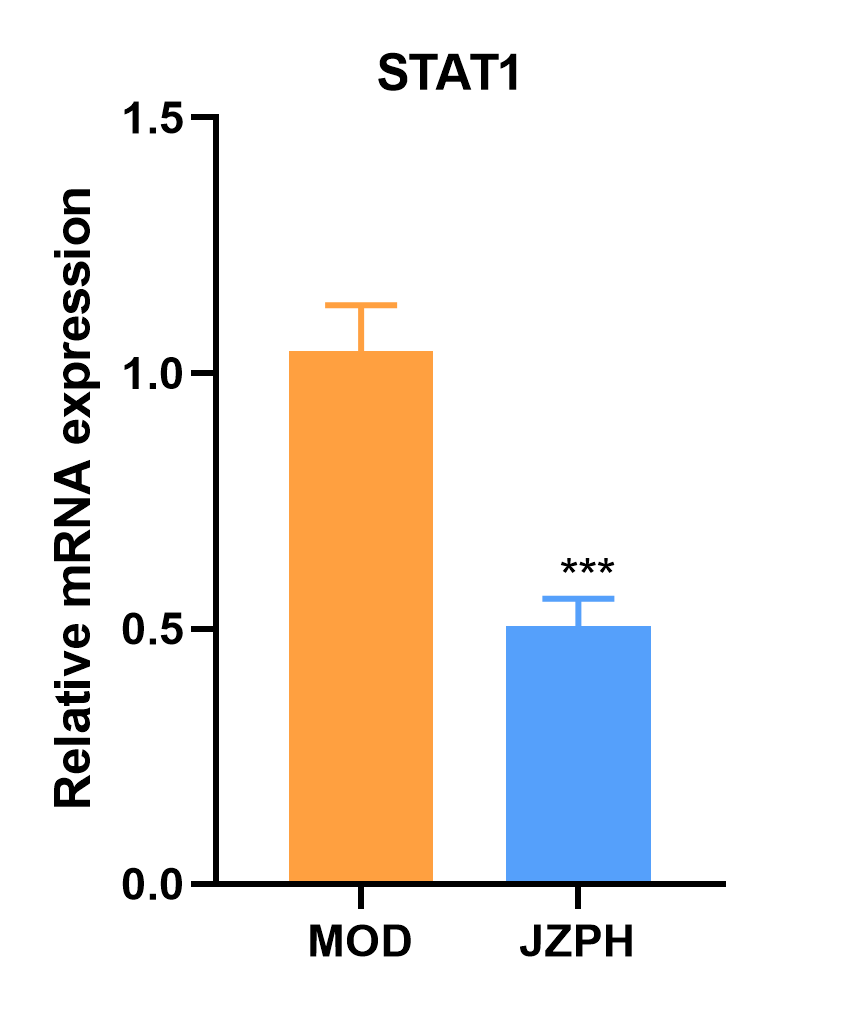

Supplement: Supplementary file 3 [file Data_Sheet_2.zip › Source data/FIGURE 3/STAT1.tif]

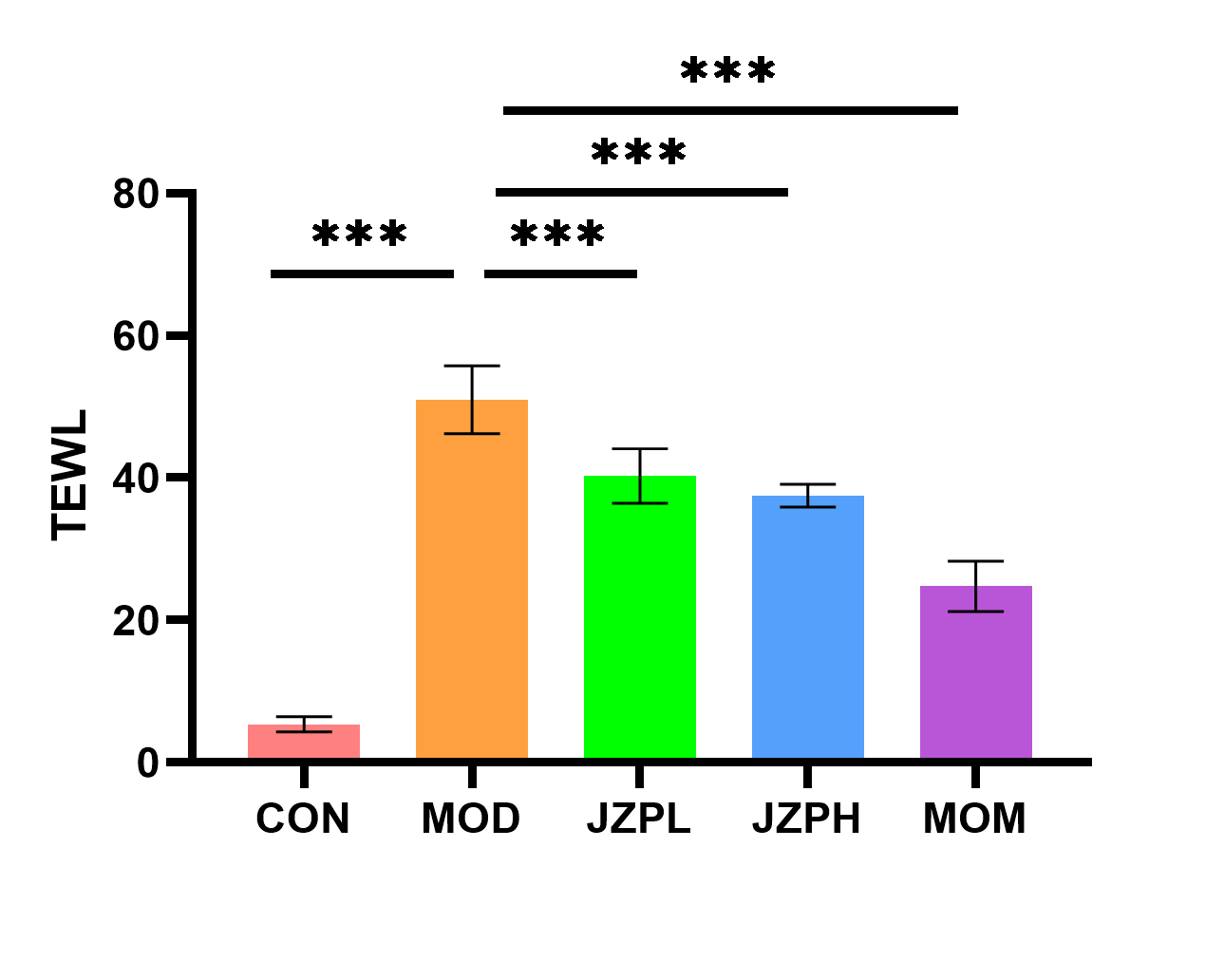

Supplement: Supplementary file 3 [file Data_Sheet_2.zip › Source data/FIGURE 3/TEWL.png]

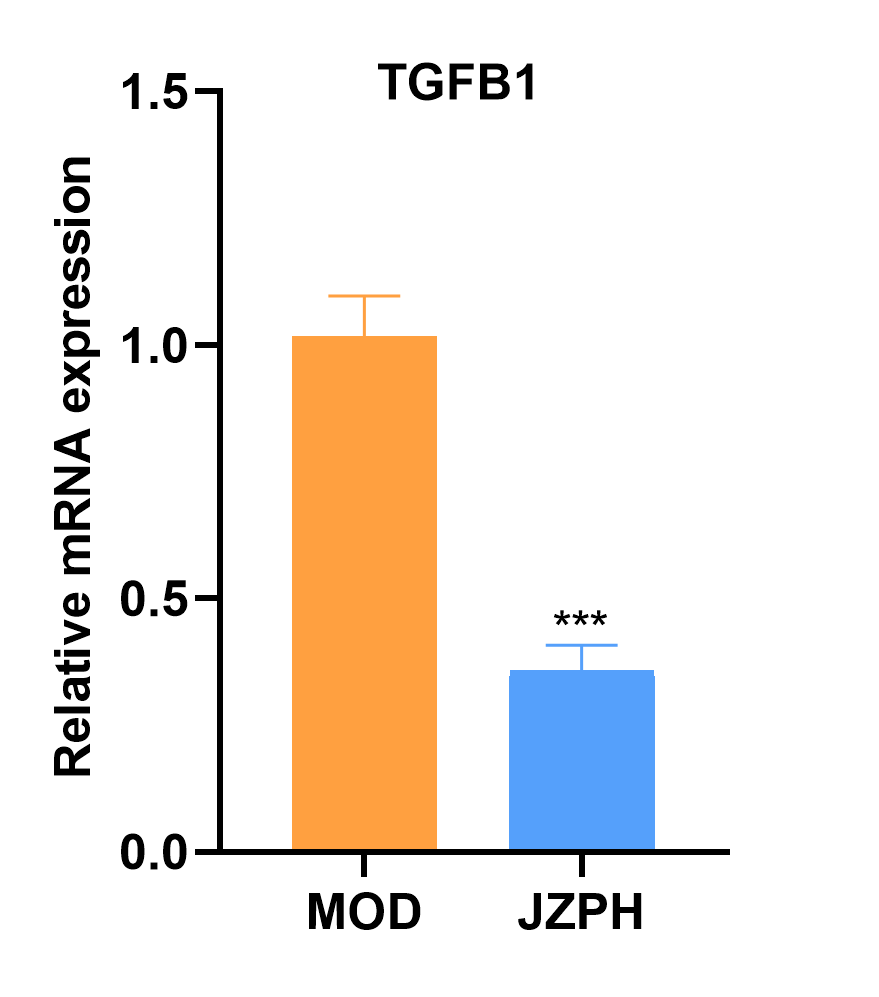

Supplement: Supplementary file 3 [file Data_Sheet_2.zip › Source data/FIGURE 3/TGFB1.tif]

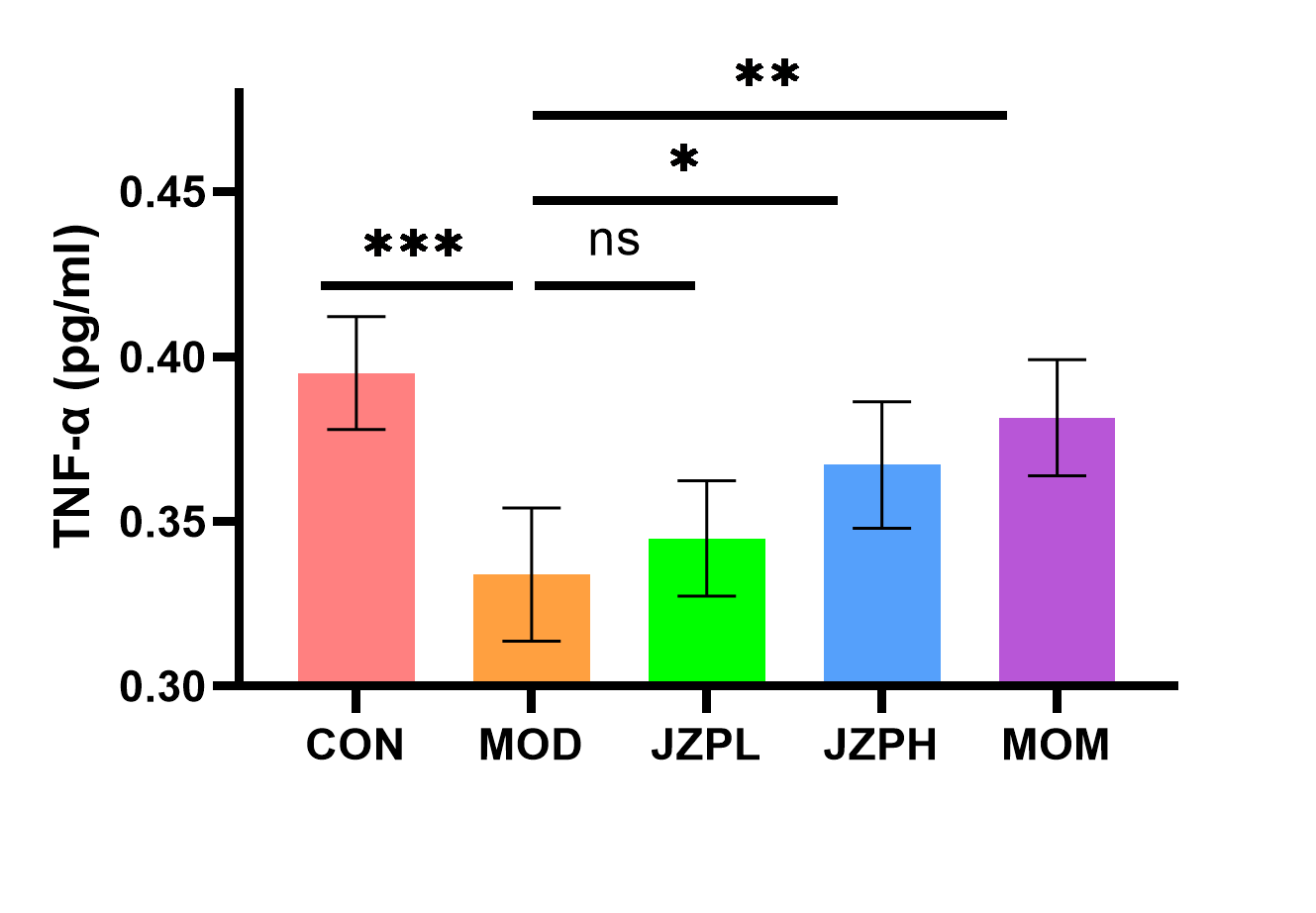

Supplement: Supplementary file 3 [file Data_Sheet_2.zip › Source data/FIGURE 3/TNF-α.png]

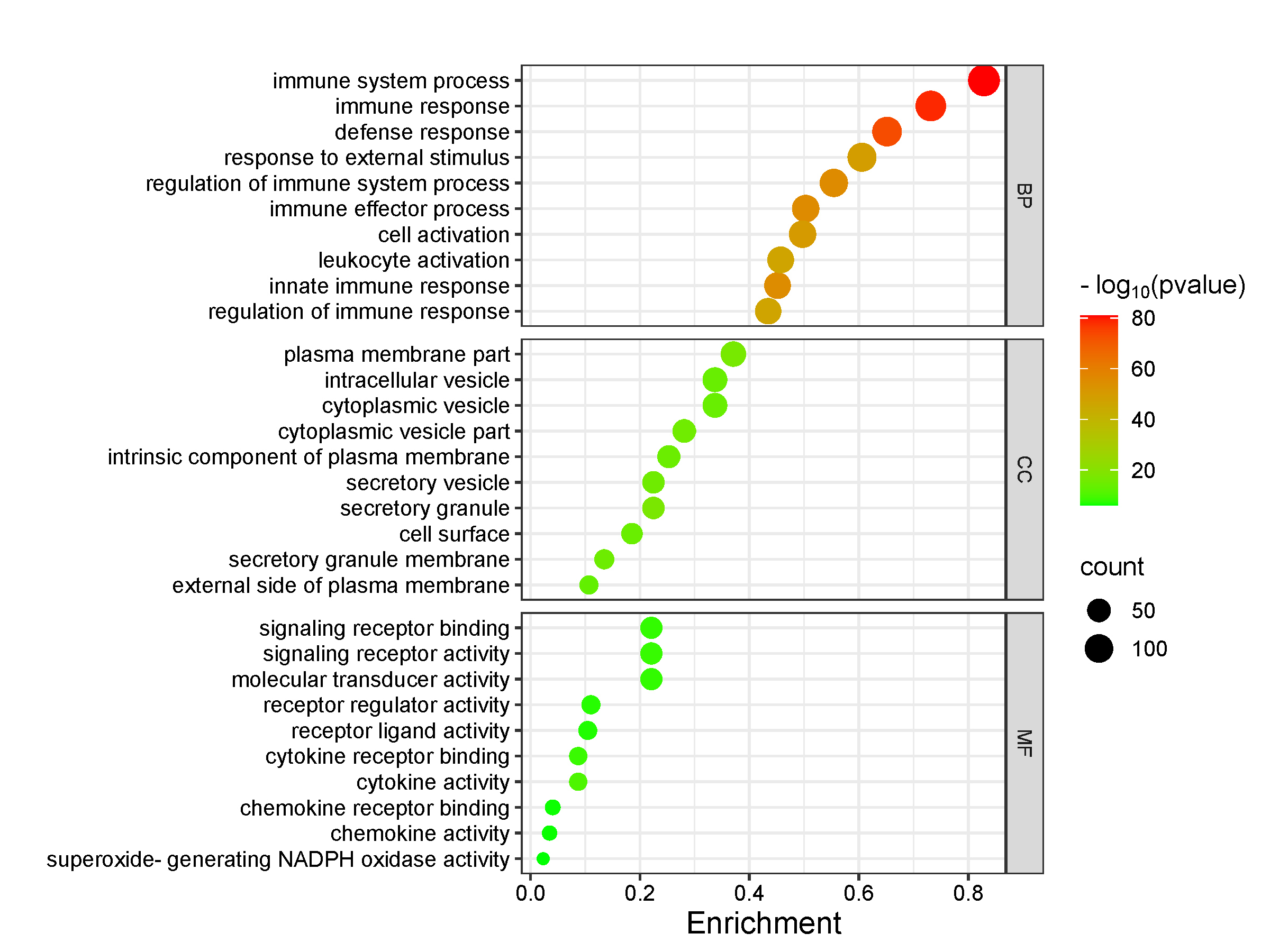

Supplement: Supplementary file 3 [file Data_Sheet_2.zip › Source data/FIGURE 4/GO.png]

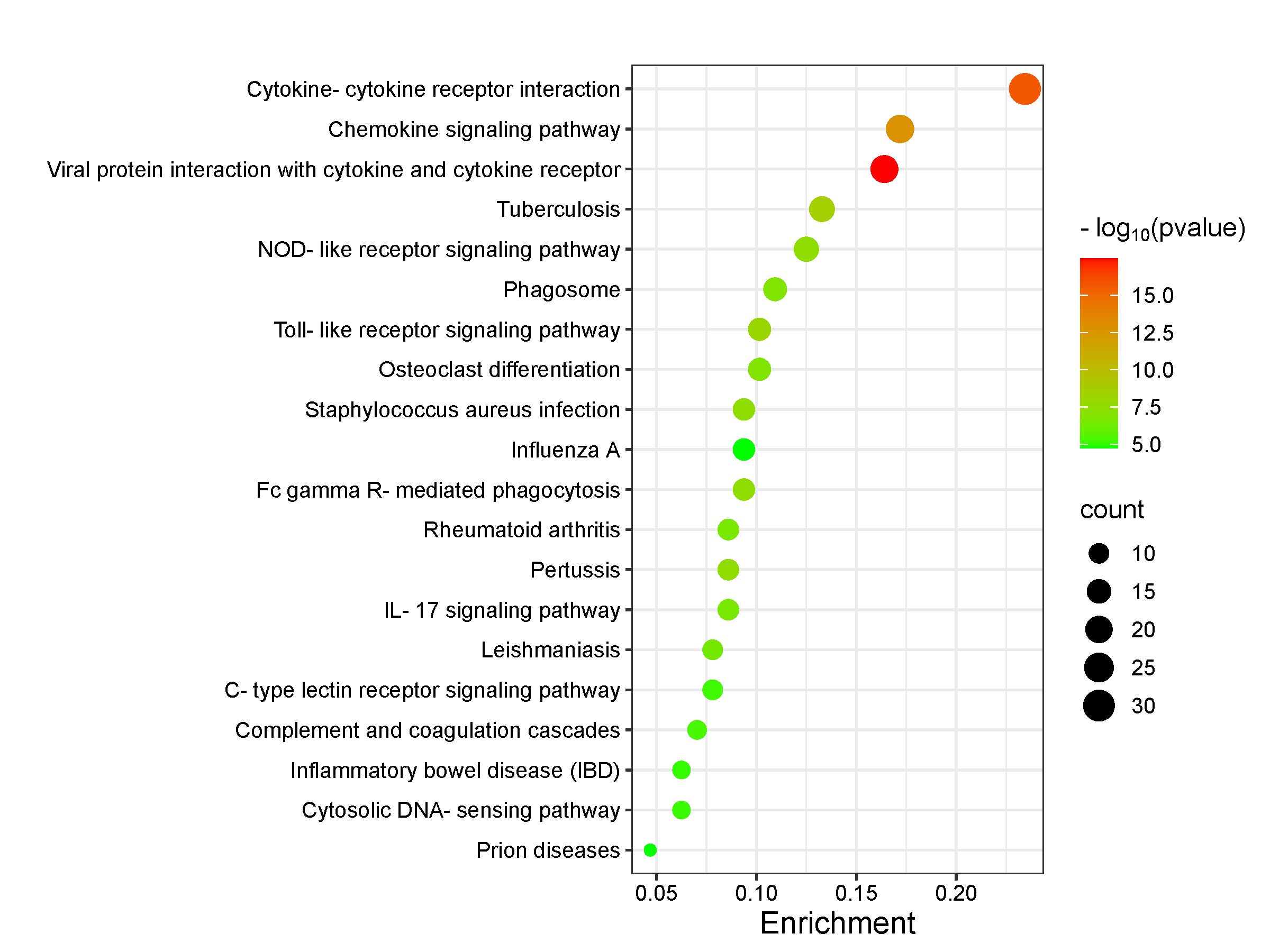

Supplement: Supplementary file 3 [file Data_Sheet_2.zip › Source data/FIGURE 4/KEGG.png]

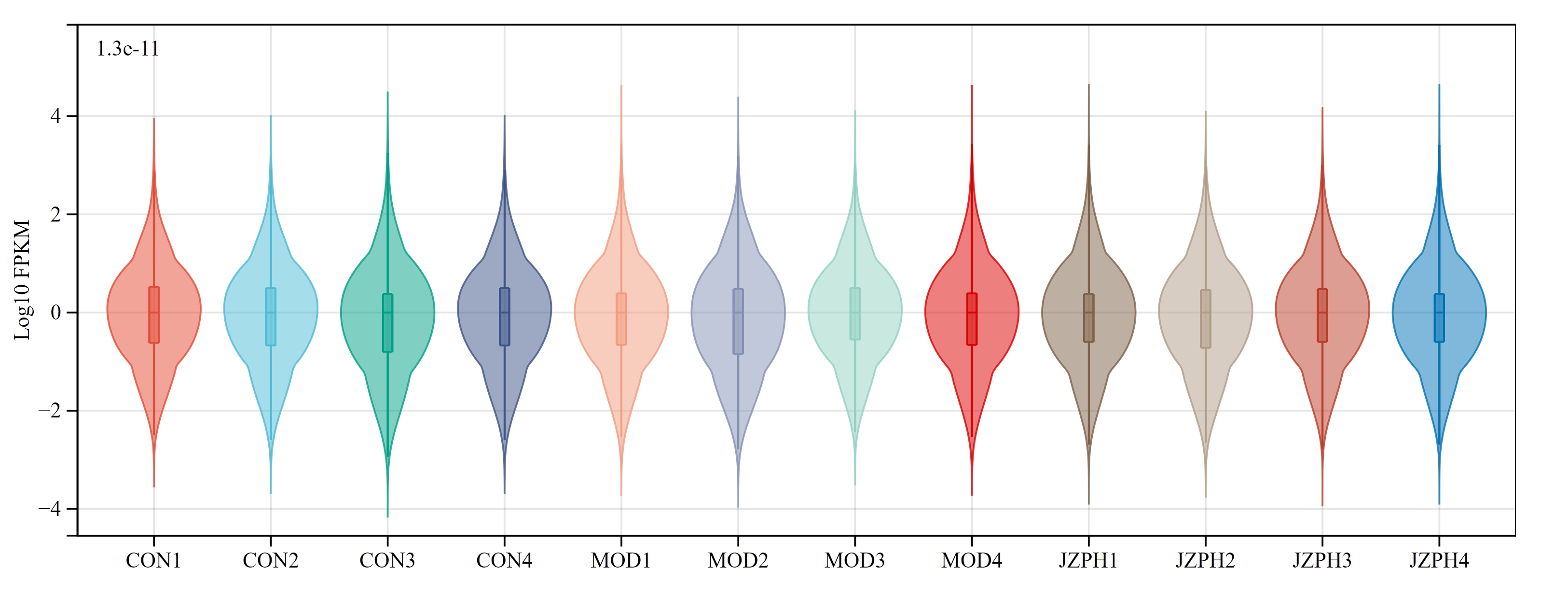

Supplement: Supplementary file 3 [file Data_Sheet_2.zip › Source data/FIGURE 4/LOG10 FPKM.jpeg]

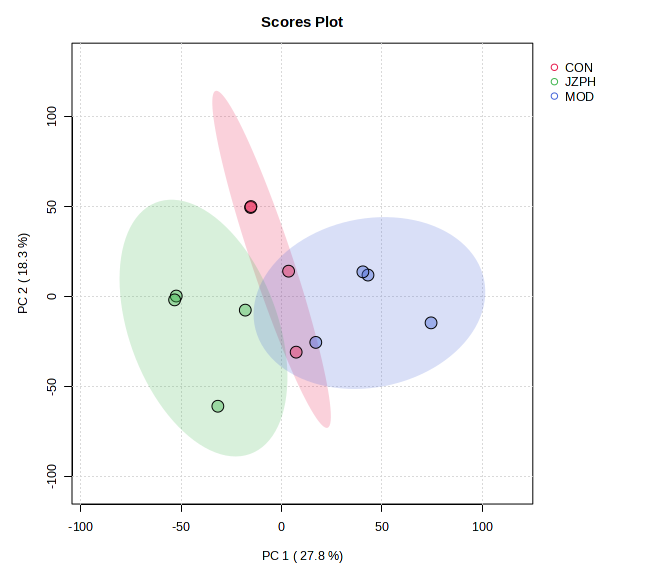

Supplement: Supplementary file 3 [file Data_Sheet_2.zip › Source data/FIGURE 4/PCA.png]

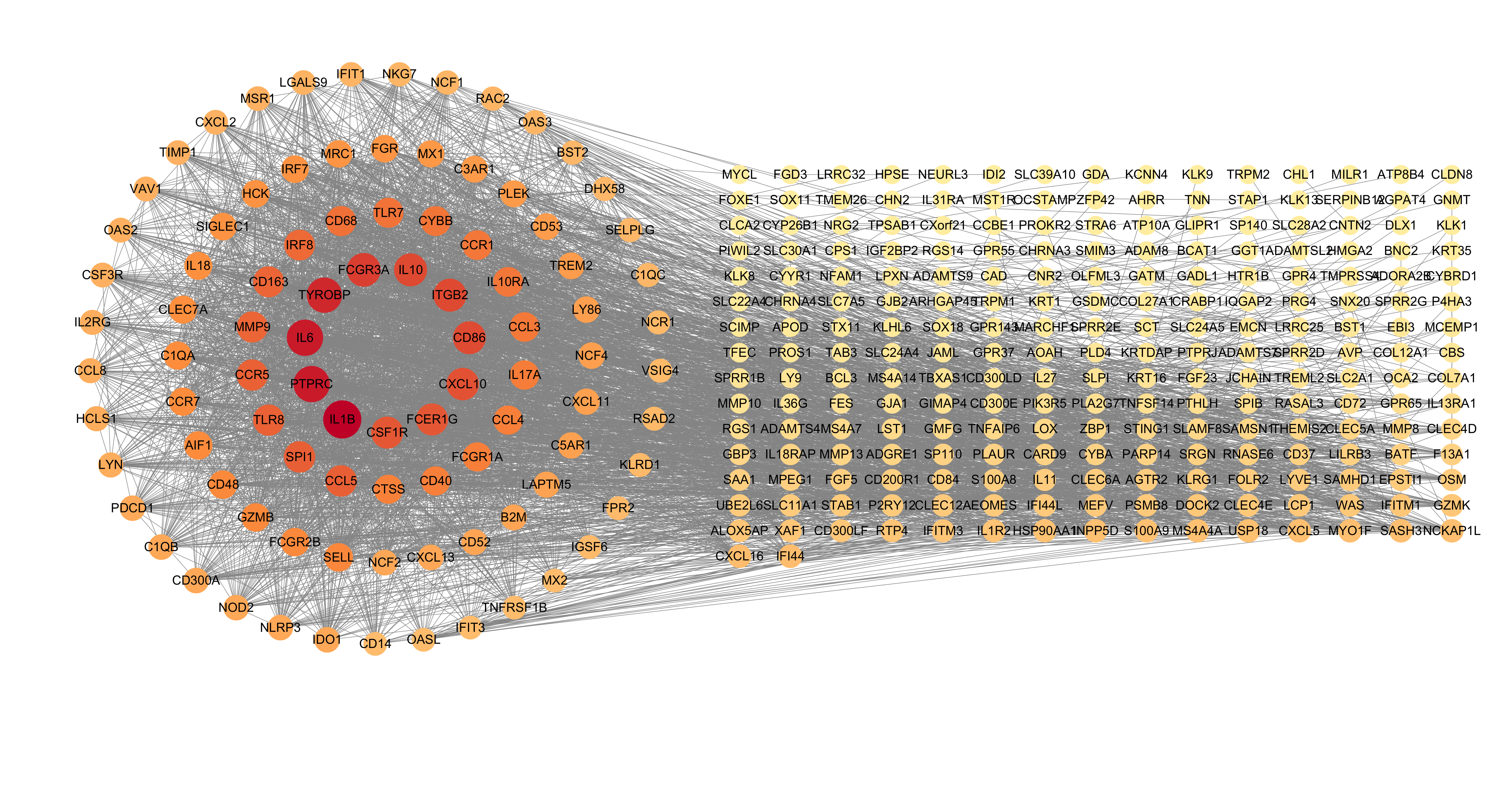

Supplement: Supplementary file 3 [file Data_Sheet_2.zip › Source data/FIGURE 4/PPI.png]

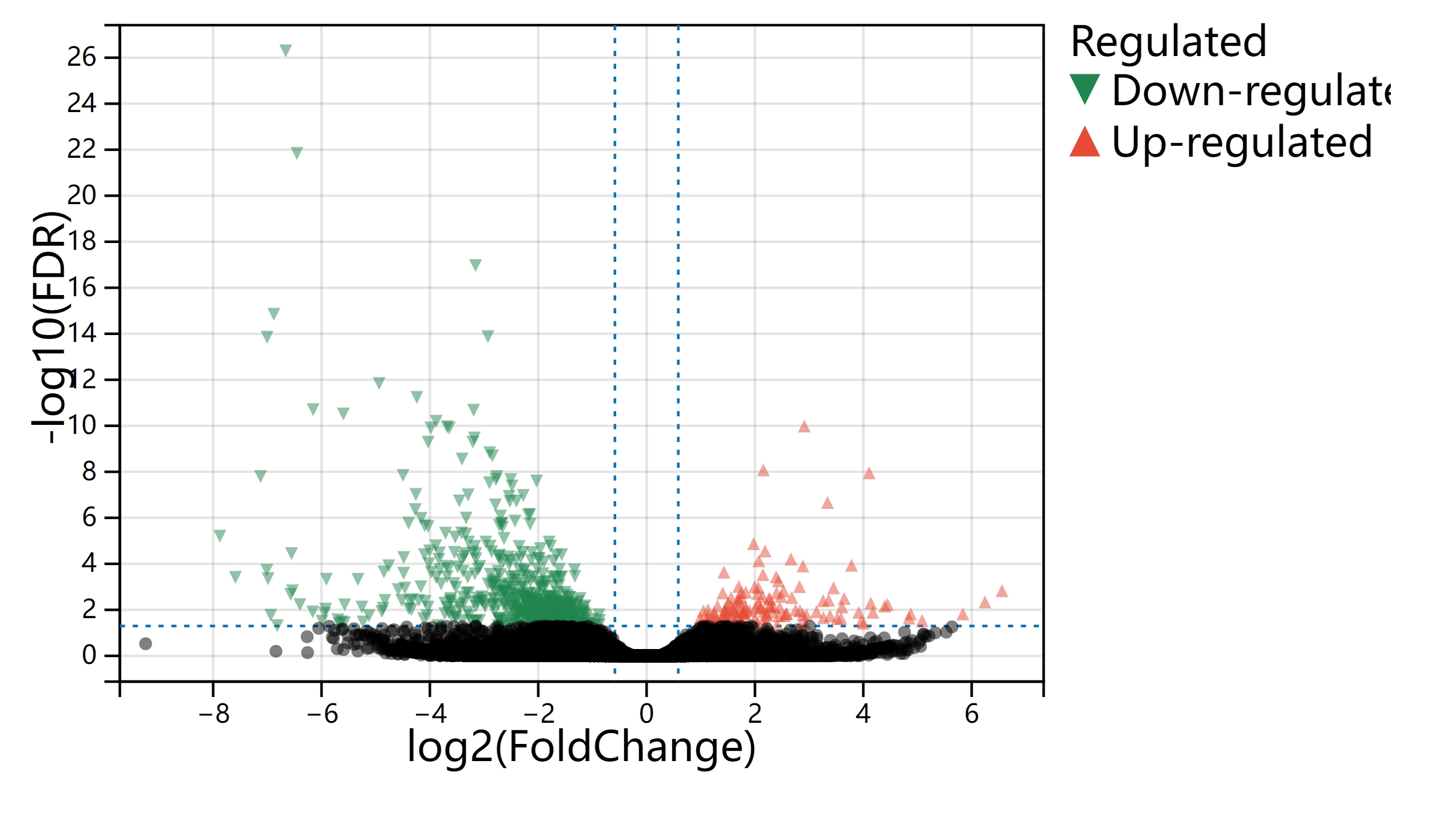

Supplement: Supplementary file 3 [file Data_Sheet_2.zip › Source data/FIGURE 4/result-c&m-chuli .jpeg]

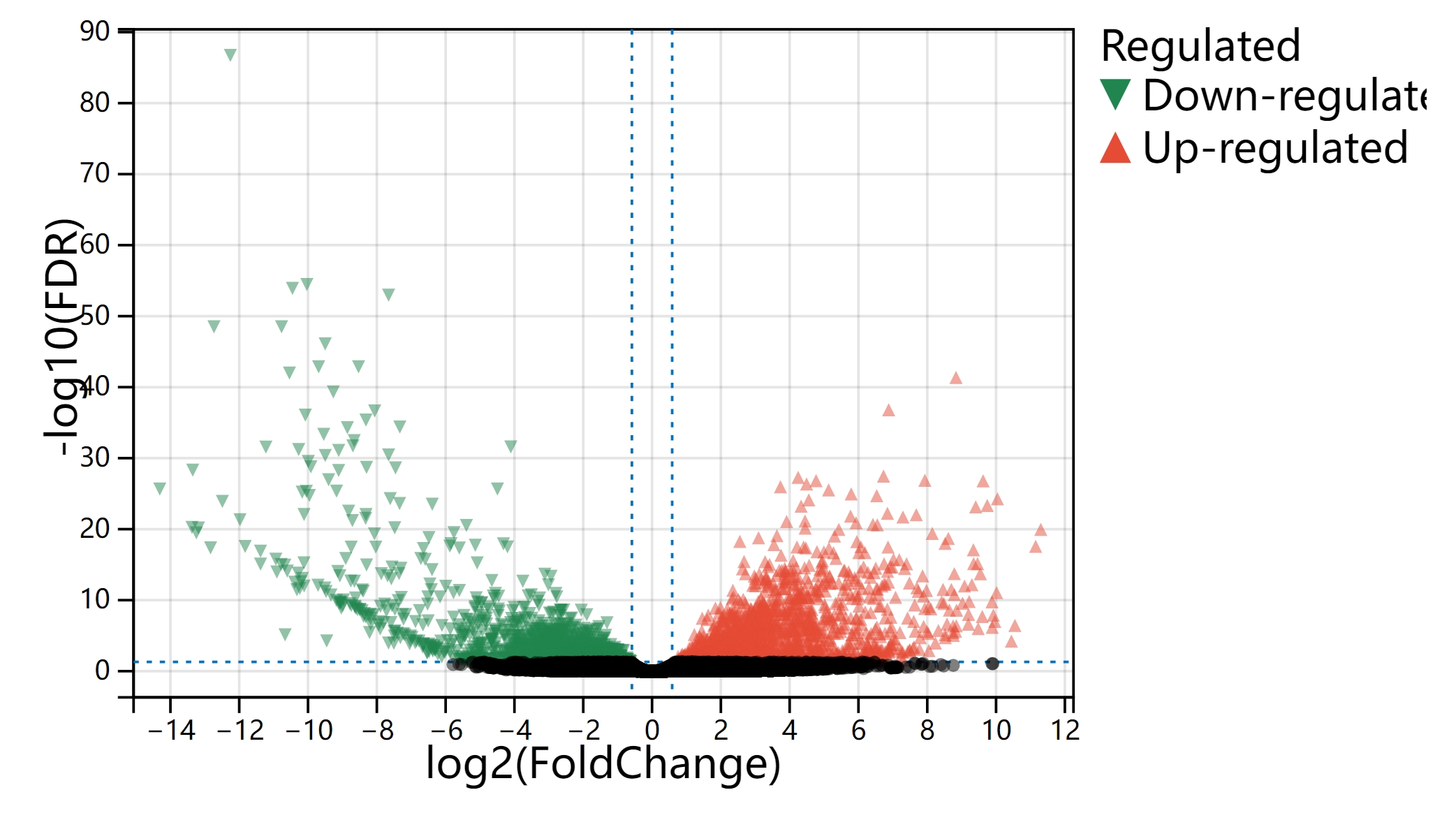

Supplement: Supplementary file 3 [file Data_Sheet_2.zip › Source data/FIGURE 4/result-m&j-chuli .jpeg]

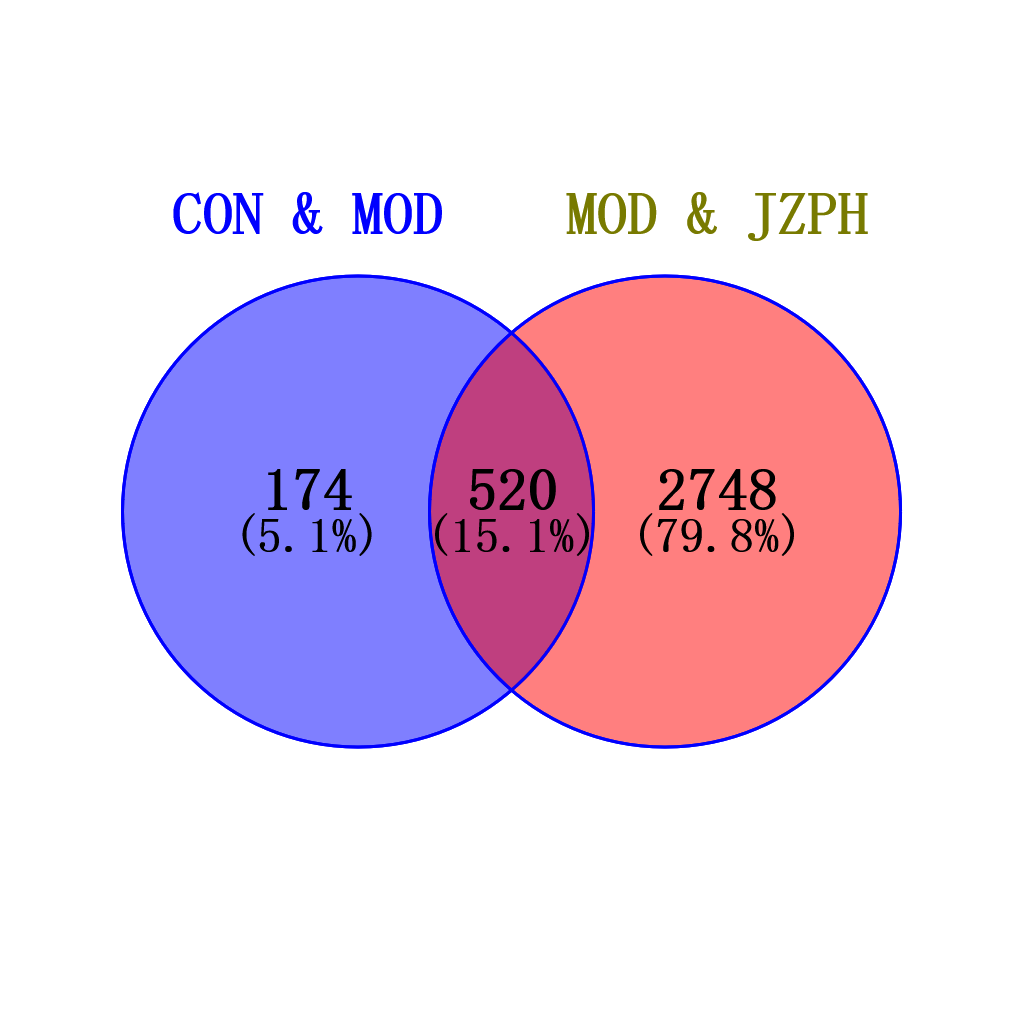

Supplement: Supplementary file 3 [file Data_Sheet_2.zip › Source data/FIGURE 4/交集.png]

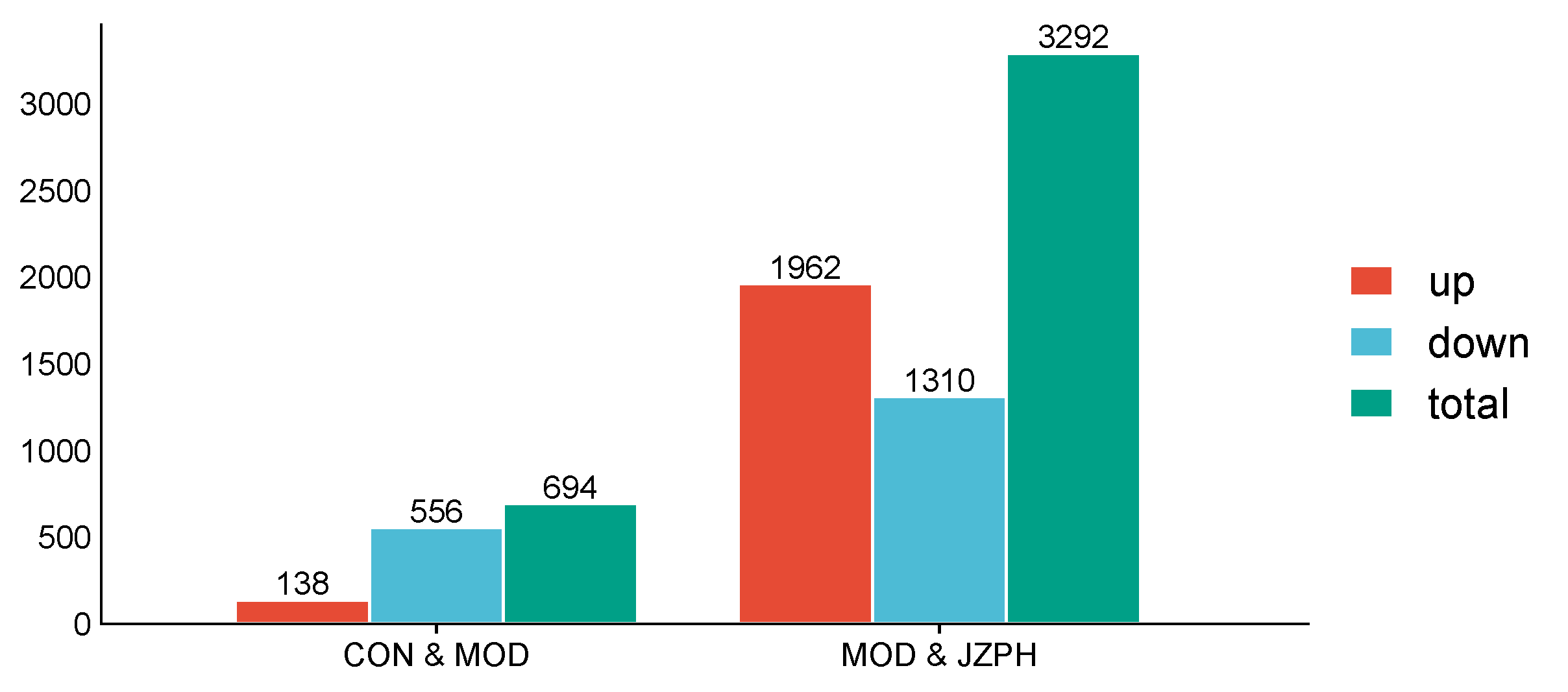

Supplement: Supplementary file 3 [file Data_Sheet_2.zip › Source data/FIGURE 4/柱状图汇总.png]

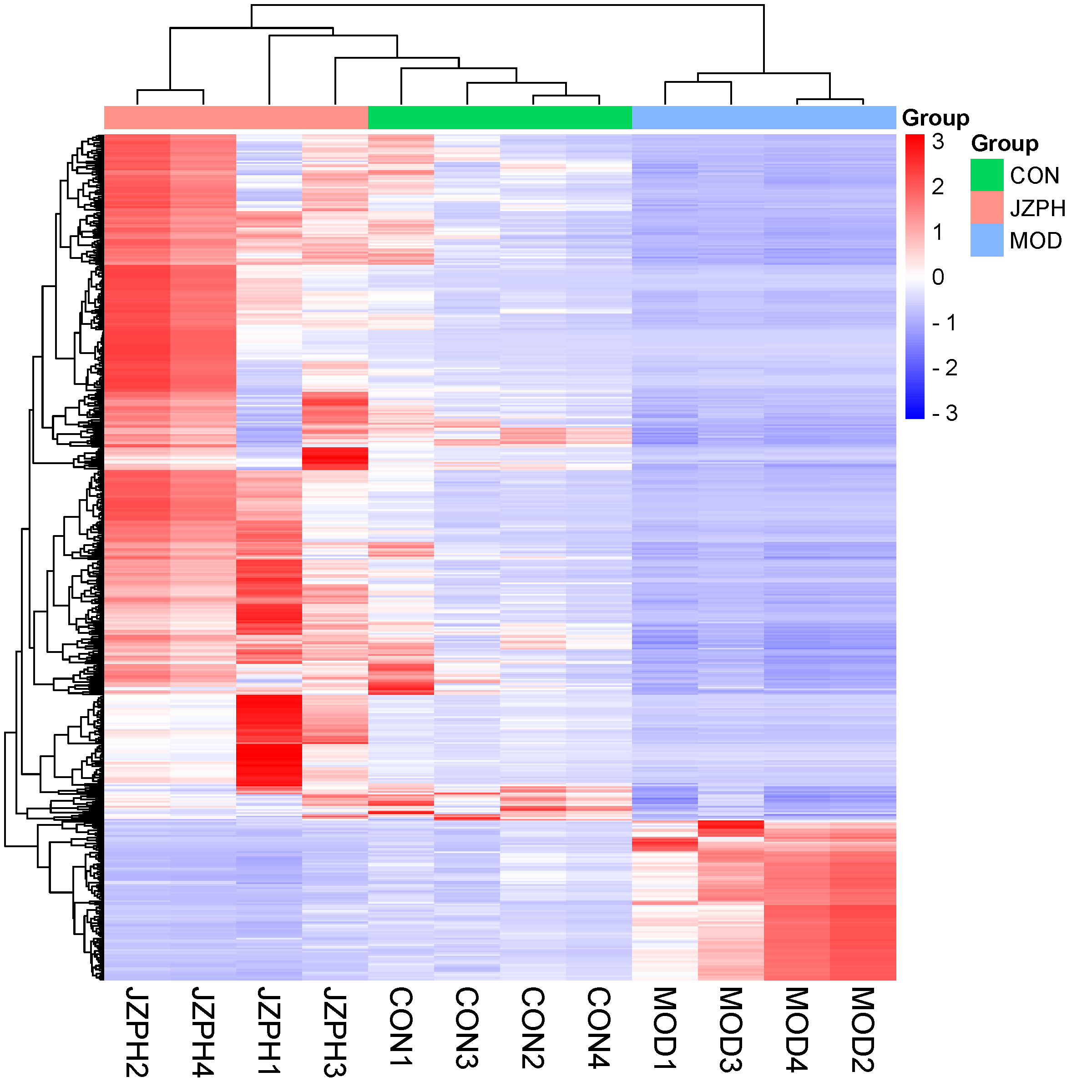

Supplement: Supplementary file 3 [file Data_Sheet_2.zip › Source data/FIGURE 4/聚类图.png]

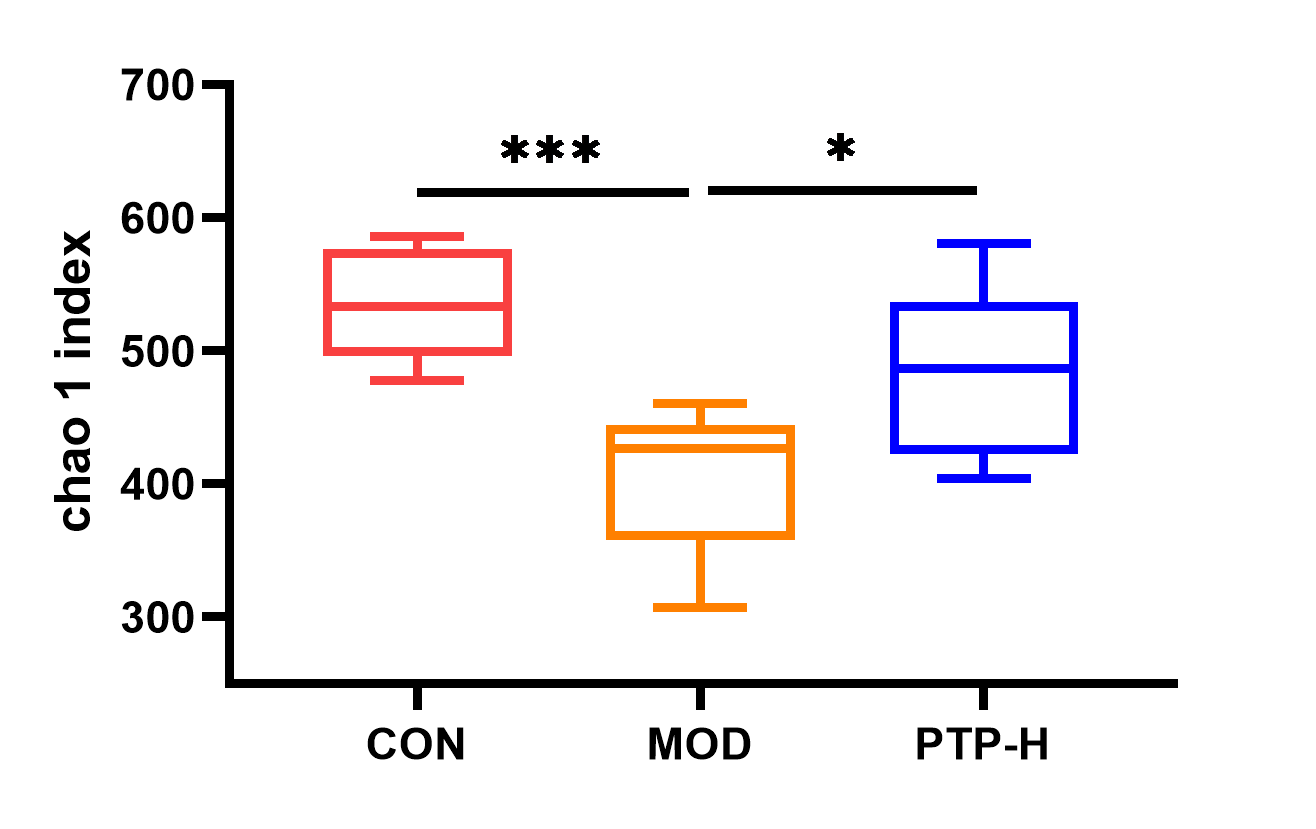

Supplement: Supplementary file 3 [file Data_Sheet_2.zip › Source data/FIGURE 5/chao 1 index.png]

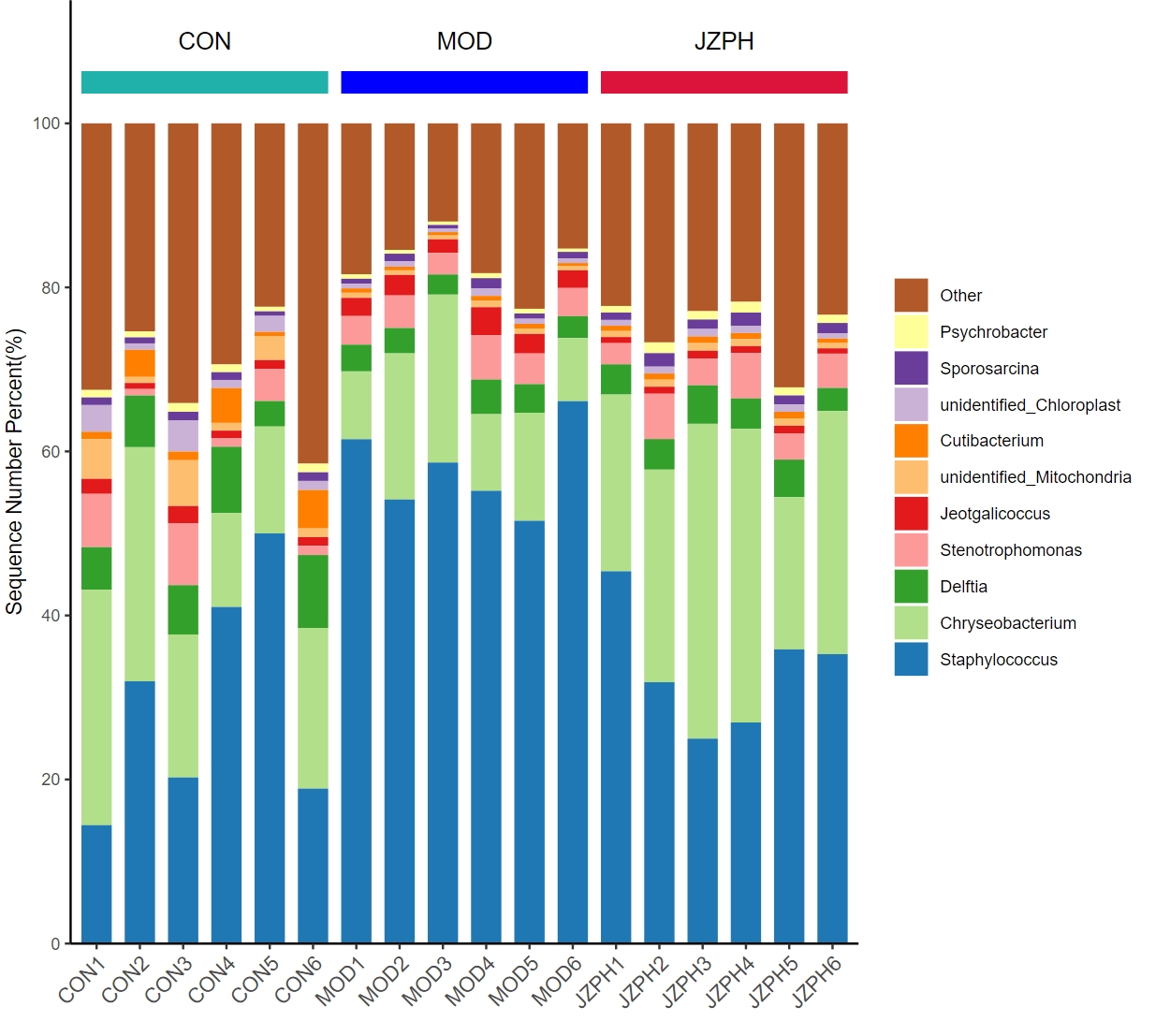

Supplement: Supplementary file 3 [file Data_Sheet_2.zip › Source data/FIGURE 5/g 丰度.jpeg]

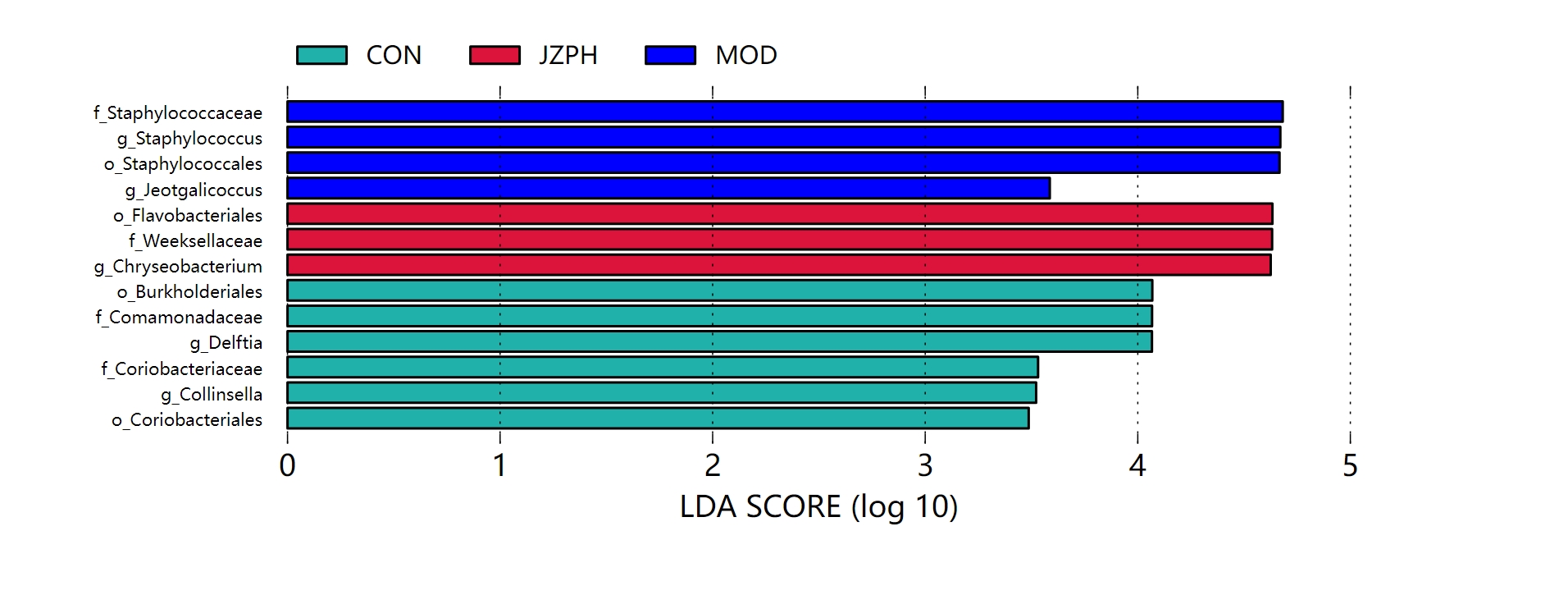

Supplement: Supplementary file 3 [file Data_Sheet_2.zip › Source data/FIGURE 5/lefse.jpeg]

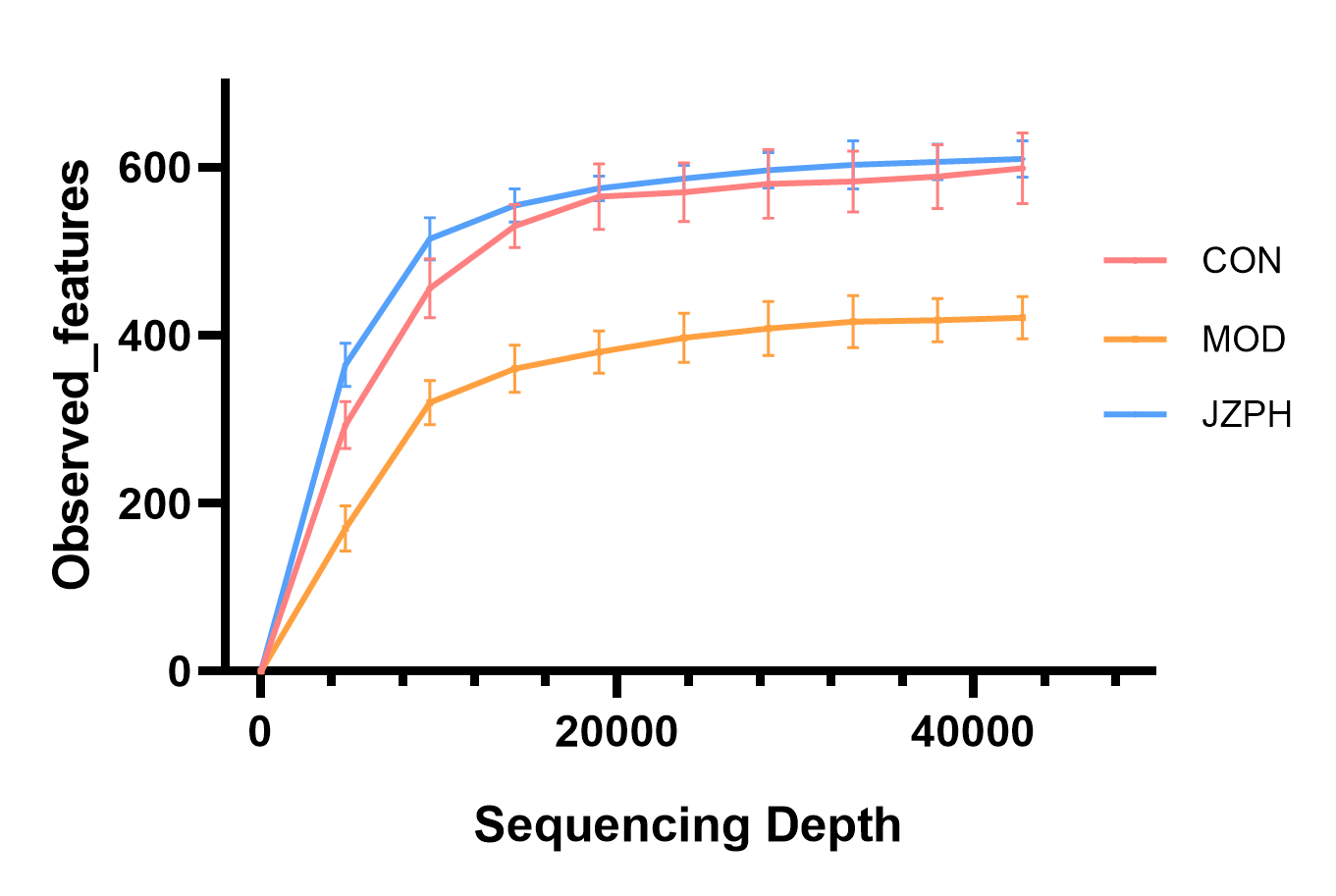

Supplement: Supplementary file 3 [file Data_Sheet_2.zip › Source data/FIGURE 5/Observed_features.png]

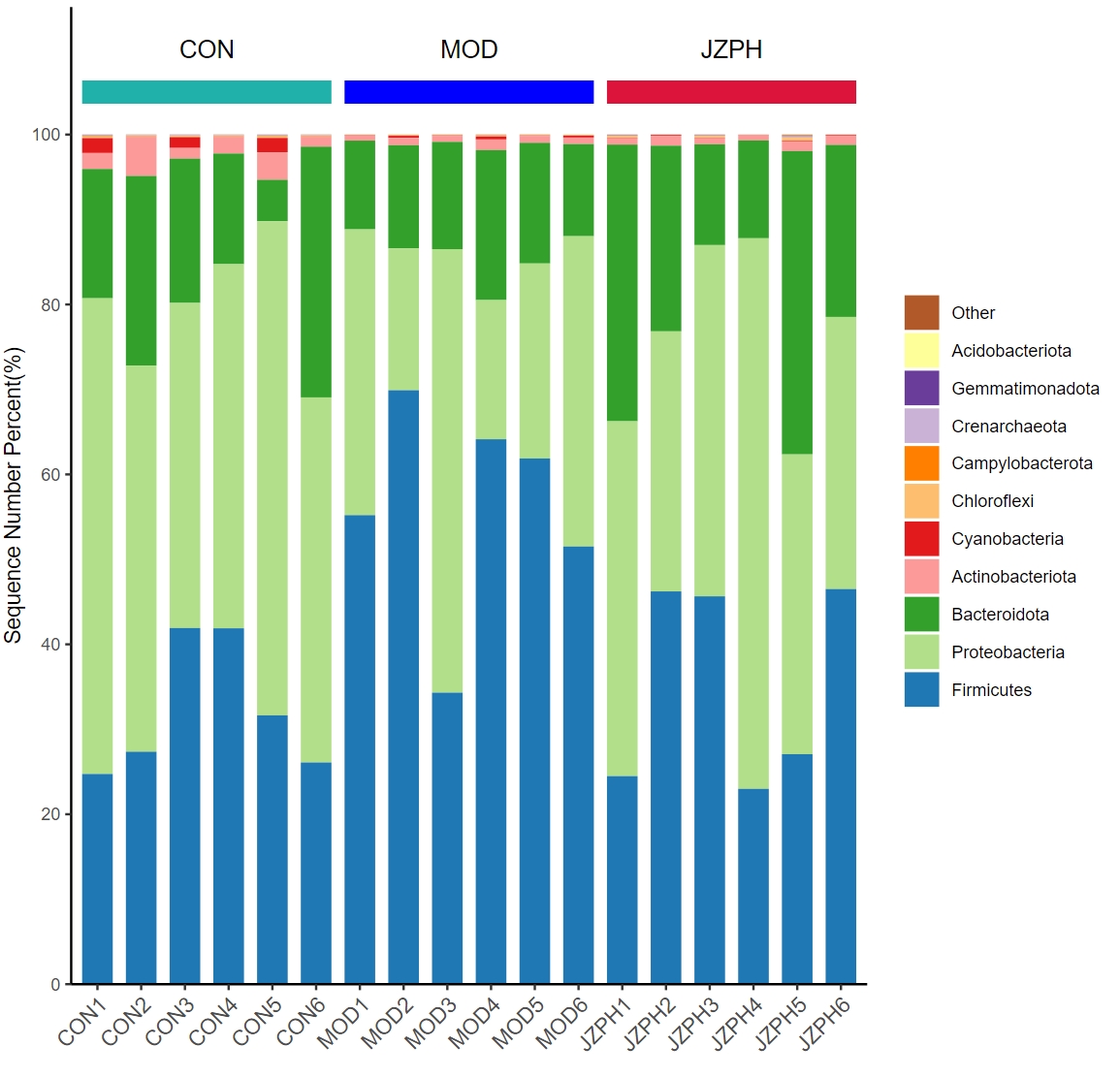

Supplement: Supplementary file 3 [file Data_Sheet_2.zip › Source data/FIGURE 5/p 丰度.jpeg]

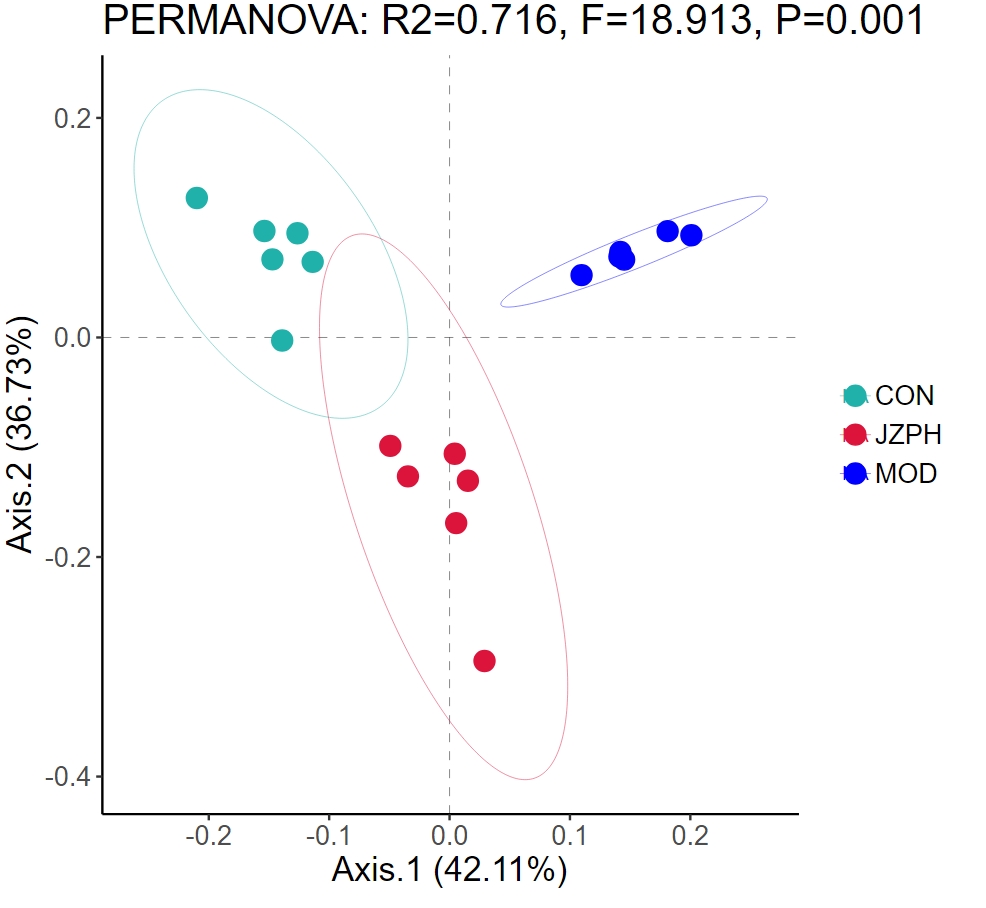

Supplement: Supplementary file 3 [file Data_Sheet_2.zip › Source data/FIGURE 5/PCoA.jpeg]

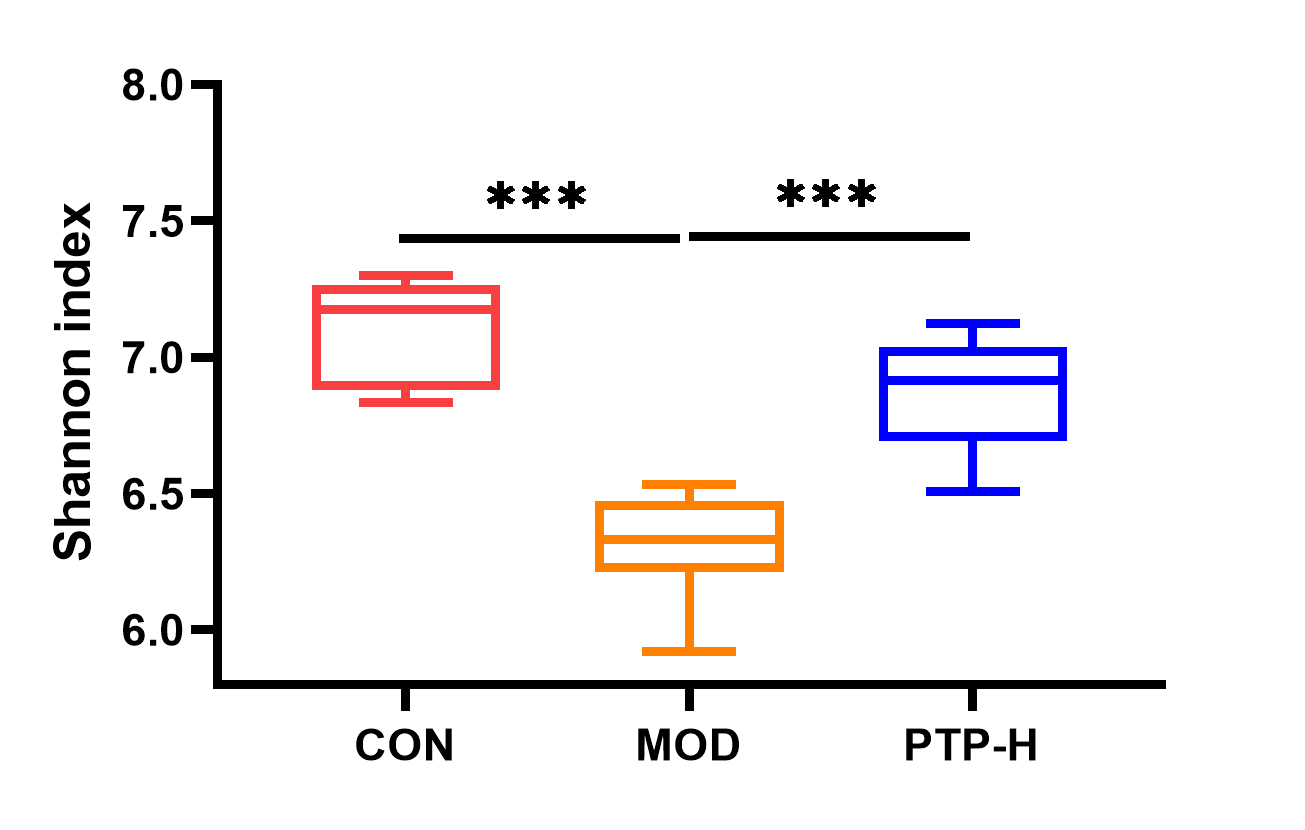

Supplement: Supplementary file 3 [file Data_Sheet_2.zip › Source data/FIGURE 5/Shannon index.png]

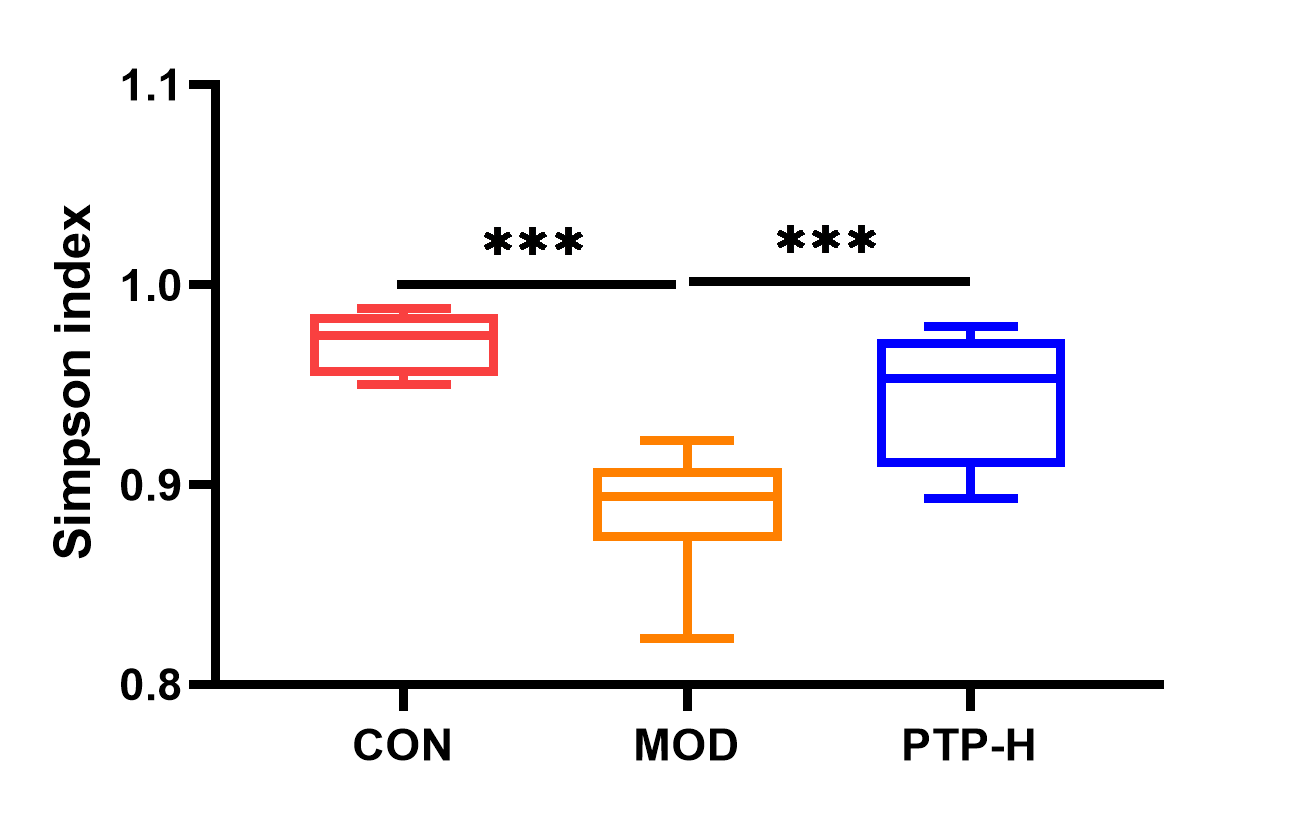

Supplement: Supplementary file 3 [file Data_Sheet_2.zip › Source data/FIGURE 5/Simpson index.png]

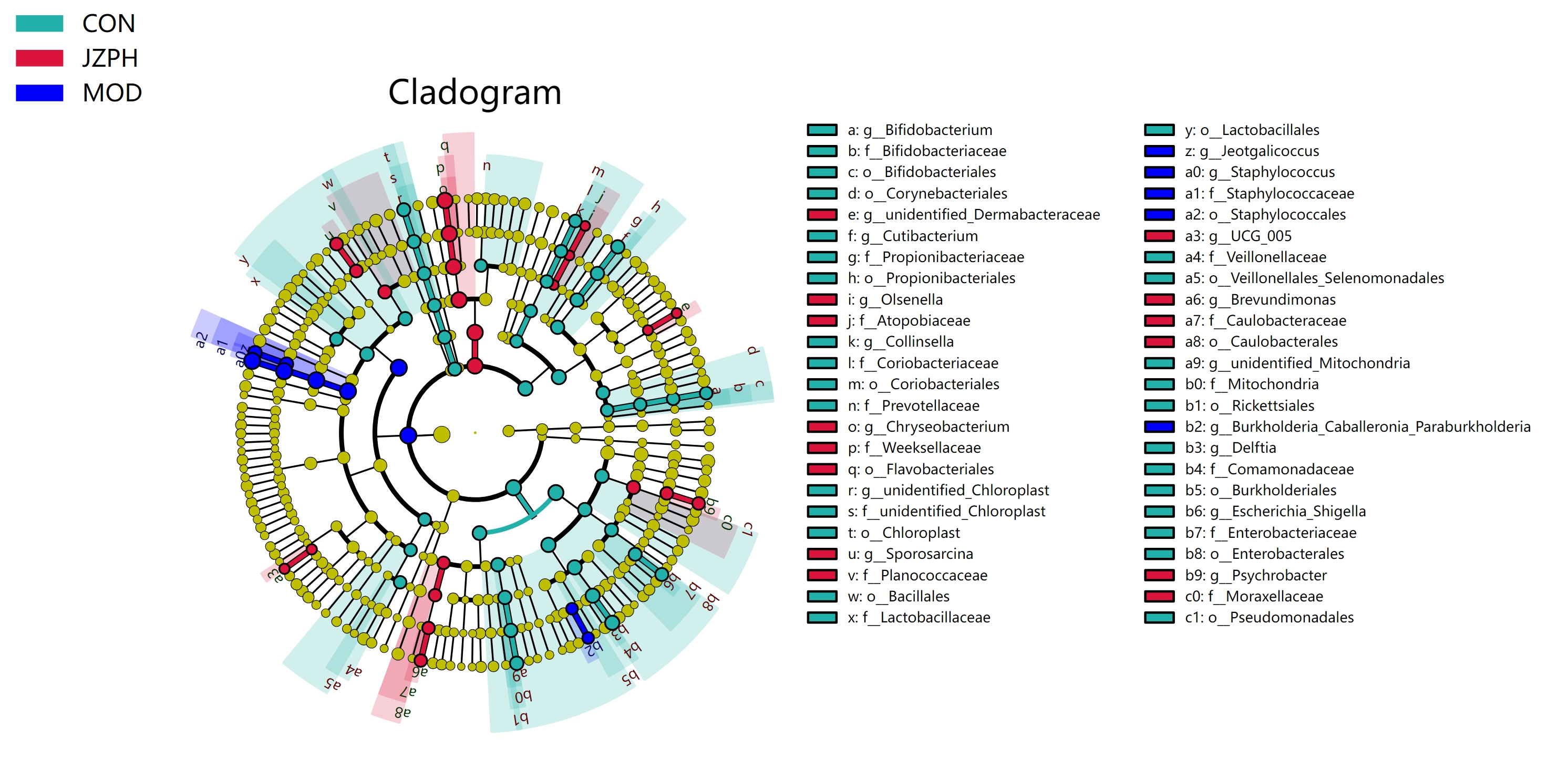

Supplement: Supplementary file 3 [file Data_Sheet_2.zip › Source data/FIGURE 5/进化树.jpeg]
